# Welcoming Neighbour or Inhospitable Host? Selective Second Metal Binding in 5- and 6-Phospha-Substituted Bpy Ligands

**DOI:** 10.3390/molecules29051150

**Published:** 2024-03-05

**Authors:** James A. Platts, Benson M. Kariuki, Paul D. Newman

**Affiliations:** School of Chemistry, Cardiff University, Cardiff CF10 3AT, UK; platts@cardiff.ac.uk (J.A.P.); kariukib@cardiff.ac.uk (B.M.K.)

**Keywords:** heterobimetallic complexes, phospha–bpy ligands, rotamers

## Abstract

The controlled formation of mixed-metal bimetallics was realised through use of a *fac*-[Re(CO)_3_(*N*,*N*′-bpy-P)Cl] complex bearing an exogenous 2,4,6-trioxa-1,3,5,7-tetramethyl-8-phosphaadamantane donor at the 5-position of the bpy. The introduction of gold, silver, and rhodium with appropriate secondary ligands was readily achieved from established starting materials. Restricted rotation about the C_(bpy)_-P bond was observed in several of the bimetallic complexes and correlated with the relative steric bulk of the second metal moiety. Related chemistry with the 6-substituted derivative proved more limited in scope with only the bimetallic Re/Au complex being isolated.

## 1. Introduction

Bimetallic complexes, especially those containing disparate metal ions, are becoming increasingly important molecular platforms in an array of applications including catalysis [1,2,3,4,5,6,7,8], medical imaging and therapy [9,10,11,12,13,14,15], and optoelectronics [16,17,18,19]. The association between the two metals can be intimate, involving direct M–M’ bonds, or be remote, with each metal occupying quite separate accommodations within the same molecule. The controlled construction of such systems requires highly selective donor groups for each particular metal ion and/or the development of astute synthetic procedures for the deliberate sequential introduction of the metals [20,21,22,23,24,25,26,27,28,29,30,31]. Our approach is based on the former, with different donor sets (2 × N of bpy and 1 × P) providing the necessary discrimination. Examples of ligands of this type with phosphine functions bonded directly to one or more positions on the pyridyl ring(s) in bpy are surprisingly rare [32,33], with only one known example involving the [Re(CO)_3_X] core [33].

The lack of precedence in the literature regarding metal complexes of 2,2′-bpy derivatives adorned with a phosphine donor(s) piqued our interest in exploring these systems as potential platforms for supporting heterobimetallics. The ultimate goal is to study mechanisms of electron and/or energy transfer between two unique metal centres within a single molecule and their application as theranostics and/or as metallaphotoredox catalysts. The combination of a photo-active rhenium centre and a carbonylation catalyst in a single molecule is an attractive prospect as it can enable transformations utilising CO_2_ as the source of CO [34,35,36]. To this end, we report here our recent efforts on the development of heterobimetallic complexes combining a light-responsive unit and a second metal fragment. The current manuscript describes studies of 5- and 6-phosphatrioxaadamantane substituted 2,2′-bipyridine ligands and their ability to accommodate a [Re(CO)_3_Cl] core (bpy donor) and group 9, 10, or 11 metal ions (P-donor). The primary aim was to identify limitations on second metal coordination associated with the positioning of the phosphine donor on the 2,2′-bpy framework, specifically determining distinctions in the coordination behaviour of 5- and 6-phosphino derivatives.

## 2. Results and Discussion

### Ligand Synthesis

The phosphine-derivatised bpy compounds CgP-5bpy (**5L**) and CgP-6bpy (**6L**) are ditopic ligands designed to selectively bind two disparate metal centres where one metal can potentially act as a light-activated reporter/activator and the other as a therapeutic or catalyst. The 2,4,6-trioxa-1,3,5,7-tetramethyl-8-phosphaadamantane (PCg) unit was chosen as the phosphine function as it is rigid and bulky with a cone angle between that of ^t^Bu_2_P and PPh_2_. The air-stability of many of its known derivatives was perceived to be a further advantage and it has an established coordination chemistry with the metals of interest in this study [37,38,39,40]. The bpy unit was chosen as complexes of this ligand with appropriate transition metal cores, notably those based on rhenium, ruthenium, or iridium, are renowned photo-active systems for multifold applications.

**Scheme 1:**
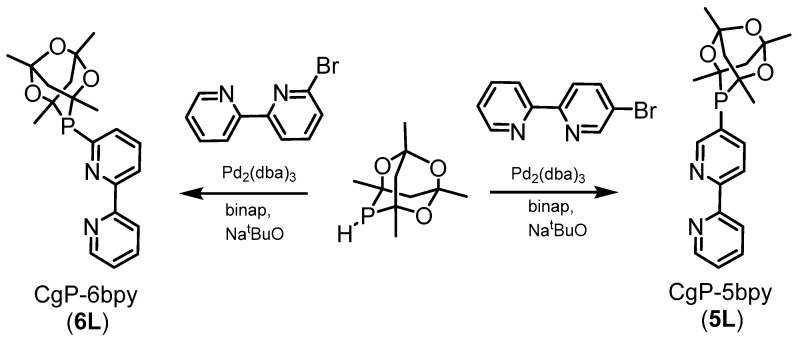
Synthesis of the ligands CgP-5bpy (**5L**) and CgP-6bpy (**6L**).

The ligands were synthesised by the C–P coupling chemistry highlighted in Scheme 1. In contrast to many derivatives, including the pyr–PCg analogue [41], **5L** proved to be air-sensitive and the procedure had to be performed under strictly anaerobic conditions. Although far less susceptible to air oxidation in the solid state, as a precaution, the isolated white solid was stored under nitrogen. A single peak at δ_P_ −30.5 ppm was seen in the ^31^P{^1^H} NMR spectrum of **5L** and all other spectroscopic data were in accord with the expected structure (see Supplementary Materials). Unlike **5L**, **6L** proved to be air-stable with no discernible oxidation being observed upon leaving non-degassed NMR samples in air for several weeks, as determined by the constant presence of a single peak at δ_P_ −25 ppm and the absence of any other signals in the ^31^P{^1^H} NMR spectrum of the compound. In both cases, there was no evidence of rotamers reflecting free rotation about the C_bpy_–P bond on the NMR timescale.

**κ^2^-*N,N*′-rhenium complexes:** The preparation of the rhenium complex *fac*-[Re(CO)_3_(κ^2^-*N*,*N*′-**5L**)Cl] (**5L^Re^**) was achieved through heating a 1:1 mixture of [Re(CO)_5_Cl] and **5L** in degassed PhCl for several hours under nitrogen. After the removal of all volatiles, the yellow, air-sensitive complex was acquired in good yield and of sufficient purity for all subsequent reactions (>90%). Under the same reaction conditions, selective coordination of the bpy fragment was observed as determined by the lack of a coordination shift in the ^31^P{^1^H} NMR spectrum; this agrees with similar observations from the group of Bercaw on a 6-substituted diphenylphosphino derivative prepared under similar conditions [33]. Although no significant change in the chemical shift was observed, two signals of almost equal intensity were seen at δ_P_ −31.5 and −31.6 ppm in the ^31^P{^1^H} spectrum of **5L^Re^**, reflecting the presence of rotamers resulting from restricted rotation about the C_bpy_–P bond (restricted rotation has previously been observed with cagephos complexes) [42]. This was confirmed upon inspection of the ^1^H and ^13^C{^1^H} NMR spectra, which showed distinct duplication of peaks for selected resonances (notably those hydrogen atoms in the immediate proximity of the C–P bond). The expected coordination shifts for the aromatic protons were noted, with the largest of these occurring for the 6,6′ hydrogens.

**Figure 1:**
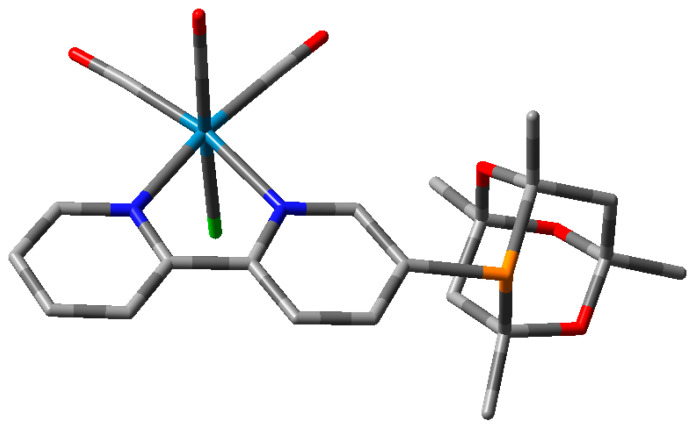
Optimised geometry of **5L^Re^** (hydrogens omitted for clarity). Light blue = Re; dark blue = N; green = Cl; red = O; orange = P.

The presence of rotamers was explored using DFT for both the uncoordinated ligand and the rhenium complex. **5L** has two equivalent low-energy (and two metastable) conformers at C–P–C–C torsion angles of 0 and 180°, whereas the rhenium complex has non-equivalent low-energy conformers with the same torsion angles as the ligand, with the lowest energy conformer being the one with a C–P–C–C angle of 0⁰ and the lone pair on P pointing away from the Re(CO)_3_ group (Figure 1). The two rotamers of **5L^Re^** did not interconvert in *d*_6_-acetone or *d*_6_-dmso when left under ambient conditions for several weeks. However, an unrelated change was evident upon leaving these samples over extended periods of time with the emergence of two new peaks at δ_P_ 16.4 and 16.3 and 16.8 and 16.6 ppm in *d*_6_-acetone and *d*_6_-dmso, respectively. These peaks developed at the expense of those around δ_P_ −32 ppm and resulted from the coordination of the phosphine donor to a second rhenium centre (presumably through substitution of the chloride) to form a dirhenium complex in solution. A similar change was observed in the ^1^H spectrum recorded in C_6_D_6_, although the conversion was noticeably slower. This was not the result of oxidation, as the oxidised compound has a ^31^P{^1^H} chemical shift at δ_P_ 22.1 ppm.

Formation of the rhenium complex *fac*-[Re(CO)_3_(κ^2^-*N,N*′-**6L**)Cl] (**6L^Re^**) was achieved in a similar manner to above by heating a 1:1 mixture of [Re(CO)_5_Cl] and the ligand in PhCl for several hours. The isolated bright yellow solid proved to be poorly soluble to insoluble in most common organic solvents but did dissolve in dmso upon warming the sample. As noted for **5L^Re^**, two isomers of **6L^Re^** were observed by NMR spectroscopy, although a clear preference for one was evident from the 4:1 ratio. The major isomer is most likely that presenting the bulky phosphacycle away from the [Re(CO)_3_Cl] core. The aromatic hydrogens show the expected downfield coordination shifts as exemplified by the 6,6′ protons resonating around 0.5 ppm downfield of their position in the ^1^H NMR spectrum of the uncoordinated ligand.

Unlike the CgP-5bpy derivative, there was no evidence of self-dimerisation through coordination of the phosphine from one complex to another for **6L^Re^**. As might be expected, this suggests that the phosphine is more available to act as a ligand to a second metal in **5L^Re^** compared to **6L^Re^**. In order to test this further, the in situ reaction of both with one equivalent of [Re(CO)_5_Cl] was explored. Due to limitations with solubility of the 6-derivative, the reactions were performed in d_6_-dmso and assessed by ^31^P{^1^H} NMR spectroscopy. Heating the *fac*-[Re(CO)_3_(**5L**)Cl]/[Re(CO)_5_Cl] mixture to ~70 °C led to the formation of *fac*-[Re(CO)_3_(κ-*N,N*′-Re-κ-*P*-Re’-**5L**)Cl{Re’(CO)_4_Cl}] within an hour as evidenced by the complete loss of the peaks for the starting material and the subsequent appearance of two new peaks at δ_P_ −7.6 and −8.2 ppm. Only the starting material was observed in the ^31^P{^1^H} NMR spectrum of the *fac*-[Re(CO)_3_(κ-*N,N*′-**6L**)Cl]/[Re(CO)_5_Cl] mixture even after heating at ~100 °C for 24 h; this serves to emphasise inherent limitations to binuclear complex formation for *fac*-[Re(CO)_3_(**6L**)Cl] through steric inhibition.

**Heterometallic Re/Au complexes:** As the [Re(CO)_4_Cl] fragment was not coordinated by the P-donor of **6L^Re^**, a smaller metal unit was sought to further assess steric limitations. As Au(I) strongly prefers a linear, two-coordinate geometry, the sterically small AuCl group was chosen to promote the formation of the bimetallic complex. The synthesis of *fac*-[Re(*κ*^2^-*N,N*′-Re,*κ*-*P*-Au**-5L**)(CO)_3_Cl(AuCl)] (**5L^Re,Au^**) was readily achieved upon mixing equimolar amounts of **5L^Re^** and Au(THT)Cl in dichloromethane at room temperature. The orange solid obtained after the removal of volatiles was air-stable and could be recrystallised by vapour diffusion of pentane into an acetone solution of the complex. The rotational isomerism seen in the rhenium parent extended to the **5L^Re,Au^** complex, as evidenced by the presence of two peaks in a 45:55 ratio at δ_P_ 20.9 and 20.5 ppm in the ^31^P{^1^H} NMR spectrum; coordination shifts of 52 ppm are comparable with related complexes [39]. This was also evident from selected peaks in the ^1^H NMR spectrum, most notably the two doublets at δ_H_ 1.68 (^2^*J*_H-P_ = 15.4 Hz) and 1.61 (^2^*J*_H-P_ = 15.4 Hz) ppm for two of the methyls of the CgP structure. The HRMS shows the parent peak at 873.0209 amu.

The C_bpy_–P rotational barrier for the gold complex showed two non-equivalent energy minima (see Supplementary Materials and discussion below) at C–P–C–C values of 80° (relative energy: zero) and 250° (rel. energy: +27 kJ mol^−1^). The barriers to interconversion were 51 and 24 kJ mol^−1^. This latter value suggested that a single isomer could be isolated under appropriate conditions. It was noticed that an NMR sample left for several days at RT showed a change in the relative intensity of the two rotamers and, hence, the presence of a solution equilibrium favouring the more stable form. The NMR sample was left to evaporate slowly over several weeks and the solid obtained redissolved in CDCl_3_. An analysis of the ^1^H and ^31^P{^1^H} NMR spectra of the recovered solid showed a single isomer (see Supplementary Materials).

**Figure 2:**
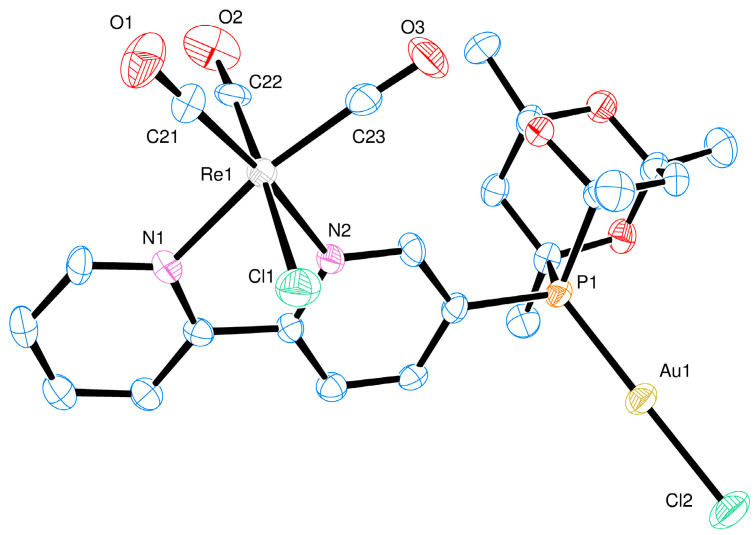
Ortep [45] representation of the molecular structure of **5L^Re,Au^**. Hydrogen atoms and residual solvent have been omitted for clarity. Selected bond lengths (Å) and angles (°): Re1—N1 2.170(5); Re1—N2 2.162(4); Re1—C21 1.925(6); Re1—C22 1.973(6); Re1—C23 1.924(6); Re1—Cl1 2.4483(16); Au1—P1 2.2177(14); Au1—Cl2 2.2786(15); N1—Re1—C23 170.1(2); N2—Re1—C21 171.9(2); Cl1—Re1—C22 176.42(17); P1—Au1—Cl2 179.34(7).

The molecular structure of **5L^Re,Au^**, along with pertinent metrics, determined by SCXRD is shown in Figure 2. Both metal ions show their anticipated geometry and the AuCl fragment is directed away (*anti*) from the [Re(CO)_3_] core with the P–Au–Cl vector projecting at an angle of 131.7° with respect to the pyridine ring and orientated on the side of the Re(bpy)(CO)_2_ plane that contains the rhenium-bound chloride. This places the main bulk of the CgP unit in the region of space proximate to the CO group *trans* to the chloride, resulting in some distortion of the Re–C–O linkage to 169.7°. At 2.2177(14) Å, the Au–P bond length is in line with values seen in similar compounds, including the related complex of Smith et al. (2.2328(16), 2.280(2)) [39] and 2.2285(8) [43] and 2.2137(18) Å for those of Stradiotto and Newman, respectively [44]. It is noteworthy that the preferred geometry/configuration determined by DFT agrees with that shown in Figure 2.

**Figure 3:**
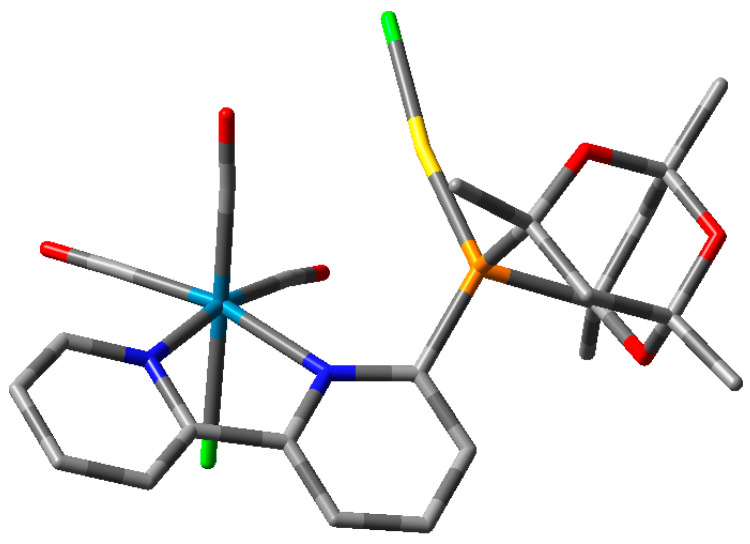
Geometry optimised molecular structure of **6L^Re,Au^**. Light blue = Re; dark blue = N; green = Cl; red = O; orange = P; yellow = Au.

The poor solubility of **6L^Re^** in common organic solvents proved problematic for the formation of the bimetallic complexes. Formation of the gold complex was achieved through the use of a CH_2_Cl_2_/dmso solvent mixture or by employing a relatively large volume of solely CH_2_Cl_2_. Once formed, the mixed rhenium/gold complex proved more soluble in organic solvents than the rhenium complex and, unlike the parent complex, there was no evidence of rotameric isomerism in *fac*-[Re(CO)_3_(κ^2^-*N*,*N*′-κ-P^Au^-**6L**)Cl(AuCl)] (**6L^Re,Au^**). A single peak was observed in the ^31^P{^1^H} NMR spectrum at δ_P_ 22.5 ppm and no duplication of peaks occurred in the ^1^H or ^13^C{^1^H} NMR spectra. Although crystals suitable for SCXRD were not forthcoming, the theoretically derived structure had both the gold and rhenium atoms as mutually *cis* (see below and Figure 3). This reflects a requirement to station the bulky phosphacycle away from the rhenium core and is achievable with the sterically small AuCl unit (albeit with some necessary distortion, as noted below).

**Heterometallic Re/Ag complexes:** Coordination control in Ag(I) complexes is not as readily achieved as for Au(I). No attempt was made to isolate the silver complex *fac*-[Re(CO)_3_(κ^2^-*N,N*′-Re-κ-*P-*Ag-**5L**)Cl(AgOTf)], although an in situ reaction was performed on an NMR scale using **5L^Re^** and one equivalent of AgOTf in CD_3_CN. The only signal observed in the ^31^P{^1^H} NMR spectrum of this mixture was a broad resonance centred around δ_P_ −9.3 ppm with a width at half height of ~240 Hz. The in situ ^1^H spectrum was also broadened and showed evidence of two isomers along with a third minor product. Although the broadening was indicative of the formation of a silver complex, no attempt was made to isolate this complex. A subsequent preparative scale reaction between **5L^Re^** and 0.5 mol equivalents of AgOTf led to the isolation of an orange complex that was recrystallised from acetone by the slow introduction of pentane. The ^31^P{^1^H} NMR spectrum was again broad, with a peak maximum at δ_P_ −16.0 ppm and clear evidence of two species, as the peak was unsymmetrical with an obvious shoulder. The ^1^H NMR spectrum was not broad and assignments could be made based upon a series of 1D and 2D NMR spectra (see Supplementary Materials). All attempts to isolate a similar complex starting from **6L^Re^** were met with failure, partly as a result of the poor solubility of the rhenium complex in common organic solvents but also the hindered nature of the P-donor in this system. Attempts to access the silver complex by prior synthesis of the P-bound silver complex [Ag(κ-*P*-**6L**)_2_]BF_4_ and subsequent coordination to rhenium were also frustrated, as the reaction of this pre-formed complex with [Re(CO)_5_Cl] gave largely intractable materials with no evidence of P-bound silver; the ^31^P{^1^H} NMR spectrum of an isolated yellow solid mimicked that observed for **6L^Re^**.

**Heterometallic Re/Rh complexes:** In situ monitoring of the reaction of **5L^Re^** with 0.5 equivalents of [Rh(COD)Cl]_2_ revealed the rapid formation of two rotameric isomers of *fac*-[Re(CO)_3_(κ^2^-*N,N*′-Re-κ-*P-*Rh-**5L**)Cl(Rh(COD)Cl)] (**5L^Re,Rh^**) represented by two doublets in a 1:1 ratio at δ_P_ 7.18 (^1^*J*_P-Rh_ 153 Hz) and 6.93 (^1^*J*_P-Rh_ 152.6 Hz) ppm in the ^31^P{^1^H} NMR spectrum. After isolating the mixture in good yield, attempts were made to obtain a single isomer by recrystallisation. Isomeric enrichment was observed with several solvent mixtures but only toluene gave a single isomer, as evidenced by the presence of one doublet, δ_P_ 6.94 ppm with ^1^*J*_P-Rh_ = 156.0 Hz, in the ^31^P{^1^H} NMR spectrum of the isolated complex. The ^1^H NMR spectrum confirmed the presence of a single isomer with seven distinct aromatic peaks for the bpy fragment and characteristic alkenic hydrogens of the coordinated COD ligand between δ_H_ 4.5 and 5.5 ppm.

**Figure 4:**
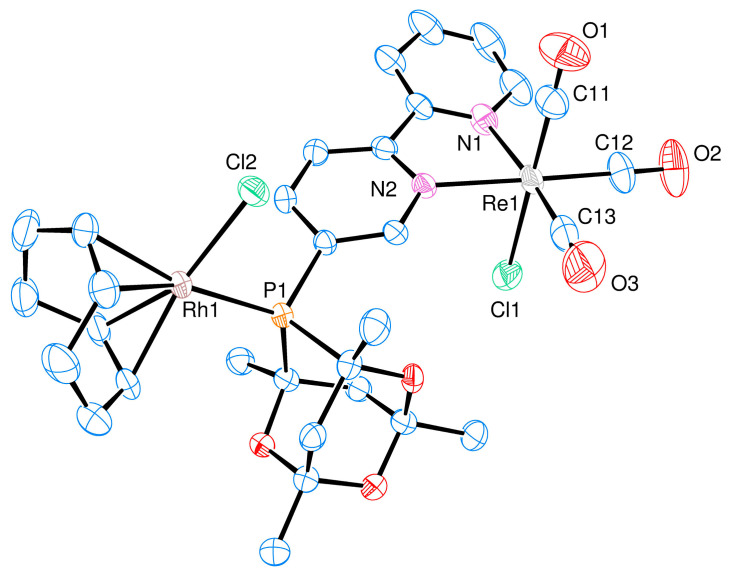
Ortep [45] representation of the molecular structure of *anti*-**5L^Re,Rh^**. Hydrogen atoms and residual solvent have been omitted for clarity. Selected bond lengths (Å) and angles (°): Re1—N1 2.173(3); Re1—N2 2.172(3); Re1—C11 1.902(6); Re1—C12 1.910(4); Re1—C13 1.908(5); Re1—Cl1 2.4563(18); Rh1—P1 2.3176(8); Rh1—Cl2 2.3580(8); N1—Re1—C13 171.67(14); N2—Re1—C12 173.15(15); Cl1—Re1—C11 179.1(2); P1—Rh1—Cl2 84.48(3).

Unfortunately, crystals suitable for SCXRD could not be obtained from toluene but were accessed by the slow evaporation of diethyl ether into an acetone solution of the isomeric mixture of the complex. An analysis of the crystallographic data revealed the presence of two isomeric forms differentiated by the relative position of the rhenium-bound chloride with respect to the rhodium atom in a 78:22 ratio. Figure 4 shows the structure of the major *anti* isomer with the chloride attached to rhenium being on the opposite side of the Re(bpy) plane to the rhodium. In the second (minor) isomer, the positions of the chloride bound to rhenium and the *trans* CO are swapped to give the *syn* isomer. DFT predicts this isomer with Re–Cl and Rh–Cl in *anti* orientation as the global minimum, with the other rotamer being 9.8 kJ mol^−1^ higher in energy.

Given that papers concerned with rhodium–phosphine complexes are legion, the number of reported complexes of CgP derivatives is surprisingly small and the direct comparison of metric features is hence somewhat limited, to the extent that we were unable to locate SCXRD data for any [Rh(COD)(PR_3_)Cl] complex where the phosphine is of the current type. Although not directly comparable, the Rh–P bond length of 2.3176(8) Å observed for **5L^Re,Rh^** is shorter than the 2.3205(7) Å and 2.3245 (av.) seen in related [Rh(L)_2_CO(Cl)] complexes [44,46]. The Re–N bond lengths are equivalent, reflecting both a lack of steric impedance and little electronic influence of the 5-phospha/rhodium unit. The only noticeable distortion of the metrics about the rhenium centre is for one of the Re–C–O angles, which is slightly compressed to 172(3)° due to the presence of the phosphacycle.

The aforementioned synthetic route to **5L^Re,Rh^** was not successful for **6L^Re,Rh^**, with only the undissolved starting material being recovered. In an effort to circumvent this, the P-bound rhodium complex [Rh(COD)(*κ*-P-**6L**)Cl] (**6L^Rh^**) was prepared prior to attempting coordination of the bpy fragment to rhenium. The synthesis of **6L^Rh^** was readily achieved upon mixing the two components in a 1:1 ratio in dichloromethane. All spectroscopic data were consistent with the expected formulation. A subsequent reaction of **6L^Rh^** with [Re(CO)_5_Cl] in PhCl led to the precipitation of a purple solid, which was isolated by filtration. The solid was poorly soluble in CDCl_3_ and *d*_6_-acetone, so the spectra were recorded in *d*_6_-dmso. Upon dissolution, the purple colour dissipated, leaving an orange solution. Examination of the ^31^P{^1^H} NMR spectrum showed a broad doublet at δ_P_ 31.4 ppm, with a ^1^*J*_P-Rh_ coupling constant of 149 Hz. The ^1^H NMR spectrum only showed resonances due to the **L6** ligand, with no evidence of 1,5-COD. The LRMS spectrum showed peaks for [Re(CO)_3_(**6L**)]^+^ at 641.09 amu (30%) and [Rh(**6L**)CO]^+^ at 501.05 amu (100%). It was noticeable that the original complex decomposed rapidly in solution, as evidenced by extensive changes in the NMR spectra over time. Although the exact nature of this purple complex remains elusive, it is not an analogue of **5L^Re,Rh^**, again emphasising the inhospitable nature of the 6-subsituted ligand for generating bimetallic complexes.

**Electronic spectroscopy:** The UV/Vis spectra are given in the ESI and are typical of complexes of the type [Re(CO)_3_(bpy)Cl], with an observable maximum at around 310 nm of slightly higher intensity than the maximum at ~260 nm [47]. *Fac*-[Re(CO)_3_(κ-*N,N*′-**5L**)Cl] is the exception, with enhanced intensity in the higher energy absorption band and associated structure. The increased molar absorption associated with the silver(I) complex is anticipated, given that the complex is 2:1 Re/Ag; the concentration of the chromophore is double that of the others. The emission spectra (see Supplementary Materials) of [Re(CO)_3_(κ-*N*,*N*′-**6L**)Cl] and its Au(I) complex are very similar, with maxima around 610 nm. There is less consistency in the spectra of the **5L** complexes, with the gold complex showing an emission maximum at 650 nm and the silver complex one at 590 nm. This disparity likely arises from the innate differences between the two, with the gold complex being a neutral 1:1 binuclear complex and the silver being a cationic 2:1 trinuclear complex. Direct comparison with related 6-(phospha)bpy and even 6-(amino)bpy complexes of type [Re(CO)_3_(L)Cl] is not possible due to the lack of reported spectroscopy for such analogues.

**Rotamers and comparative binding:** To further probe the rotational freedom and metal binding properties of these ligands, a series of DFT calculations (see Supplementary Materials for details) were performed. The **5L** ligand exhibits two almost degenerate minima (relative energy) corresponding to dihedral angles C6–C5–P–C of +168° (relative energy: 0.0 kJ mol^−1^) and −12° (relative energy: 1.3 kJ mol^−1^), with a barrier of 26 kJ mol^−1^ separating these. In contrast, **6L** is predicted to have one low-energy minimum with N–C6–P–C dihedral = 5°, which places the bulk of the cagephos ligand away from the bpy ring, and a higher energy-shallow minimum (relative energy: 10.1 kJ mol^−1^) with the bulky group closer to bpy. The barrier to interconversion between these forms is low at 13.8 kJ mol^−1^ relative to the global minimum.

**5L** is calculated to bind Re(CO)_3_Cl in the expected manner with equivalent Re–N bonds (2.20 and 2.21 Å) and regular octahedral coordination geometry. The rotational energy profile of the C–P bond, however, is altered upon formation of the Re complex, such that the low energy form is now 18.8 kJ mol^−1^ lower than its rotamer, with the cagephos framework and Re(CO_3_)Cl fragment separated as much as possible. Restricted rotation around the C–P bond is expected based on the barrier to rotation of 40.4 kJ mol^−1^. **6L** can also bind Re(CO)_3_Cl in two rotameric forms. In the lowest energy form, the binding is as anticipated (Re–N = 2.19 and 2.27 Å), with the cagephos ligand oriented away from the Re centre. The rotamer with the cagephos and Re(CO_3_)Cl in proximity is 86 kJ mol^−1^ higher in energy and leads to disruption in the coordination to Re with Re–N = 2.20 and 2.45 Å.

**Figure 5:**
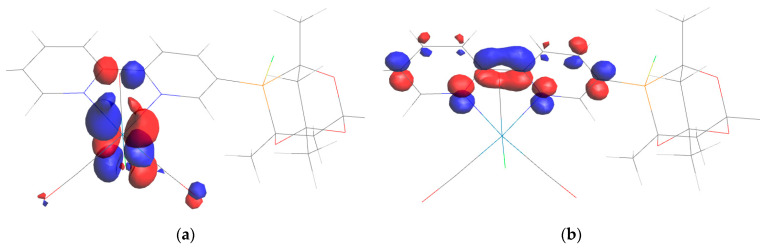
Frontier orbitals of **5L^Re,Au^**. (**a**) The HOMO (−6.09 eV); (**b**) the LUMO (−2.66 eV).

DFT was also used to examine the electronic structure of the ligands and complexes. In the free ligands, the HOMO is mainly centred on the phosphine, with the LUMO dominated by the π* orbital on the bpy. In the Re complexes, the HOMO is metal-centred with small contributions from CO ligands, while the LUMO is still predominantly of bpy π* character. This is not significantly altered by the addition of AuCl on P. A gold-based HOMO is observed upon coordination of AuCl to P but the LUMO remains as bpy π*. Representative plots are shown in Figure 5, and frontier orbital energies are summarised in the Supplementary Materials.

## 3. Materials and Methods

All chemicals were purchased from commercial sources and used without further purification unless otherwise stated. All reactions and manipulations involving phosphines were performed under nitrogen using standard Schlenk techniques and previously dried, degassed solvents. NMR spectra were recorded on Bruker Fourier 300, DPX 400, and Avance 500 or 600 MHz NMR spectrometers. ^1^H and ^13^C{^1^H} NMR chemical shifts were referenced relative to the residual solvent resonances in the deuterated solvent. Mass spectra (ESI) were recorded on a Waters LCT premier XE spectrometer. UV/Vis spectra were obtained on a Cary 60 spectrophotometer and recorded over the range of 800 to 250 nm, with a 600 nm min^−1^ scan rate using a 1 cm path length quartz cuvette. Emission spectra were collected using a Cary Eclipse spectrophotometer from 700 to 450 nm, with an excitation wavelength of 410 nm and a 600 nm min^−1^ scan rate.

Single-crystal XRD data were collected using Mo-Kα radiation of 0.71073 Å on an Agilent SuperNova Dual Atlas diffractometer with a mirror monochromator. The sample temperature was maintained at 200K using an Oxford Cryosystems cooling apparatus. The crystal structures were solved using SHELXT [48] and refined using SHELXL [49]. Non-hydrogen atoms were refined with anisotropic displacement parameters. Hydrogen atoms were inserted in idealized positions, and a riding model was used with their Uiso set at 1.2 or 1.5 times the value of Ueq for the atom to which they are bonded. In the structure of **5L^Re,Rh^**, the chloro and carbonyl ligands coordinated to Re are disordered, with refined Cl/CO occupancies of 0.784(7)/0.216(7) and 0.216(7)/0.784(7) over two sites. CCDC 2327744 and 2327745 contain the supplementary crystallographic data for this paper. These data can be obtained free of charge via http://www.ccdc.cam.ac.uk/conts/retrieving.html (accessed on 23 January 2024) or from the CCDC, 12 Union Road, Cambridge CB2 1EZ, UK; Fax: +44-1223-336033; E-mail: deposit@ccdc.cam.ac.uk. A table of pertinent details of the data collection and refinement is included in the ESI.

DFT calculations were carried out using the Gaussian09 program [50]. Rotational energy profiles and subsequent optimisations were calculated at the BP86/def2SVP level [51,52,53] using density fitting with automated fitting basis set assignment and D3 empirical dispersion correction [54]. All minima were confirmed as such by harmonic frequency calculation. The subsequent calculation of orbital energies used the B3LYP/def2TZVP level [55].

### 3.1. Synthesis of CgP-5bpy (***5L***)

A mixture of 1,3,5,7-tetramethyl-2,4,6-Trioxa-8-phosphatricyclo[3.3.1.13,7]decane (1.6 g, 7.4 mmol), 5-bromobpyridine (1.73 g, 7.4 mmol), Pd_2_(dba)_3_ (0.1 g, 0.12 mmol), BINAP (0.3 g, 0.48 mmol), and NaO^t^Bu (2.0 g, 20.1 mmol) in toluene (100 mL) was heated at 100 °C for 48 h under nitrogen. After cooling to RT, the toluene was reduced to half volume in vacuo and 6 M HCl (40 mL) was added thereto. The biphasic mixture was stirred vigorously for 20 min and allowed to settle, and the organic phase was removed via a cannula. Et_2_O (50 mL) was added to the aqueous phase and the process was repeated. The aqueous phase was basified with solid NaOH with ice cooling and subsequently extracted with Et_2_O (2 × 50 mL). Each organic phase was isolated by cannula, dried over MgSO_4_, filtered, and taken to dryness at the pump. The sticky orange residue was triturated with 40/60 petroleum ether to remove some insoluble residues. Isolation and removal of the petroleum ether gave a pale yellow solid. Yield = 2.23 g (77%). ^1^H (CDCl_3_, 400 MHz): δ_H_ 8.94 (m, 1H), 8.63 (d, ^3^*J*_HP_ 4.8 Hz, 1H), 8.33 (t, ^3^*J*_HH_ 8.1 Hz, 2H), 8.24 (m, 1H), 7.77 (td, ^3^*J*_HH_ ^4^*J*_HH_ 7.7, 1.6 Hz, 1H), 7.26 (m, 1H), 2.20-1.85 (m, 3H), 1.65 (d, ^3^*J*_HP_ 13.5 Hz, 1H), 1.47 (d, ^3^*J*_HP_ 13.8 Hz, 3H), 1.37 (s, 6H), 1.22 (d, ^3^*J*_HP_ 13.4 Hz, 3H) ppm. ^13^C{^1^H} (CDCl_3_, 100 MHz): δ_C_ 156.7 (C), 155.7 (C), 155.0 (CH), 154.7 (CH), 149.4 (CH), 143.2 (d, ^2^*J*_CP_ 15.1 Hz, CH), 130.5 (d, ^1^*J*_CP_ 36.1 Hz, C), 124.1 (CH), 121.3 (CH), 120.6 (d, ^3^*J*_CP_ 4.7 Hz, CH), 96.9 (C), 96.1 (C), 73.3 (d, ^1^*J*_CP_ 22.2 Hz, C), 73.2 (d, ^1^*J*_CP_ 7.1 Hz, C), 45.2 (d, ^2^*J*_CP_ 18.2 Hz, CH_2_), 36.4 (d, ^2^*J*_CP_ 1.6 Hz, CH_2_), 28.0 (CH_3_), 27.8 (CH_3_), 27.4 (d, ^2^*J*_CP_ 22.2 Hz, CH_3_), 26.8 (d, ^2^*J*_CP_ 11.1 Hz, CH_3_) ppm. ^31^P{^1^H} (CDCl_3_, 162 MHz): δ_P_ −30.5 ppm. HRMS (ES): *m*/*z* 371.1528 (calc. 371.1525) [L]^+^, 100%.

### 3.2. Synthesis of CgP-6bpy (***6L***)

Prepared as for the **5L** derivative above. Yield = 2.35 g (81%). ^1^H (CDCl_3_, 400 MHz): δ_H_ 8.61 (ddd, ^3^*J*_HH_ ^4^*J*_HH_ 4.9, 1.9, 0.9 Hz, 1H), 8.43 (dt, ^3^*J*_HH_ ^4^*J*_HH_ 8.0, 1.1 Hz, 1H), 8.29 (dt, ^3^*J*_HH_ ^4^*J*_HH_ 7.9, 1.1 Hz, 2H), 7.85 (dd, ^3^*J*_HH_ ^4^*J*_HH_ 7.7, 1.0 Hz, 1H), 7.76 (td, ^3^*J*_HH_ ^4^*J*_HH_ 7.7, 1.8 Hz, 1H), 7.70 (td, ^3^*J*_HH_ ^4^*J*_HP_ 7.9, 1.7 Hz, 1H), 7.25 (ddd, ^3^*J*_HH_ ^3^*J*_HH_ ^4^*J*_HH_ 7.5, 4.7, 1.3 Hz, 1H), 2.05 (dd, ^2^*J*_HH_ ^3^*J*_HP_ 13.2, 6.6 Hz, 1H), 1.88 (dd, ^3^*J*_HP_ ^2^*J*_HH_ 23.5, 13.2 Hz, 1H), 1.82 (d, ^2^*J*_HH_ 13.2 Hz, 1H), 1.56 (d, ^3^*J*_HP_ 12.4 Hz, 3H), 1.48 (dd, ^2^*J*_HH_ ^3^*J*_HP_ 13.3, 4.2 Hz, 1H), 1.43 (d, ^3^*J*_HP_ 12.5 Hz, 3H), 1.37 (s, 6H), 1.31 (s, 3H) ppm. ^13^C{^1^H} (CDCl_3_, 100 MHz): δ_C_ 160.0 (d, ^1^*J*_CP_ 13.4 Hz, C), 156.2 (d, ^3^*J*_CP_ 12.0 Hz, C), 155.8 (C), 149.1 (CH), 137.0 (CH), 136.3 (CH), 129.2 (d, ^2^*J*_CP_ 11.7 Hz, CH), 123.9 (CH), 121.2 (CH), 120.0 (CH), 96.8 (C), 96.2 (C), 73.5 (d, ^1^*J*_CP_ 9.6 Hz, C), 72.9 (d, ^1^*J*_CP_ 22.7 Hz, C), 45.1 (d, ^2^*J*_CP_ 16.7 Hz, CH_2_), 37.5 (d, ^2^*J*_CP_ 1.9 Hz, CH_2_), 28.0 (CH_3_), 27.8 (CH_3_), 27.7 (d, ^2^*J*_CP_ 23.0 Hz, CH_3_), 27.3 (d, ^2^*J*_CP_ 11.7 Hz, CH_3_) ppm. ^31^P{^1^H} (CDCl_3_, 162 MHz): δ_P_ −24.9 ppm. HRMS (ES): *m*/*z* 371.1521 (calc. 371.1525) [L]^+^, 40%.

### 3.3. fac-[Re(κ^2^-N,N′-***5L***)(CO)_3_Cl], ***5L^Re^***

A mixture of **5L** (400 mg, 1.08 mmol) and Re(CO)_5_Cl (390 mg, 1.08 mmol) was heated at ~100 °C in degassed chlorobenzene (20 mL) for 6 h under nitrogen. After cooling, the volatiles were removed in vacuo to give an orange solid which was triturated with O_2_-free diethyl ether (10 mL) to give an insoluble and soluble fraction. The insoluble fraction was determined to be >90% pure by NMR spectroscopy and therefore used for the subsequent chemistry, whereas the soluble fraction proved to be less pure and was discarded. Yield = 526 mg (72%). ^1^H (*d*_6_-acetone, 400 MHz): δ_H_ 9.47 (m, 0.5H), 9.41 (m, 0.5H), 8.99 (d, 5.1 Hz, 1H), 8.56 (m, 1H), 8.42 (m, 1H), 8.21 (m, 1H), 7.67 (m, 1H), 7.26 (m, 1H), 1.55–1.35 (m, 5H), 1.31 (s, 1.5H), 1.29 (s, 1.5H), 1.23 (s, 1.5H), 1.23 (s, 1.5H), 1.18 (d, ^3^*J*_HP_ 8.6 Hz, 1.5H), 1.15 (d, ^3^*J*_HP_ 8.6 Hz, 1.5H) ppm. ^13^C{^1^H} (C_6_D_6_, 150 MHz): δ_C_ 197.4 (2 × CO), 196.7 (CO) 196.5 (CO), 189.2 (2 × CO), 156.3 (C), 156.2 (C), 156.1 (C), 156.0 (C), 154.4 (CH), 154.2 (CH), 154.0 (CH), 153.9 (CH), 151.8 (2 × CH), 143.9 (d, ^2^*J*_CP_ 29.7 Hz, CH), 143.5 (d, ^2^*J*_CP_ 23.0 Hz, CH), 135.4 (d, ^1^*J*_CP_ 40.0 Hz, C), 135.1 (d, ^1^*J*_CP_ 39.7 Hz, C), 128.7 (CH), 127.6 (CH), 121.9 (CH), 121.7 (CH), 121.2 (d, ^3^*J*_CP_ 6.8 Hz, CH), 120.8 (d, ^3^*J*_CP_ 8.8 Hz, CH), 96.0 (C), 95.9 (C), 95.7 (C), 95.2 (C), 72.6 (d, ^1^*J*_CP_ 7.8 Hz, C), 72.4 (d, ^1^*J*_CP_ 7.7 Hz, C), 72.2 (d, ^1^*J*_CP_ 22.2 Hz, C), 72.0 (d, ^1^*J*_CP_ 21.9 Hz, C), 43.6 (d, ^2^*J*_CP_ 17.3 Hz, CH_2_), 43.5 (d, ^2^*J*_CP_ 17.4 Hz, CH_2_), 35.4 (CH_2_), 35.3 (CH_2_), 26.8 (CH_3_), 26.7 (CH_3_), 26.6 (CH_3_), 26.5 (CH_3_), 26.3 (d, ^2^*J*_CP_ 22.0 Hz, CH_3_), 26.2 (d, ^2^*J*_CP_ 22.1 Hz, CH_3_), 25.7 (CH_3_), 25.6 (CH_3_) ppm. ^31^P{^1^H} (CDCl3, 162 MHz): δ_P_ −31.5, −31.6 ppm. HRMS (ES): *m*/*z* 641.0850 (calc. 641.0851) [M − Cl]^+^, 100%. IR ν(CO): 2016, 1913, 1884, 1867 cm^−1^. UV/Vis: λ_max_(CH_2_Cl_2_)/nm 263 (ε/dm^3^ mol^−1^ cm^−1^ 65,288), and 303 (24,464).

### 3.4. fac-[Re(κ^2^-N,N′-***6L***)(CO)_3_Cl], ***6L^Re^***

Prepared as detailed in *3.3*. Yield = 599 mg (82%). ^1^H (*d*_6_-dmso, 400 MHz, major isomer): δ_H_ 9.10 (dd, ^3^*J*_HH_ ^4^*J*_HH_ 5.5, 0.7 Hz, 1H), 8.80 (d, ^3^*J*_HH_ 8.2 Hz, 1H), 8.72 (d, ^3^*J*_HH_ 8.3 Hz, 1H), 8.52 (d, ^3^*J*_HH_ 7.7 Hz, 1H), 8.35 (m, 2H), 7.76 (m, 1H), 2.06 (m, 1H), 2.02 (d, ^3^*J*_HP_ 10.3 Hz, 1H), 1.82 (d, ^2^*J*_HH_ 13.9 Hz, 1H), 1.59 (dd, ^2^*J*_HH_ ^3^*J*_HP_ 14.0, 4.1 Hz, 1H), 1.45 (s, 3H), 1.44 (d, ^3^*J*_HP_ 12.6 Hz, 3H), 1.37 (s, 3H), 1.27 (d, ^3^*J*_HP_ 12.6 Hz, 3H) ppm. ^13^C{^1^H} (*d*_6_-dmso, 100 MHz, isomeric mixture): δ_C_ 198.4 (CO), 195.6 (CO) 191.9 (CO), 163.6 (C), 163.3 (C), 159.1 (d, ^1^*J*_CP_ 7.7 Hz, C), 156.9 (C), 156.4 (C), 153.2 (CH), 153.1 (CH), 140.7 (CH), 140.6 (CH), 139.9 (CH), 133.2 (CH), 132.7 (CH), 130.8 (C), 128.9 (C), 128.3 (CH), 125.1 (d, ^2^*J*_CP_ 11.0 Hz, CH), 125.2 (CH), 97.3 (C), 97.2 (C), 96.2 (C), 75.1 (d, ^1^*J*_CP_ 13.3 Hz, C), 74.7 (d, ^1^*J*_CP_ 12.5 Hz, C), 74.5 (d, ^1^*J*_CP_ 27.2 Hz, C), 74.1 (d, ^1^*J*_CP_ 27.0 Hz, C), 45.0 (d, ^2^*J*_CP_ 19.3 Hz, CH_2_), 35.9 (d, ^2^*J*_CP_ 36.8 Hz, CH_2_), 28.8 (d, ^2^*J*_CP_ 17.9 Hz, CH_3_), 28.5 (d, ^2^*J*_CP_ 21.0 Hz, CH_3_), 27.8 (CH_3_), 27.6 (CH_3_), 26.8 (d, ^2^*J*_CP_ 10.3 Hz, CH_3_), 26.7 (d, ^2^*J*_CP_ 10.2 Hz, CH_3_) ppm. ^31^P{^1^H} (CDCl_3_, 162 MHz): δ_P_ −15.9, −18.1 ppm. HRMS (ES): *m*/*z* 639.0819 (calc. 639.0823) [M − Cl]^+^, 100%. IR ν(CO): 2010, 1920sh, 1896, 1854 cm^−1^. UV/Vis: λ_max_(CH_2_Cl_2_)/nm 260 (ε/dm^3^ mol^−1^ cm^−1^ 15,610), and 307 (17,153).

### 3.5. fac-[Re(κ^2^-N,N′-Re,κ-P-Au-***5L***)(CO)_3_Cl(AuCl)], ***5L^Re,Au^***

Au(THT)Cl (47 mg, 1.48 × 10^−4^ mol) was added to a solution of *fac*-[Re(κ^2^-*N,N*′-**5L**)(CO)_3_Cl] (100 mg, 1.48 × 10^−4^ mol) in CH_2_Cl_2_ (10 mL). After stirring for 1 h, the volatiles were removed in vacuo and the resultant orange solid crystallised from acetone by the slow inclusion of pentane. Yield = 103 mg (77%). The initial NMR spectra showed the presence of two isomers which slowly became enriched to a major and minor over several weeks in solution (*d*_6_-acetone). After slow evaporation of the *d*_6_-acetone, the yellow solid was dissolved in CDCl_3_, which then showed the presence of a single isomer. ^1^H (*d*_6_-acetone, 500 MHz): δ_H_ 9.67 (m, 2H), 9.04 (m, 2H), 8.79 (m, 4H), 8.69 (m, 2H), 8.26 (tt, 7.9, 1.6 Hz, 2H), 7.73 (m, 2H), 2.55 (dd, ^3^*J*_HP_ 6.5, 5.9 Hz, 1H), 2.53 (dd, ^3^*J*_HP_ 6.4, 5.9 Hz, 1H), 2.10–1.95 (m, 5H), 1.77 (dd, ^2^*J*_HH_ ^3^*J*_HP_ 14.4, 1.8 Hz, 1H), 1.68 (d, ^3^*J*_HP_ 15.5 Hz, 3H), 1.61 (d, ^3^*J*_HP_ 15.4 Hz, 3H), 1.44 (s, 3H), 1.43 (s, 3H), 1.37 (s, 3H), 1.36 (s, 3H), 1.35 (d, ^3^*J*_HP_ 16.4 Hz, 3H), 1.33 (d, ^3^*J*_HP_ 16.6 Hz, 3H) ppm. ^13^C{^1^H} (d_6_-acetone, 150 MHz): δ_C_ 197.8 (CO), 197.4 (CO), 189.2 (CO), 158.7 (C, d, ^1^*J*_CP_ 21.6 Hz), 156.2 (CH, d, ^2^*J*_CP_ 10.8 Hz), 156.1 (CH, d, ^2^*J*_CP_ 8.4 Hz), 154.7 (C), 153.5 (CH), 147.7 (CH, d, ^2^*J*_CP_ 15.0 Hz), 147.5 (CH, d, ^2^*J*_CP_ 15.5 Hz), 140.2 (CH), 128.7 (CH), 128.6 (CH), 126.4 (C, d, ^1^*J*_CP_ 38.1 Hz), 125.3 (CH), 125.2 (CH), 124.3 (CH, d, ^3^*J*_CP_ 8.1 Hz), 124.2 (CH, ^3^*J*_CP_ d, 7.9 Hz), 97.0 (2 × C, d, ^1^*J*_CP_ 81.6, 44.6 Hz), 74.5 (C, d, ^1^*J*_CP_ 29.4 Hz), 74.0 (C, d, ^1^*J*_CP_ 35.2 Hz), 43.6 (CH_2_, d, ^2^*J*_CP_ 10.1 Hz), 36.5 (CH_2_), 26.8 (CH_3_, d, ^2^*J*_CP_ 16.7 Hz), 26.6 (CH_3_, d, ^2^*J*_CP_ 15.2 Hz), 26.0 (CH_3_, d, ^2^*J*_CP_ 8.0 Hz), 25.9 (CH_3_, d, ^2^*J*_CP_ 7.3 Hz), 24.9 (CH_3_, d, ^2^*J*_CP_ 4.3 Hz), 24.8 (CH_3_, d, ^2^*J*_CP_ 4.0 Hz) ppm. ^31^P{^1^H} (d_6_-acetone, 202 MHz): δ_P_ 20.9, 20.5 ppm. HRMS (ES): *m*/*z* 873.0209 (calc. 873.0206) [M − Cl]^+^, 35%. IR ν(CO): 2016, 1917, 1890, 1875 cm^−1^. UV/Vis: λ_max_(CH_2_Cl_2_)/nm 261 (ε/dm^3^ mol^−1^ cm^−1^ 9441), and 310 (22,631).

### 3.6. fac-[Re(κ^2^-N,N′-Re,κ-P-Au-***6L***)(CO)_3_Cl(AuCl)], ***6L^Re,Au^***

Au(THT)Cl (20 mg, 0.63 × 10^−4^ mol) was added to a suspension of *fac*-[Re(κ^2^-*N,N*′-**6L**)(CO)_3_Cl] (44 mg, 0.65 × 10^−4^ mol) in CH_2_Cl_2_ (8 mL) and the suspension was stirred overnight. On return, a solid and yellow solution was obtained. Dmso (0.5 mL) was added to ensure complete dissolution and obtained ^31^P{^1^H} NMR—one species only at δ_P_ 22.8 ppm. The CH_2_Cl_2_ was removed in vacuo and the resultant dmso solution was diluted with water (3 mL) to give a yellow precipitate which was filtered and air-dried. Yield = 48%. Alternatively, *fac*-[Re(κ^2^-*N,N*′-**6L**)(CO)_3_Cl] (50 mg, 0.74 × 10^−4^ mol) was added to CH_2_Cl_2_ (30 mL) to give a slight suspension, whereupon one equiv. of Au(THT)Cl (23 mg) was added. Within a few minutes, the mixture clarified to an orange solution. After stirring for 10 min, the volume was reduced and the complex precipitated by the addition of diethyl ether and then filtered and air-dried. Yield = 79%. ^1^H (d_6_-dmso, 400 MHz): δ_H_ 8.63 (dt, ^3^*J*_HH_ ^4^*J*_HH_ 4.7, 0.9 Hz, 1H), 8.51 (dd, ^3^*J*_HH_ ^4^*J*_HH_ 8.1, 2.8 Hz, 1H), 8.38 (d, ^3^*J*_HH_ 7.9 Hz, 1H), 8.05 (m, 1H), 7.87 (td, ^3^*J*_HH_ ^3^*J*_HP_ ^3^*J*_HH_ 7.8, 3.9 Hz, 1H), 7.80 (td, ^3^*J*_HH_ ^3^*J*_HH_ ^4^*J*_HH_ 7.8, 1.7 Hz, 1H), 7.31 (dd, ^3^*J*_HH_ ^3^*J*_HH_ 7.4, 4.8 Hz, 1H), 2.56 (dd, ^2^*J*_HH_ ^3^*J*_HP_ 13.8, 5.0 Hz, 1H), 2.33 (d, ^2^*J*_HH_ 13.4 Hz, 1H), 1.93 (dd, ^2^*J*_HH_ ^3^*J*_HP_ 13.6, 7.6 Hz, 1H), 1.88 (d, ^2^*J*_HH_ 12.8 Hz, 1H), 1.54 (d, ^3^*J*_HP_ 15.0 Hz, 3H), 1.44 (d, ^3^*J*_HP_ 16.0 Hz, 3H), 1.42 (s, 3H), 1.40 (s, 3H) ppm. ^13^C{^1^H} (d_6_-dmso, 100 MHz): δ_C_ 196.8 (CO), 193.1 (CO), 187.7 (CO), 157.0 (C, d, ^1^*J*_CP_ 16.3 Hz), 154.5 (C), 150.1 (CH), 149.6 (C), 139.2 (CH, d, ^3^*J*_CP_ 8.1 Hz), 138.2 (CH), 132.2 (CH, d, ^2^*J*_CP_ 23.0 Hz), 138.1 (CH), 132.2 (CH, d, ^2^*J*_CP_ 23.0 Hz), 125.5 (CH), 123.4 (CH, d, ^2^*J*_CP_ 2.1 Hz), 121.0 (CH), 97.3 (C), 96.8 (C, d, ^3^*J*_CP_ 1.6 Hz), 74.1 (C, d, ^1^*J*_CP_ 5.6 Hz), 73.8 (C, d, ^1^*J*_CP_ 11.6 Hz), 43.9 (CH_2_, d, ^2^*J*_CP_ 9.4 Hz), 37.6 (CH_2_), 27.5 (CH_3_), 27.4 (CH_3_), 27.1 (CH_3_, d, ^2^*J*_CP_ 6.7 Hz), 25.7 (CH_3_, d, ^2^*J*_CP_ 4.5 Hz) ppm. ^31^P{^1^H} (CDCl_3_, 162 MHz): δ_P_ 22.5 ppm. HRMS (ES): *m*/*z* 873.0197 (calc. 873.0206) [M − Cl]^+^, 30%; 864.0553 (calc. 864.0548) [M − 2Cl + CN]+, 100%. IR: 2010, 1922sh, 1886 cm^−1^. UV/Vis: λ_max_(CH_2_Cl_2_)/nm 260 (ε/dm^3^ mol^−1^ cm^−1^ 13,870), and 312 (19,668).

### 3.7. fac-[{Re(κ^2^-N,N′-Re,κ-P-Ag-***5L***)(CO)_3_Cl}_2_(Ag)]OTf, ***5L^Re,Ag^***

Ag(OTf) (19 mg, 0.74 × 10^−4^ mol) was added to a solution of *fac*-[Re(κ^2^-*N,N*′-**5L**)(CO)_3_Cl] (100 mg, 1.48 × 10^−4^ mol) in CH_2_Cl_2_ (10 mL). After stirring for 1 h, the volatiles were removed in vacuo and the resultant orange solid crystallised from acetone by the slow inclusion of pentane. Yield = 56%. ^1^H (d_6_-dmso, 500 MHz): δ_H_ 9.58 (m, 2H), 9.12 (d, ^3^*J*_HH_ 5.3 Hz, 2H), 8.94 (t, 8.3 Hz, 2H), 8.88 (t, 8.3 Hz, 2H), 8.80 (t, 8.1 Hz, 2H), 8.44 (qd, 8.0, 1.4 Hz, 2H), 7.87 (qd, 8.1, 1.4 Hz, 2H), 2.41 (m, 2H), 2.16 (d, ^2^*J*_HH_ 13.5 Hz, 1H), 2.10 (d, ^2^*J*_HH_ 14.0 Hz, 1H), 1.90-1.60 (m, 2H), 1.75 (d, ^3^*J*_HP_ 14.1 Hz, 6H), 1.49 (s, 3H), 1.48 (s, 3H), 1.45–1.36 (6H) ppm. ^13^C{^1^H} (d_6_-dmso, 150 MHz): δ_C_ 198.1 (CO), 197.8 (CO), 197.5 (CO), 196.7 (CO), 190.2 (CO), 190.1 (CO), 157.0 (C), 156.1 (CH, d, ^3^*J*_CP_ 7.2 Hz), 155.1 (C), 154.9 (C), 154.8 (C), 154.3 (CH, d, ^2^*J*_CP_ 5.0 Hz), 153.7 CH), 147.4 (CH, d, ^2^*J*_CP_ 24.8 Hz), 140.9 (CH), 129.0 (CH), 129.0 (CH), 125.6 (CH), 125.5 (CH), 124.6 (CH, d, ^3^*J*_CP_ 8.9 Hz), 97.0 (2 × C, d, ^1^*J*_CP_ 81.6, 44.6 Hz), 74.5 (C, d, ^1^*J*_CP_ 29.4 Hz), 74.0 (C, d, ^1^*J*_CP_ 35.2 Hz), 43.6 (CH_2_, d, ^2^*J*_CP_ 10.1 Hz), 36.5 (CH_2_), 26.8 (CH_3_, d, ^2^*J*_CP_ 16.7 Hz), 26.6 (CH_3_, d, ^2^*J*_CP_ 15.2 Hz), 26.0 (CH_3_, d, ^2^*J*_CP_ 8.0 Hz), 25.9 (CH_3_, d, ^2^*J*_CP_ 7.3 Hz), 24.9 (CH_3_, d, ^2^*J*_CP_ 4.3 Hz), 24.8 (CH_3_, d, ^2^*J*_CP_ 4.0 Hz) ppm. ^31^P{^1^H} (*d*_6_-dmso, 202 MHz): δ_P_ −16.0(br), −15.0(sh) ppm. LRMS (ES): *m*/*z* 1459.01 (calc. 1459.01) [M]^+^, 2%. IR ν(CO): 2019, 1886 cm^−1^. UV/Vis: λ_max_(CH_2_Cl_2_)/nm 259 (ε/dm^3^ mol^−1^ cm^−1^ 35,745), and 304 (41,689).

### 3.8. [Au(κ-P-***6L***)Cl]

The complex was prepared in situ by the addition of one equivalent of **6L** to one of [Au(THT)Cl] in CH_2_Cl_2_ at RT. The removal of all volatiles gave a slightly grey solid. Yield = quant. The complex was crystallised by the slow evaporation of an acetone solution. ^1^H (CDCl_3_, 400 MHz): δ_H_ 8.64 (ddd, ^3^*J*_HH_ ^4^*J*_HH_ 4.9, 1.7, 0.9 Hz, 1H), 8.54 (ddd, ^3^*J*_HH_ ^4^*J*_HH_ 8.0, 2.7, 0.7 Hz, 1H), 8.40 (dt, ^3^*J*_HH_ ^4^*J*_HH_ 8.0, 0.9 Hz, 1H), 8.06 (ddd, ^3^*J*_HH_ ^4^*J*_HH_ 7.7, 5.2, 1.0 Hz, 1H), 7.88 (td, ^3^*J*_HH_ ^3^*J*_HP_ ^4^*J*_HH_ 8.0, 3.8 Hz, 1H), 7.83 (td, 7.7, 1.7 Hz, 1H), 7.33 (ddd, ^3^*J*_HH_ ^3^*J*_HH_ ^4^*J*_HH_ 7.5, 4.8, 1.1 Hz, 1H), 2.56 (dd, ^2^*J*_HH_ ^3^*J*_HP_ 13.7, 5.0 Hz, 1H), 2.31 (dd, ^2^*J*_HH_ ^3^*J*_HP_ 13.6, 1.3 Hz, 1H), 1.93 (dd, ^2^*J*_HH_ ^3^*J*_HP_ 13.7, 7.7 Hz, 1H), 1.88 (dd, ^2^*J*_HH_ ^3^*J*_HP_ 13.7, 1.2 Hz, 1H), 1.54 (d, ^3^*J*_HP_ 15.2 Hz, 3H), 1.44 (d, ^2^*J*_HH_ 15.9 Hz, 1H), 1.42 (s, 3H), 1.40 (s, 3H) ppm. ^13^C{^1^H} (CDCl_3_, 100 MHz): δ_C_ 157.2 (C, d, ^1^*J*_CP_ 16.1 Hz), 154.4 (C), 150.1 (C), 149.5 (C), 149.1 (CH), 137.7 (CH), 137.5 (d, ^3^*J*_CP_ 8.9 Hz, CH), 132.0 (CH), 124.8 (CH), 123.2 (CH, d, ^2^*J*_CP_ 2.7 Hz), 121.7 (CH), 97.1 (C), 96.6 (d, ^3^*J*_CP_ 1.6 Hz, C), 74.0 (C, d, ^1^*J*_CP_ 24.3 Hz), 73.6 (C, d, ^1^*J*_CP_ 30.0 Hz), 44.1 (CH_2_, d, ^2^*J*_CP_ 9.8 Hz), 37.8 (CH_2_), 27.6 (CH_3_), 27.4 (CH_3_), 26.9 (CH_3_, d, ^2^*J*_CP_ 7.1 Hz), 25.7 (CH_3_, d, ^2^*J*_CP_ 4.4 Hz) ppm. ^31^P{^1^H} (CDCl_3_, 162 MHz): δ_P_ 22.6 ppm. HRMS (ES): *m*/*z* 603.0887 (calc. 603.0879) [M + H]^+^, 100%.

### 3.9. [Ag(κ-P-***6L***)_2_]BF_4_

The complex was prepared in situ by the addition of two equivalents of **6L** to one of AgBF4 in CH_2_Cl_2_. The removal of all volatiles left a greyish solid. Yield = quant. The NMR data were broad for this complex (see Supplementary Materials).

### 3.10. fac-[Re(κ^2^-N,N′-Re,κ-P-Rh-***5L***)(CO)_3_Cl(Rh(COD)Cl)], ***5L^Re,Rh^***

Prepared as for the gold complex. Recrystallised from toluene. Yield = 80%. ^1^H (d_6_-acetone, 500 MHz): δ_H_ 9.76 (d, ^3^*J*_HP_ 5.0 Hz, 1H), 9.14 (d, ^3^*J*_HH_ 5.5 Hz, 1H), 8.82 (t, ^3^*J*_HH_ ^3^*J*_HP_ 6.6 Hz, 1H), 8.72 (d, ^3^*J*_HH_ 8.2 Hz, 1H), 8.65 (d, ^3^*J*_HH_ 8.4 Hz, 1H), 8.38 (td, ^3^*J*_HH_ ^4^*J*_HH_ 8.5, 1.6 Hz, 1H), 7.84 (ddd, ^3^*J*_HH_ ^3^*J*_HH_ ^4^*J*_HH_ 7.7, 5.5, 1.1 Hz, 1H), 5.50 (sbr, 1H), 5.41 (sbr, 1H), 4.89 (sbr, 1H), 4.74 (sbr, 1H), 3.03 (svbr, 1H), 2.55 (svbr, 1H), 2.45-2.00 (br, 5H), 1.87 (d, ^3^*J*_HP_ 11.9 Hz, 3H), 1.79 (d, ^2^*J*_HH_ 14.2 Hz, 1H), 1.65 (d, ^3^*J*_HP_ 11.6 Hz, 3H), 1.62 (d, ^3^*J*_HP_ 11.2 Hz, 3H), 1.48 (s, 3H), 1.43 (s, 3H), 1.33 (d, ^3^*J*_HP_ 23.0 Hz, 3H) ppm. ^13^C{^1^H} (CDCl3, 125 MHz, isomeric mixture): δ_C_ 197.4 (2 × CO), 197.3 (CO), 196.5 (CO), 189.0 (CO), 188.9 (CO), 159.1 (CH, d, ^2^*J*_CP_ 23.5 Hz), 155.8 (CH), 155.2 (C), 155.1 (C), 153.2 (CH), 153.1 (CH), 146.6 (CH, d, ^2^*J*_CP_ 19.0 Hz), 142.9 (CH), 139.3 (CH), 139.2 (CH), 134.2 (C, d, ^1^*J*_CP_ 21.3 Hz), 134.0 (C, d, ^1^*J*_CP_ 19.5 Hz), 127.5 (CH), 123.9 (CH), 123.8 (CH), 121.9 (CH), 105.5 (CH, m), 105.2 (CH, m), 104.1 (CH, m), 97.0 (C), 96.9 (C), 96.4 (C), 96.0 (C), 78.7 (CH, d, ^1^*J*_CRh_ 13.7 Hz), 76.0 (C, t, ^1^*J*_CP_ ^2^*J*_CRh_ 11.0 Hz), 74.6 (C, d, ^1^*J*_CP_ 19.6 Hz), 74.5 (C, d, ^1^*J*_CP_ 19.0 Hz), 73.1 (CH, d, ^1^*J*_CRh_ 13.0 Hz), 72.8 (CH, d, ^1^*J*_CRh_ 12.6 Hz), 69.7 (CH, d, ^1^*J*_CRh_ 12.0 Hz), 69.4 (CH, d, ^1^*J*_CRh_ 12.0 Hz), 44.7 (CH_2_, t, ^2^*J*_CP_ ^3^*J*_CRh_ 11.4 Hz), 40.1 (CH_2_, d, ^2^*J*_CP_ 19.7 Hz), 30.9 (CH_2_), 29.3 (CH_2_), 28.7 (CH_3_), 28.5 (CH_2_), 28.1 (CH_2_), 27.5 (CH_3_), 27.4 (CH_3_), 26.3 (CH_3_, t, ^2^*J*_CP_ ^3^*J*_CRh_ 8.1 Hz) ppm. ^31^P{^1^H} (CDCl_3_, 162 MHz): δ_P_ 6.9 (d, ^1^*J*_PRh_ 153.0 Hz) ppm. HRMS (ES): *m*/*z* 887.0535 (calc. 887.0534) [M − Cl]^+^, 75%. IR ν(CO): 2018, 1884 cm^−1^. UV/Vis: λ_max_(CH_2_Cl_2_)/nm 254 (ε/dm^3^ mol^−1^ cm^−1^ 18,211), and 306 (23,887).

### 3.11. [Rh(κ-P-***6L***)(COD)Cl)]

The [Rh(κ-*P*-**6L**)(COD)Cl)] was prepared simply by combining [Rh(COD)_2_Cl]_2_ (20 mg, 4.1 × 10^−5^ mol) and **6L** (30 mg, 8.2 × 10^−5^ mol) in CH_2_Cl_2_ (3 mL) and removing the solvent to give an orange solid. Yield = 98%. ^1^H (CDCl_3_, 400 MHz): δ_H_ 8.62 (dt, ^3^*J*_HH_ ^4^*J*_HH_ 4.8, 0.8 Hz, 1H), 8.45 (d, ^3^*J*_HH_ 7.7 Hz, 1H), 8.34 (dt, ^3^*J*_HH_ ^4^*J*_HH_ 8.1, 0.9 Hz, 1H), 7.92 (d, ^3^*J*_HH_ 7.7 Hz, 1H), 7.82 (td, ^3^*J*_HH_ ^4^*J*_HH_ 7.9, 1.5 Hz, 1H), 7.73 (td, ^3^*J*_HH_ ^3^*J*_HP_ ^4^*J*_HH_ 7.9, 2.8 Hz, 1H), 7.29 (dd, ^3^*J*_HH_ ^3^*J*_HH_ 7.5, 4.9 Hz, 1H), 3.23 (dd, ^2^*J*_HH_ ^3^*J*_HP_ 13.2, 4.5 Hz, 1H), 2.29 (m, 4H), 2.04 (d, ^3^*J*_HP_ 11.4 Hz, 3H), 1.85 (m, 5H), 1.63 (d, ^3^*J*_HP_ 13.4 Hz, 3H), 1.42 (m, 2H), 1.40 (s, 3H), 1.19 (s, 3H) ppm. ^31^P{^1^H} (CDCl_3_, 162 MHz): δ_P_ 8.5 (d, ^1^*J*_PRh_ 152.5 Hz) ppm. HRMS (ES): *m*/*z* 581.1443 (calc. 581.1440) [M − Cl]^+^, 75%.

### 3.12. Reaction of [Rh(κ-P-***6L***)(COD)Cl)] with [Re(CO)_5_Cl]

[Rh(κ-*P*-**6L**)(COD)Cl)] was dissolved in PhCl (5 mL) and 29 mg (1 equiv.) of [Re(CO)_5_Cl] was added thereto. The mixture was heated at ~80 for 2 h to give a deep-purple precipitate, which was isolated by filtration. Yield = 32 mg. Note that this does not form simply upon heating [Rh(κ-*P*-**6L**)(COD)Cl)] in PhCl. NMR spectroscopic data were recorded quickly in *d*_6_-dmso as appreciable changes in the ^31^P{^1^H} NMR spectrum occurred within a relatively short timeframe (hrs) to give an orange solution which proved to be a mixture. For NMR and other data, see Supplementary Materials.

## 4. Conclusions

Two novel phospha–bpy hybrid ligands have been prepared and selectively coordinated to [Re(CO)_3_Cl] through the bpy donor set. The subsequent binding of a second metal-containing fragment has been explored and steric limitations have been exposed for the 6-phospha ligand compared with the 5-phospha derivative, to the extent that only the small AuCl group could be accommodated by the rhenium complex of the former. Hence, only the more available 5-phospha group is able to act as a ‘hospitable host’ for the larger second metal fragments explored here. Rotational isomers are present for a number of the isolated species and their relative energies compared by DFT calculations. From the UV/Vis spectra and DFT calculations, there is no substantive evidence of electronic communication between the rhenium core and the second metal centre, with orbital coefficients being localised in the frontier orbitals. Efforts continue to further explore the chemistry and application of these and related complexes.

## Data Availability

Data are contained within the article and Supplementary Materials.

## Supplementary Materials

The following supporting information can be downloaded at https://www.mdpi.com/article/10.3390/molecules29051150/s1: Figures S1–S81: NMR and mass spectra of the compounds; Figure S82: electronic spectra of the complexes; Figure S83: normalized emission spectra of the complexes; Table S1: Crystal data and structure refinement for **5L^Re,Au^** and **5L^Re,Rh^**; Table S2: Frontier molecular orbital energies.
