# Supplementary material for: Welcoming Neighbour or Inhospitable Host? Selective Second Metal Binding in 5- and 6-Phospha-Substituted Bpy Ligands
Source: Molecules. 2024 Mar 5;29(5):1150. doi: 10.3390/molecules29051150 (PMC10933870; doi:10.3390/molecules29051150)
Supplement: Supplementary file 1 [file molecules-29-01150-s001.zip › molecules-2868087-supplementary.pdf]

Supplementary material.

Welcoming Neighbour or Inhospitable Host?: Selective Second Metal Binding in 5- and 6-Phospha Substituted Bipy Ligands

*Benson M. Kariuki, James A. Platts and Paul D. Newman**

School of Chemistry, Cardiff University, Cardiff, CF10 3AT, UK.

Contents

NMR and Mass spectra:

1. 5-bipyCgP (**5L**) S2
2. 6-bipyCgP (**6L**) S4
3. *Fac*-[Re(CO)$_3$Cl(κ^2-*N,N'*-**5L**)] (**5L^{Re}**) S9
4. *Fac*-[Re(CO)$_3$Cl(κ^2-*N,N'*-**6L**)] (**6L^{Re}**) S12
5. *Fac*-[Re(CO)$_3$Cl(κ^2-*N,N'*-Re,κ-*P*-Au-**5L**)(AuCl)] (**5L^{Re,Au}**) S15
6. *Fac*-[Re(CO)$_3$Cl(κ^2-*N,N'*-Re,κ-*P*-Au-**6L**)(AuCl)] (**6L^{Re,Au}**) S24
7. *Fac*-[{Re(CO)$_3$Cl(κ^2-*N,N'*-Re,κ-*P*-Ag-**5L**)}$_2$(Ag)]OTf (**5L^{Re,Ag}**) S29
8. [Au(κ-*P*-**6L**)Cl] S33
9. [Ag(κ-*P*-**6L**)$_2$]BF$_4$ S35
10. *Fac*-[Re(CO)$_3$Cl(κ^2-*N,N'*-Re,κ-*P*-Rh-**5L**)(Rh(COD)Cl)] (**5L^{Re,Rh}**) S36
11. [Rh(κ-*P*-**6L**)(COD)Cl] S41
12. Reaction of [Rh(κ-*P*-**6L**)(COD)Cl)] with [Re(CO)$_5$Cl]. S43

3. Electronic spectra S46

4. Emission spectra S47

5. Crystallographic data S47

6. Theoretical calculations S49

NMR and Mass Spectra

1. 5-bipyCgP (**5L**).

Figure S1. $^{31}P\{^{1}H\}$ NMR spectrum of **5L** recorded at 162 MHz in $CDCl_3$.

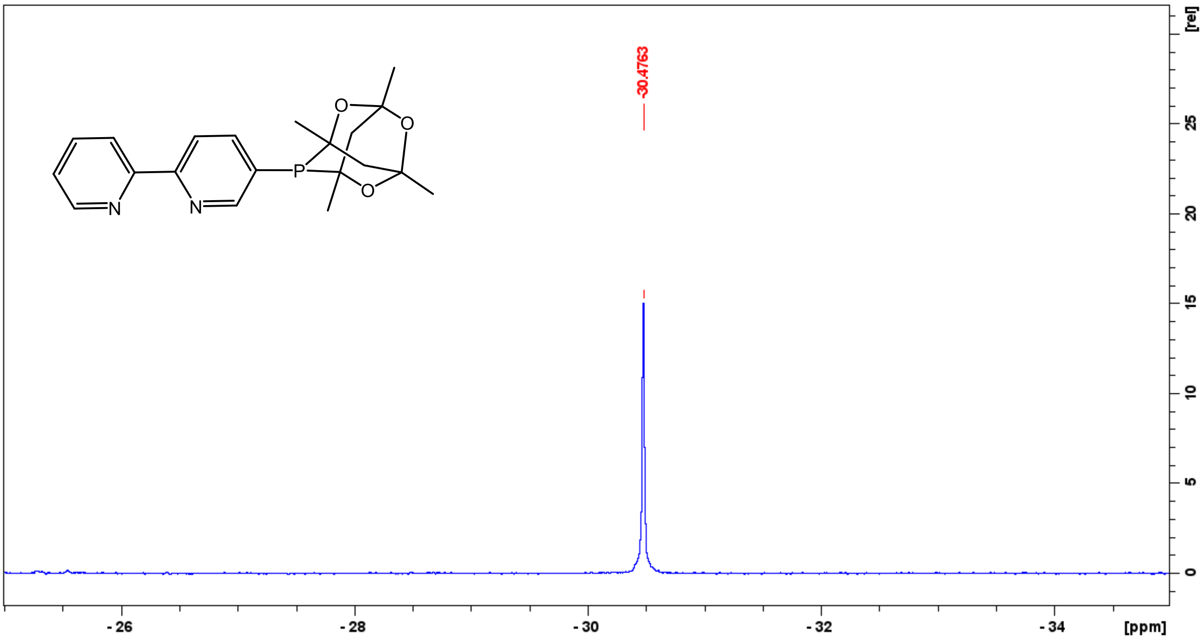

Figure S2. ^{1}H NMR spectrum of **5L** recorded at 500 MHz in $CDCl_3$.

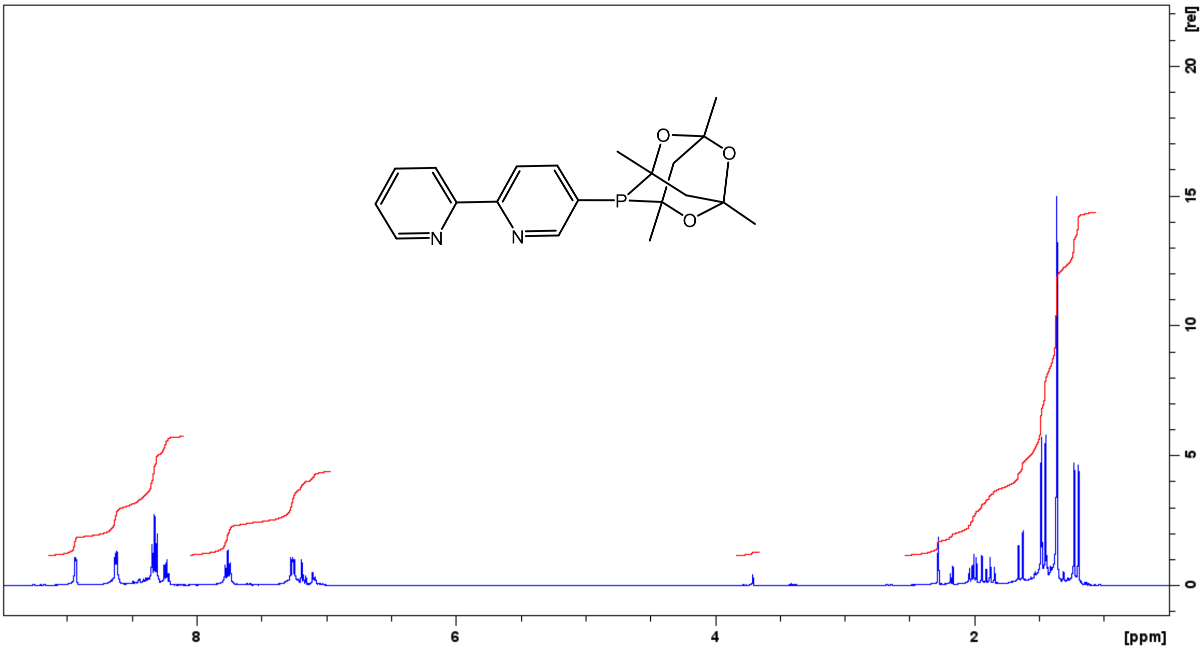

Figure S3. $^{13}C\{^{1}H\}$ NMR spectrum of **5L** recorded at 100 MHz in $CDCl_3$.

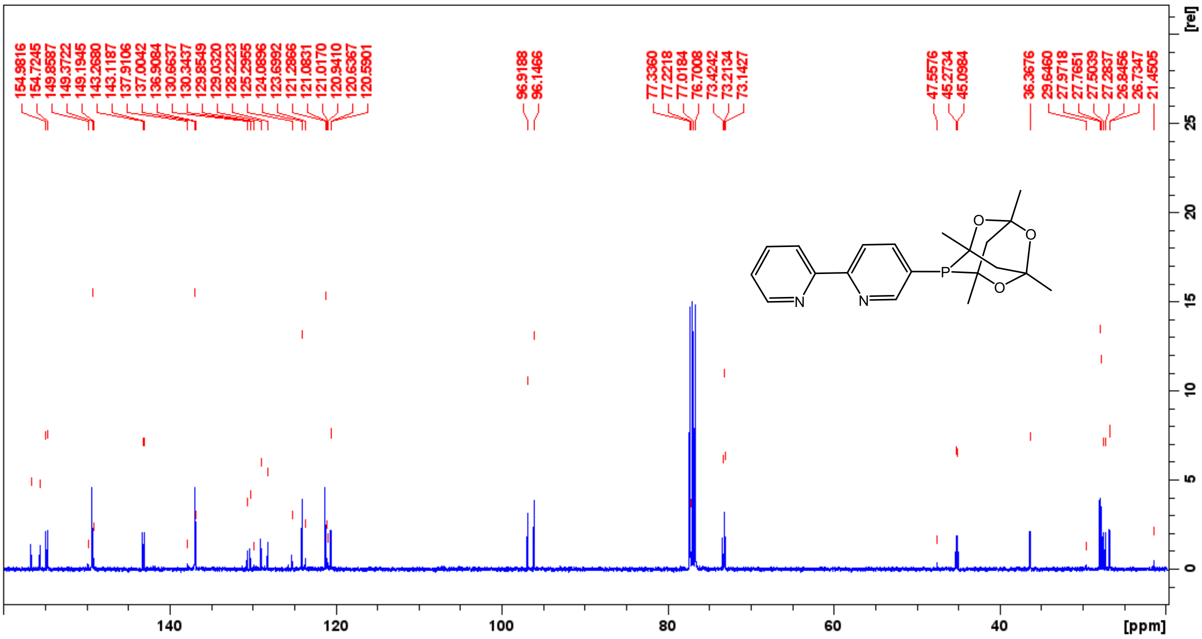

Figure S4. ^{1}H-^{13}C HSQC NMR spectrum of **5L** recorded in $CDCl_3$.

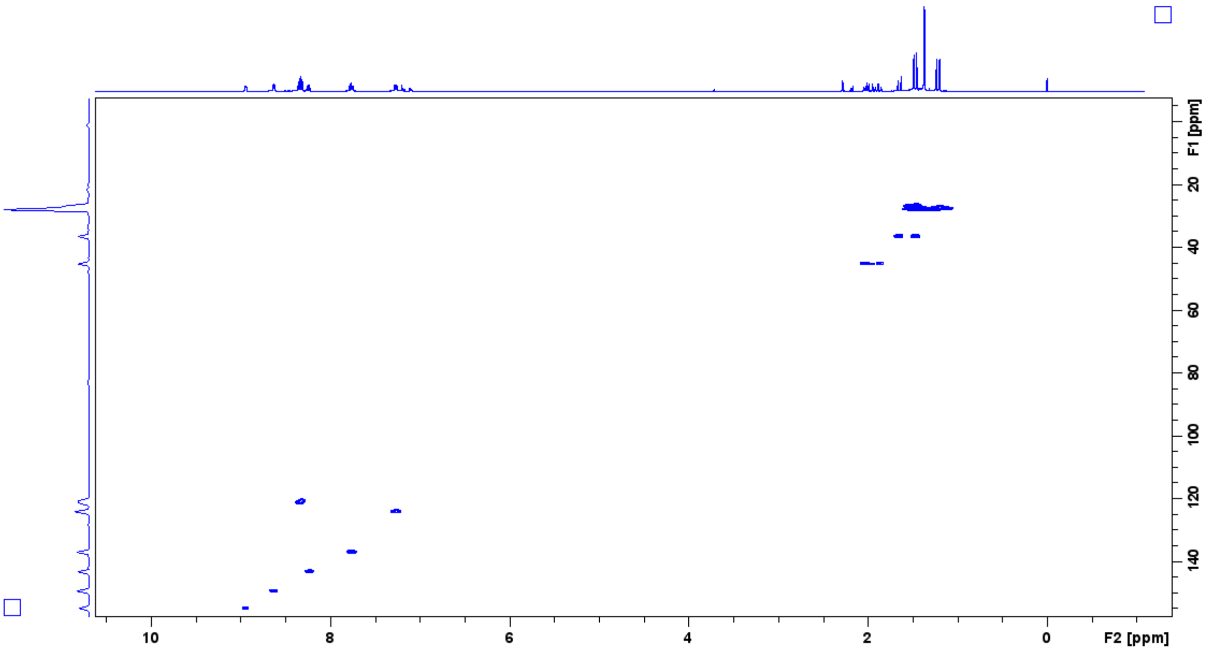

Figure S5. HRMS (ES^{+}) spectrum of **5L**.

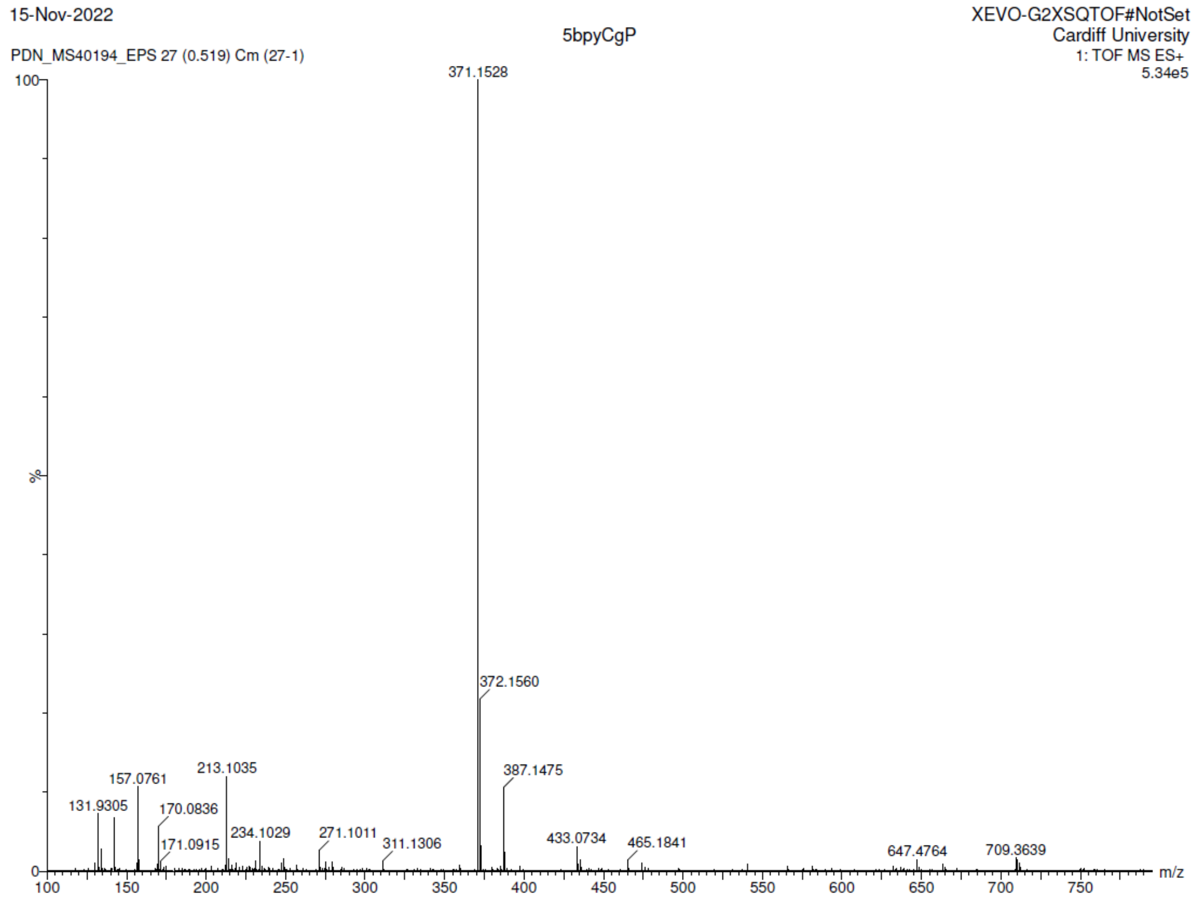

Minimum:				-1.5				
Maximum:		5.0	5.0	100.0				
Mass	Calc. Mass	mDa	PPM	DBE	i-FIT	Norm	Conf(%)	Formula
371.1528	371.1525	0.3	0.8	10.5	344.4	n/a	n/a	C20 H24 N2 O3 P

2. 6-bipyCgP (**6L**).

Figure S6. $^{31}P\{^1H\}$ NMR spectrum of **6L** recorded at 162 MHz in $CDCl_3$.

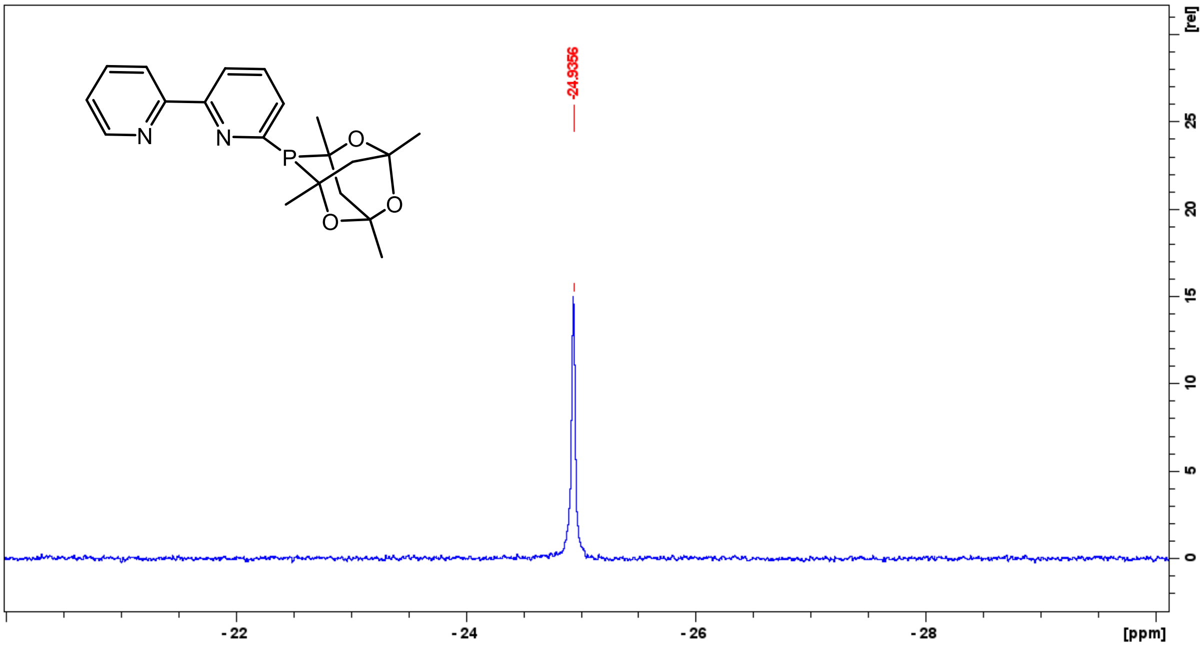

Figure S7. ^{1}H NMR spectrum of **6L** recorded at 400 MHz in $CDCl_3$.

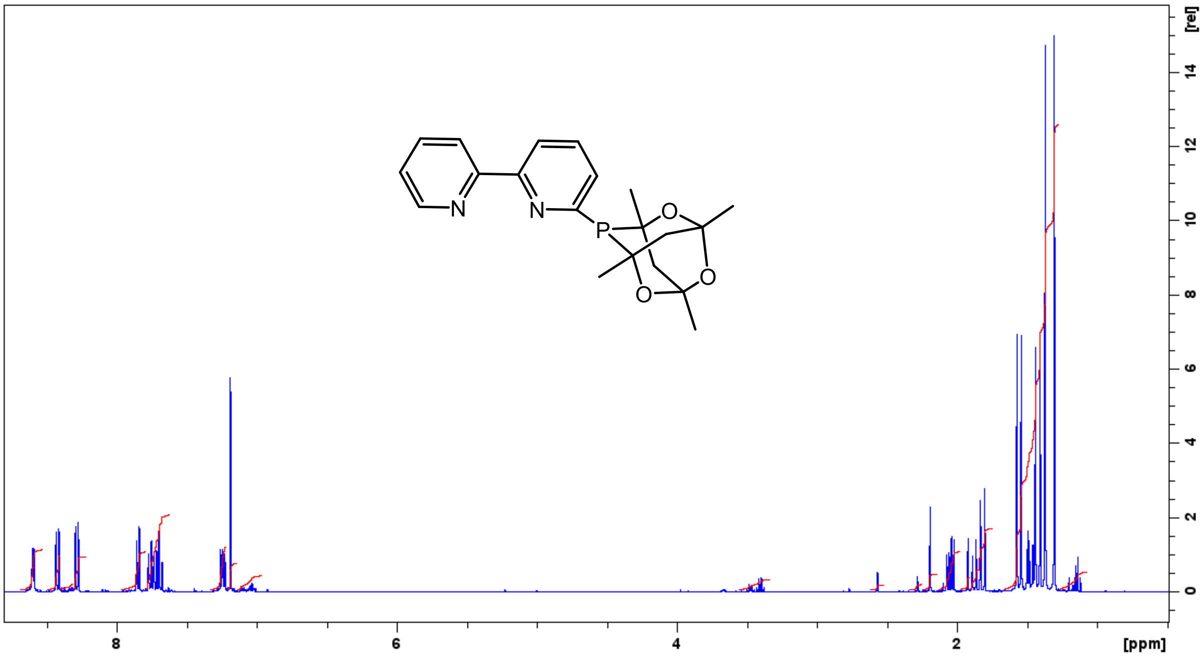

Figure S8. $^{13}C\{^{1}H\}$ NMR spectrum of **6L** recorded at 100 MHz in $CDCl_3$.

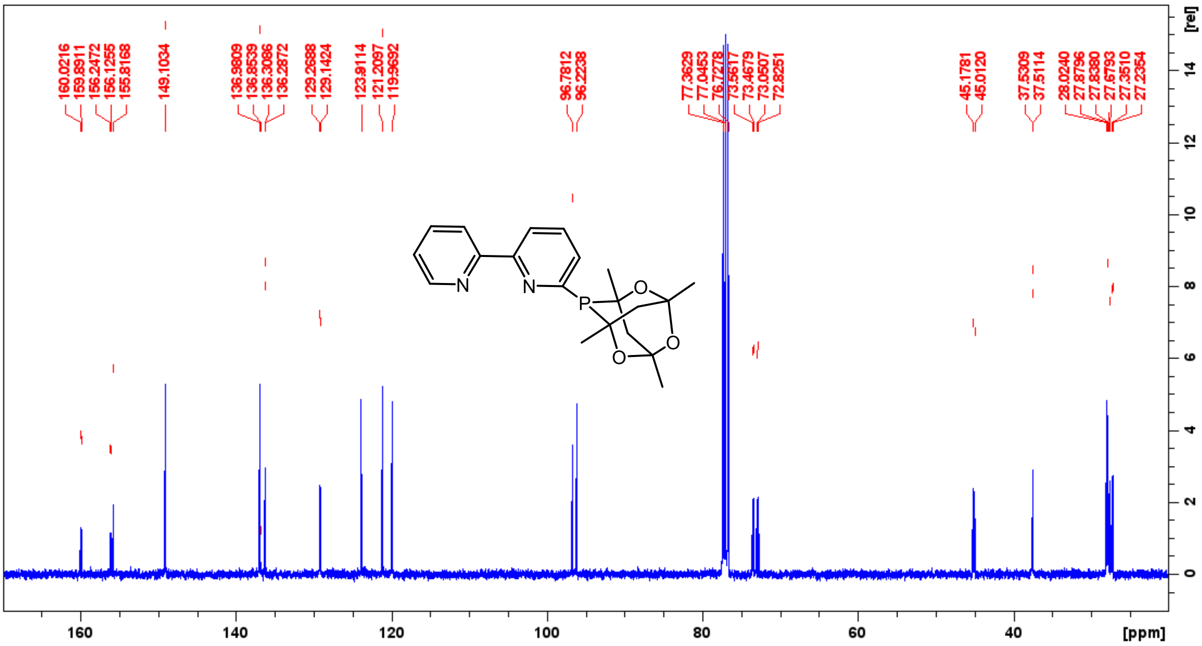

Figure S9. $^{13}C\{^{1}H\}$ DEPT NMR spectrum of **6L** recorded at 100 MHz in $CDCl_3$.

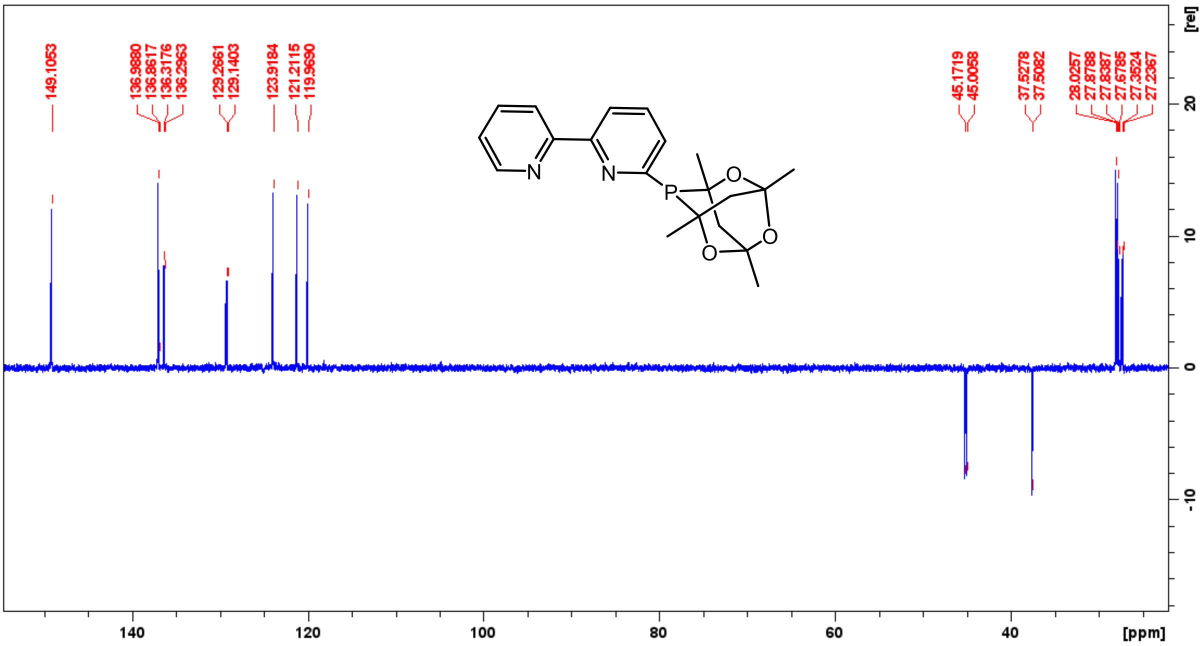

Figure S10. ^{1}H-^{13}C HSQC NMR spectrum of **6L** recorded in $CDCl_3$.

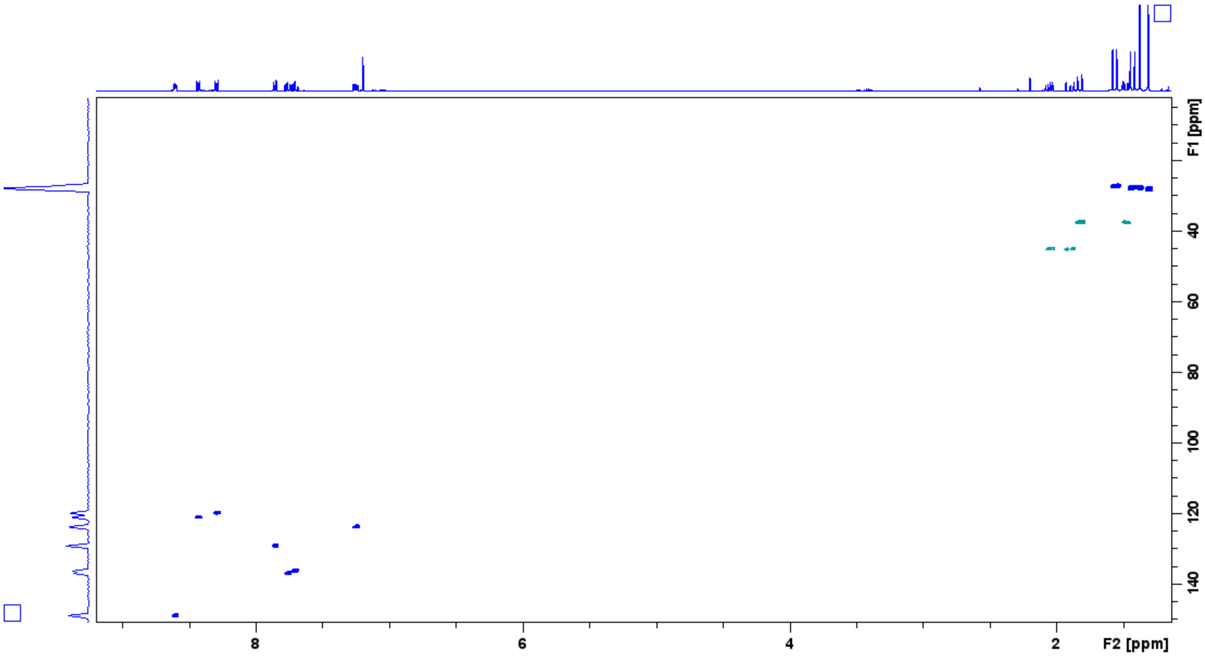

Figure S11. ^1H-^1H COSY NMR spectrum of **6L** recorded at 400 MHz in $CDCl_3$.

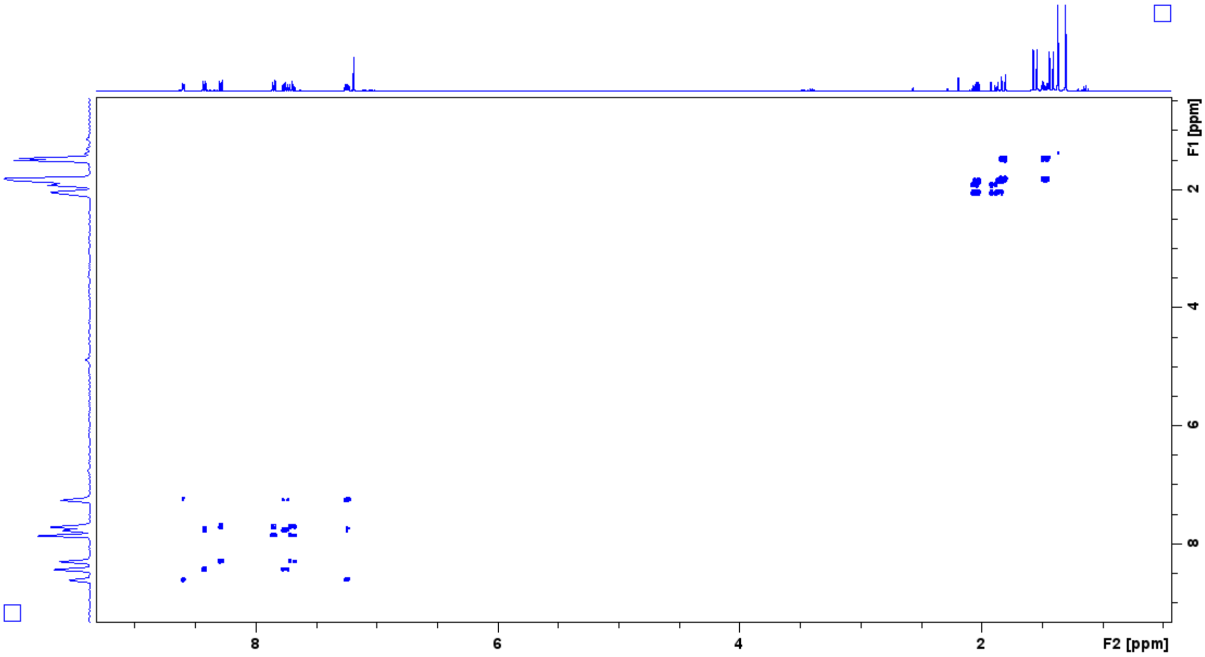

Figure S12. ^1H-^1H NOESY NMR spectrum of **6L** recorded at 400 MHz in $CDCl_3$.

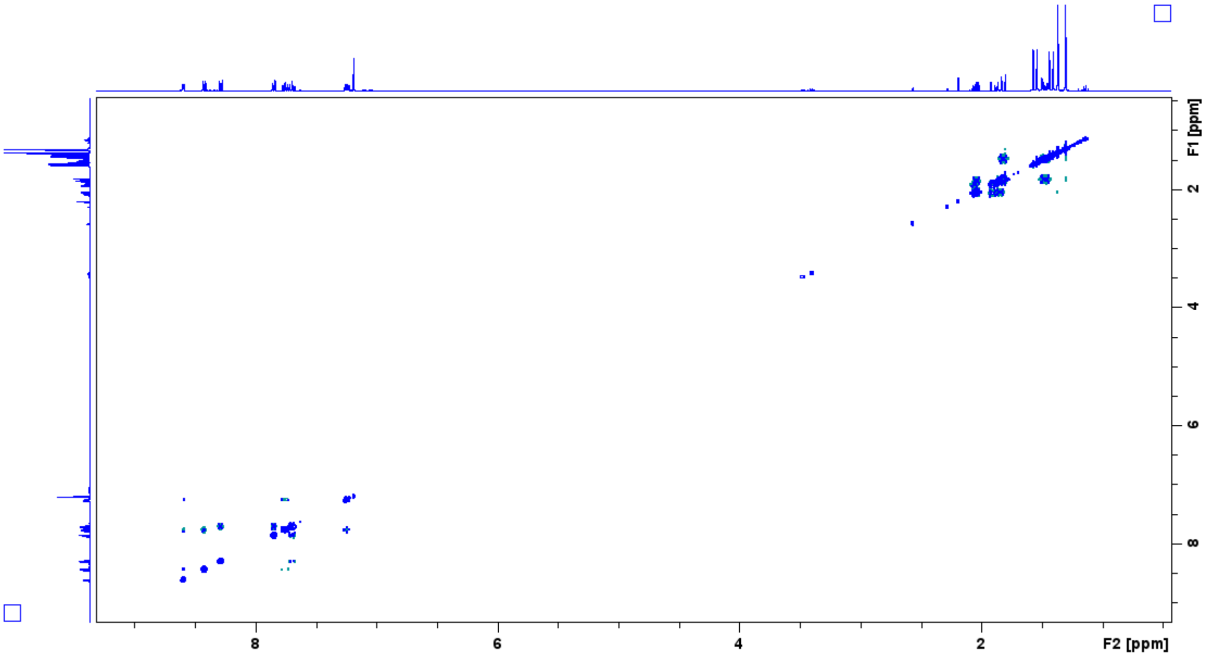

Figure S13. HRMS (ES$^+$) spectrum of **6L**.

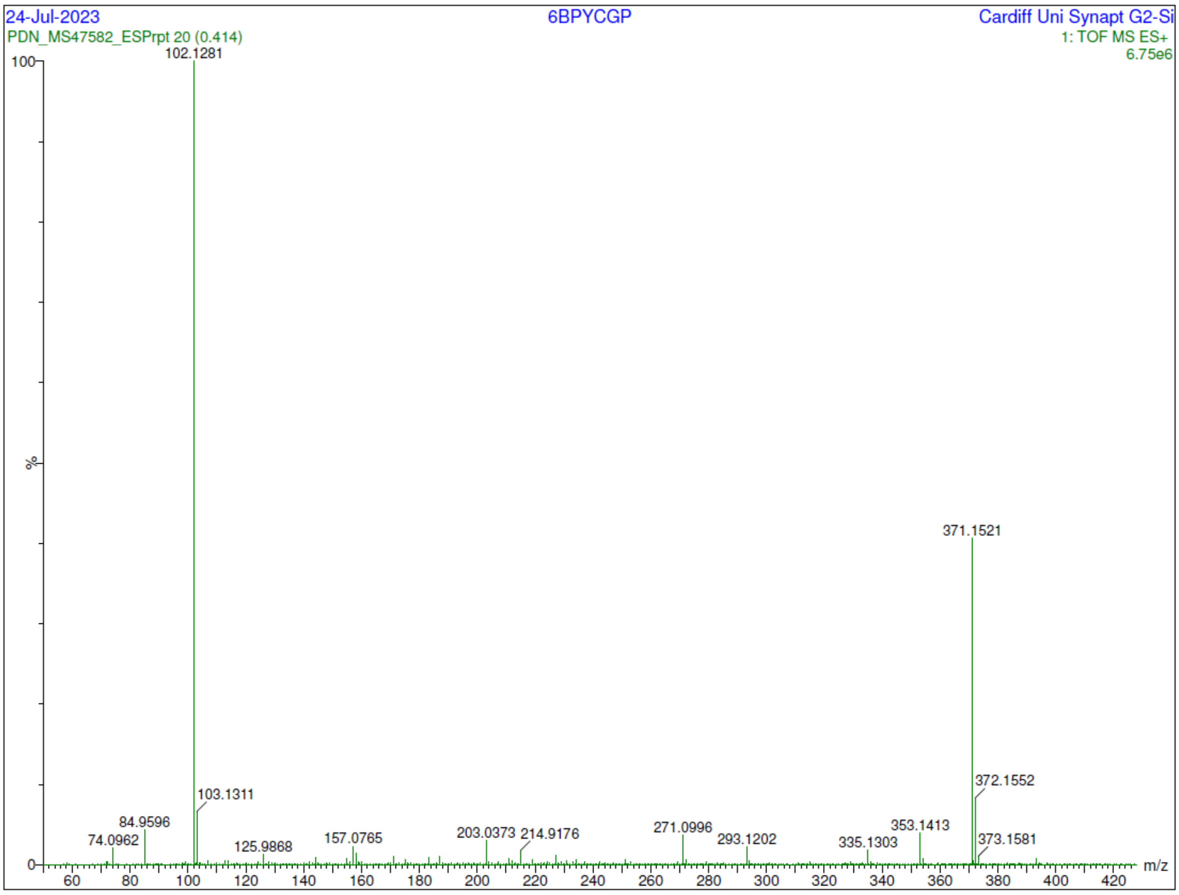

Minimum:				-1.5				
Maximum:		5.0	5.0	50.0				
Mass	Calc. Mass	mDa	PPM	DBE	i-FIT	Norm	Conf(%)	Formula
371.1521	371.1525	-0.4	-1.1	10.5	700.4	n/a	n/a	C20 H24 N2 O3 31P

3. *fac*-[Re(κ[2]-*N,N′*-**5L**)(CO)$_3$Cl], **5L^{Re}**.

Figure S14. ^{31}P{^{1}H} NMR spectrum of **5L^{Re}** recorded at 162 MHz in D$_6$-acetone.

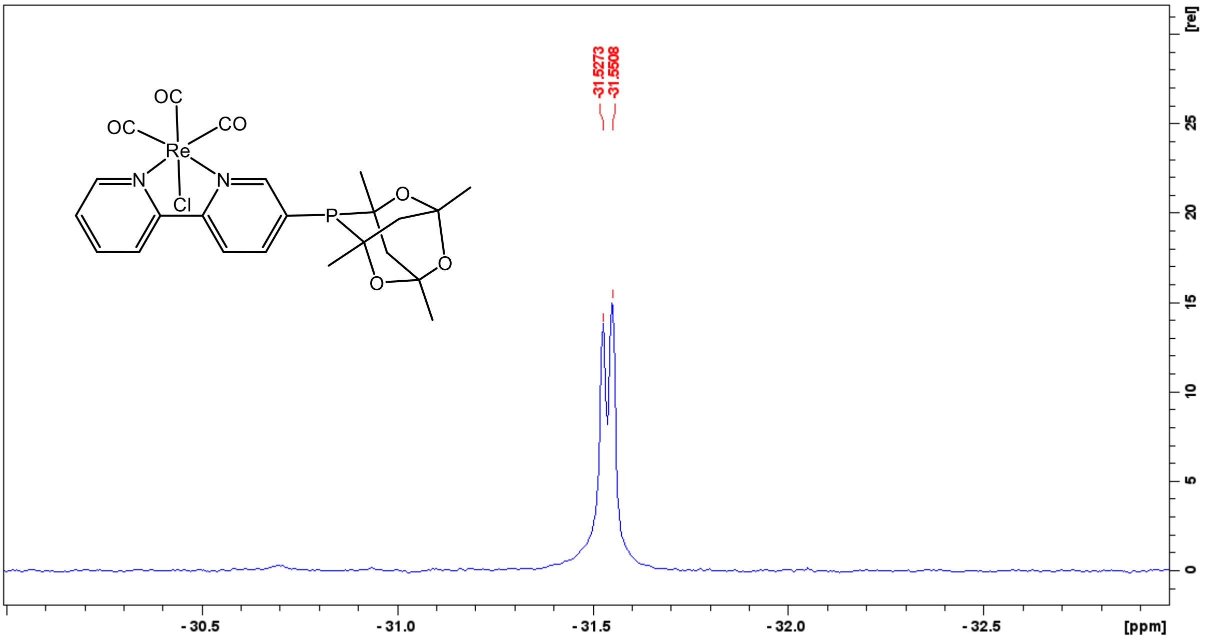

Figure S14b. ^{31}P{^{1}H} NMR spectrum of oxidised **5L^{Re}** recorded at 162 MHz in D$_6$-acetone.

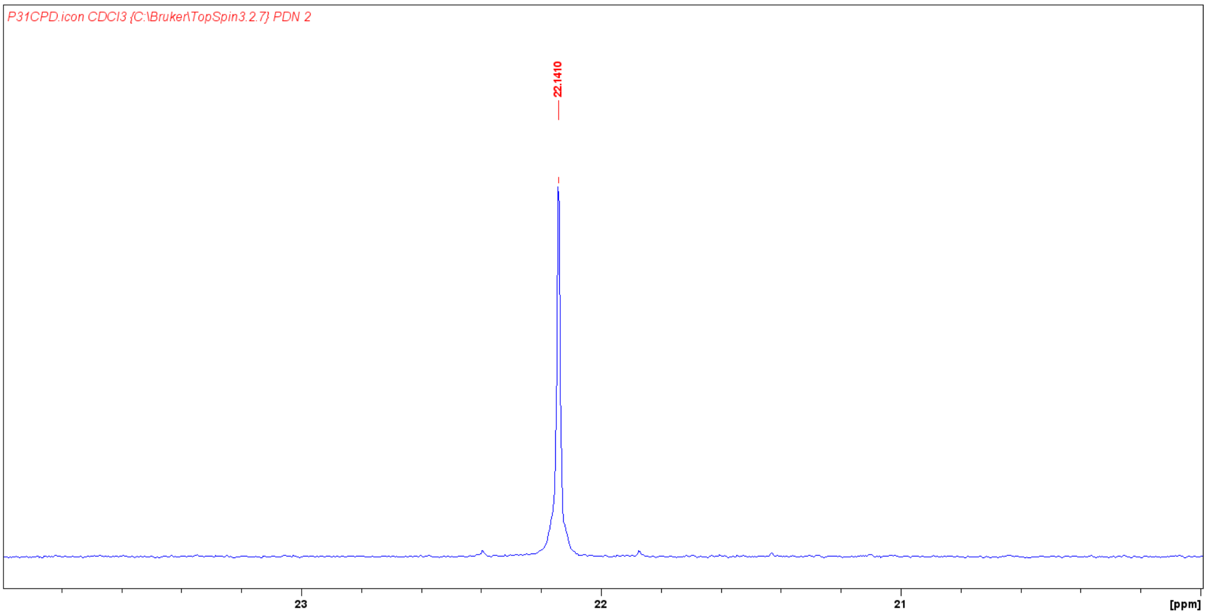

Figure S15. ^{1}H NMR spectrum of **5L^{Re}** recorded at 500 MHz in D$_6$-acetone.

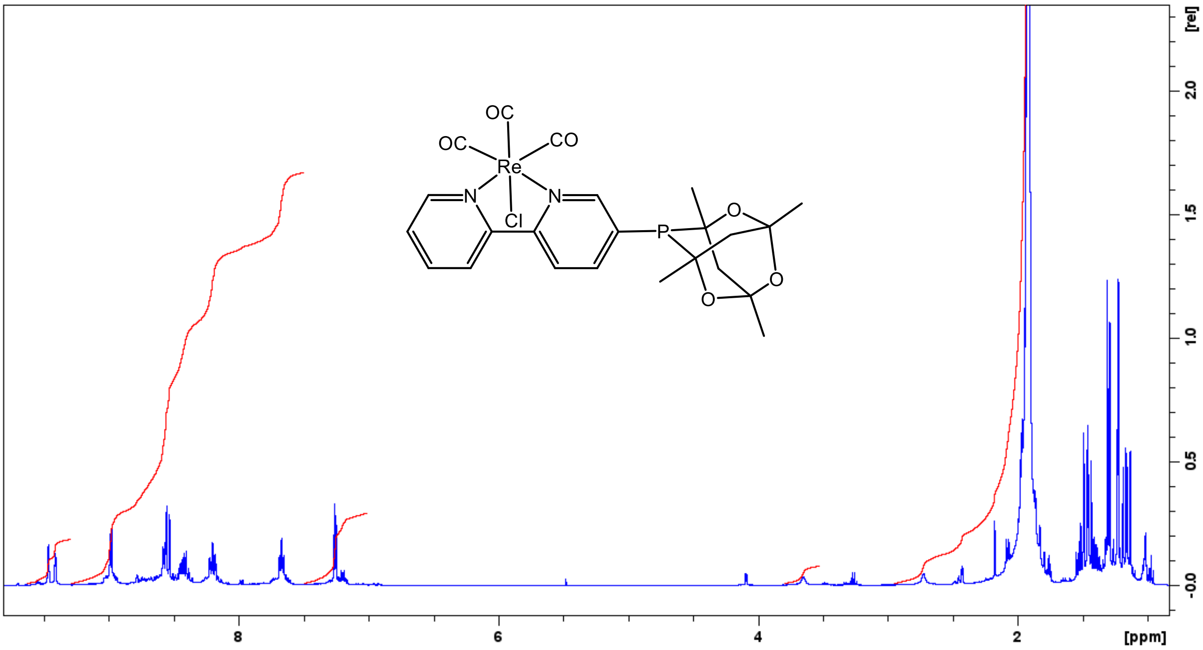

Figure S16. $^{13}C\{^{1}H\}$ NMR spectrum of **5L^{Re}** recorded at 150.9 MHz in C_6D_6.

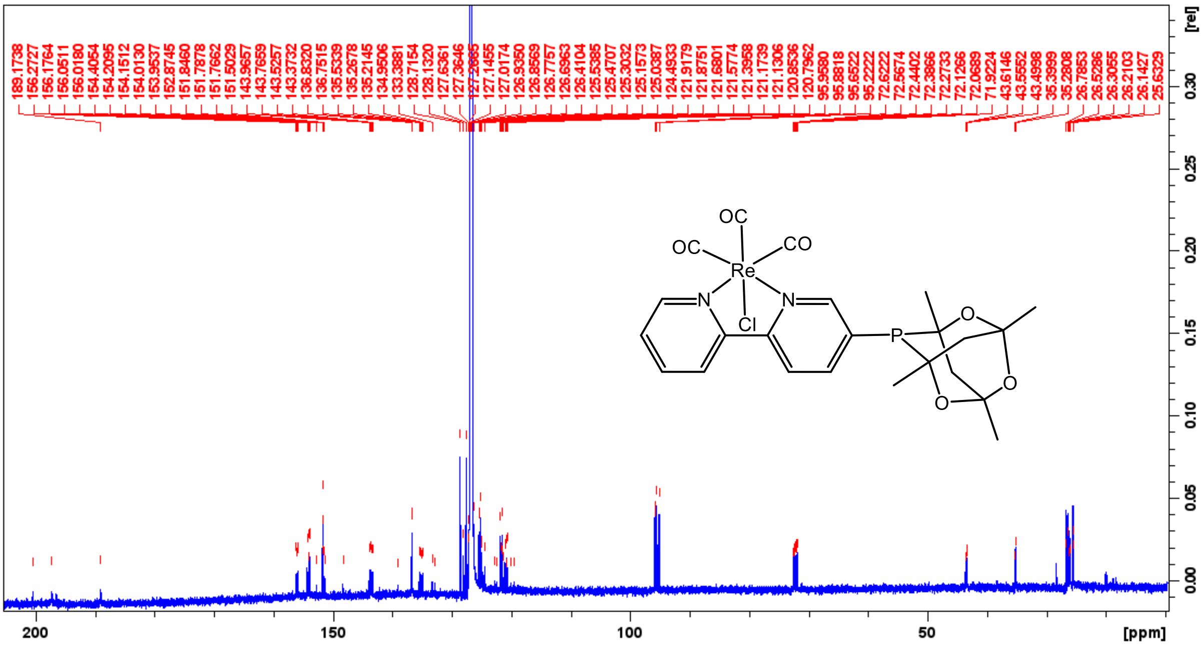

Figure S17. ^{13}C-^{1}H HSQC NMR spectrum of **5L^{Re}** recorded in C_6D_6.

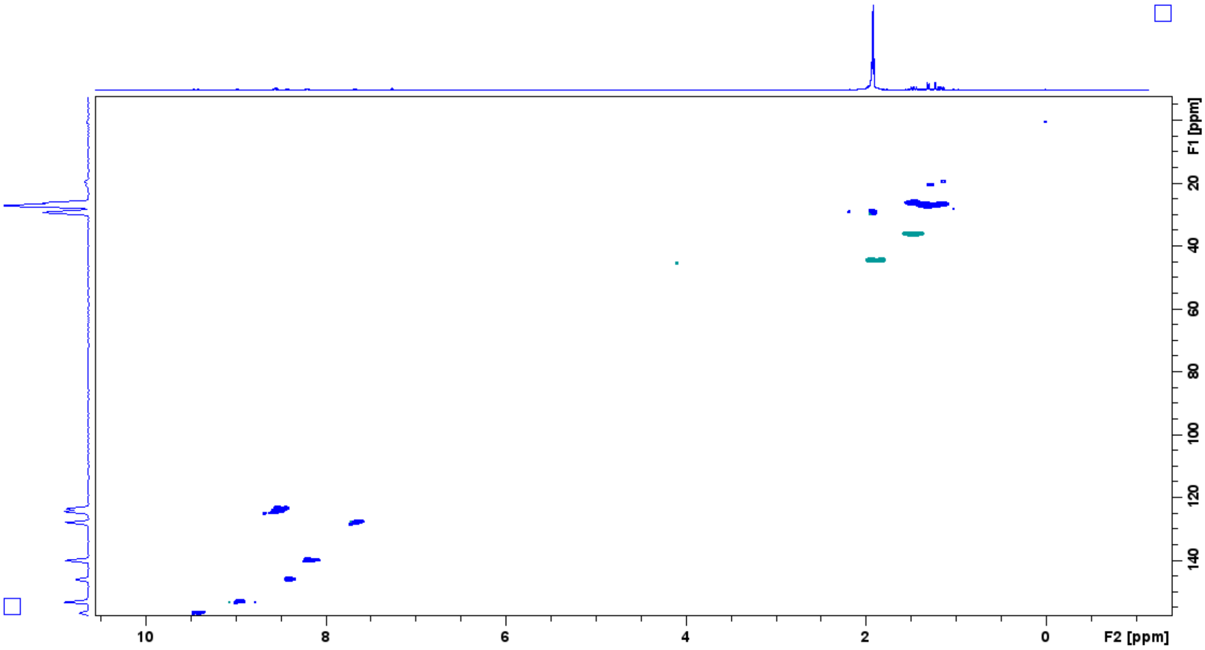

Figure S18. ^{1}H-^{1}H COSY NMR spectrum of **5L^{Re}** recorded in C_6D_6.

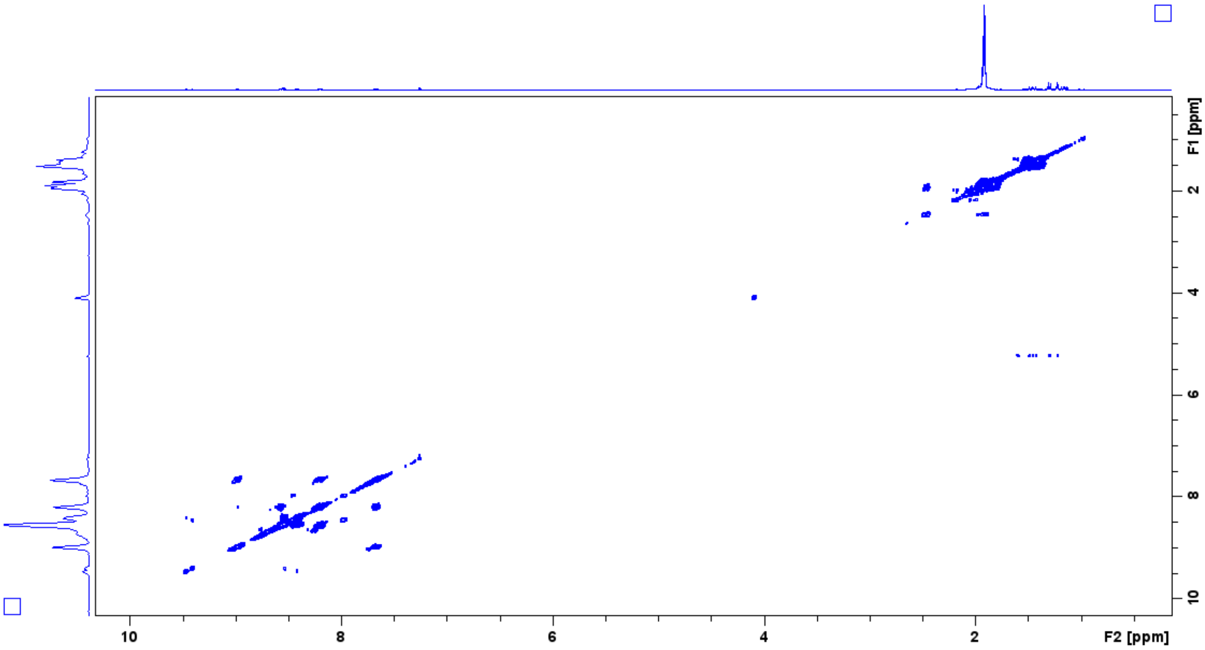

Figure S19. HRMS (ES^{+}) spectrum of **5L^{Re}**.

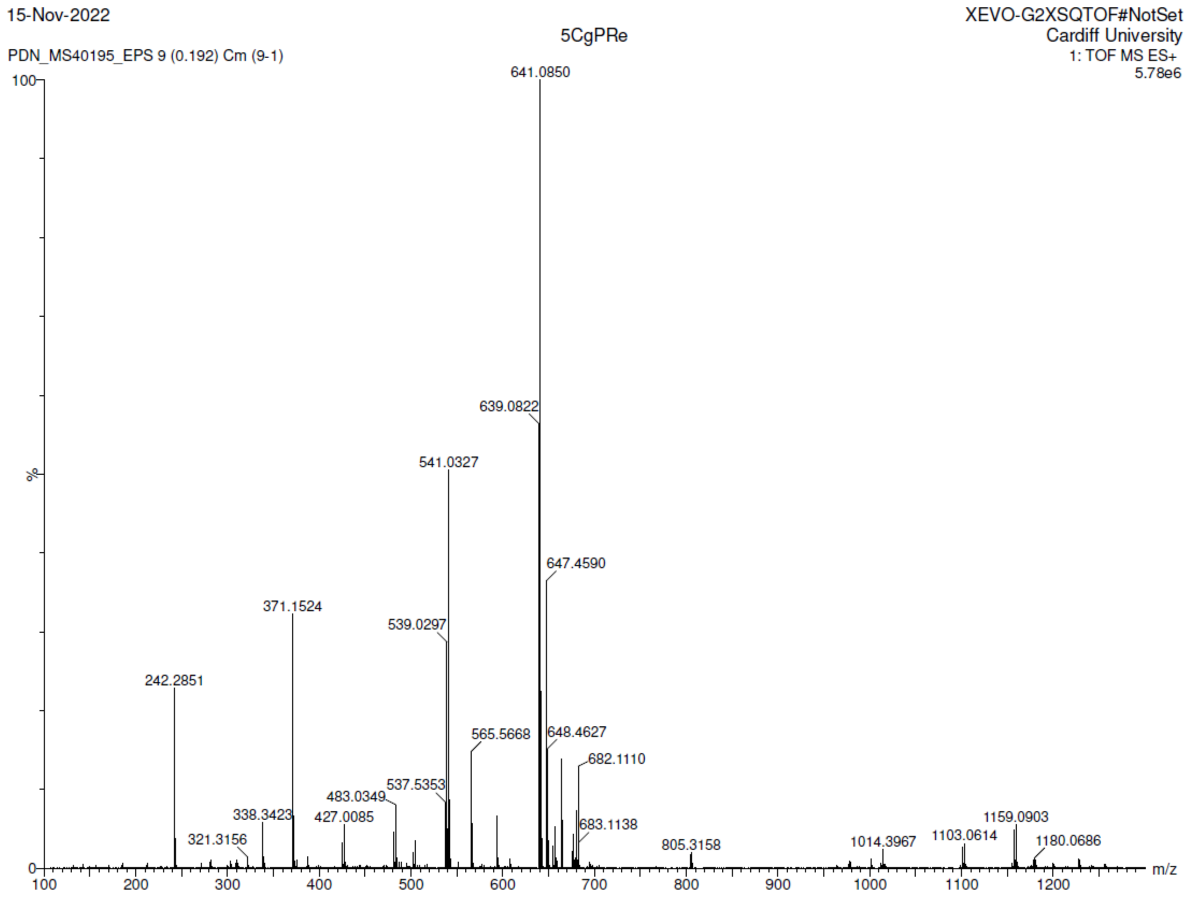

	Mass	Calc. Mass	mDa	PPM	DBE	i-FIT	Norm	Conf(%)	Formula
Minimum:					-1.5				
Maximum:			5.0	5.0	100.0				
	641.0850	641.0851	-0.1	-0.2	14.0	684.4	n/a	n/a	C23 H23 N2 O6 P Re

4. *fac*-[Re(κ^2-*N,N′*-**6L**)(CO)$_3$Cl], **6L^{Re}**.

Figure S20. $^{31}P\{^1H\}$ NMR spectrum of **6L^{Re}** recorded at 162 MHz in D$_6$-dmso.

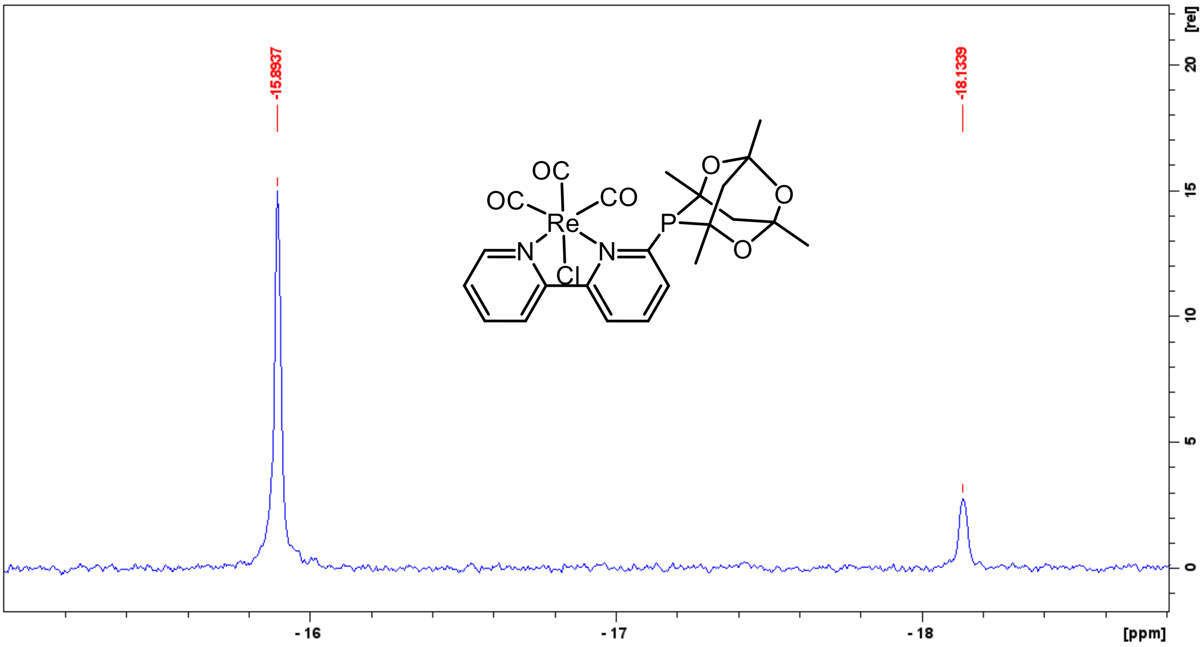

Figure S21. ^{1}H NMR spectrum of **6L^{Re}** recorded at 400 MHz in D_6-dmso.

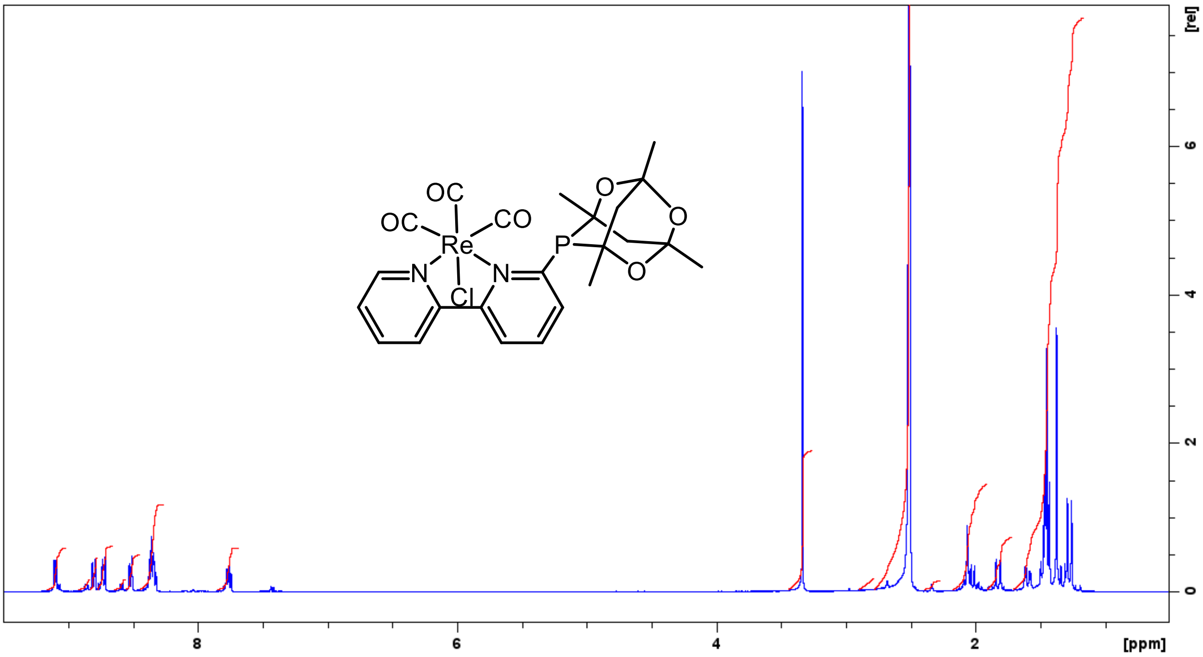

Figure S22. ^{13}C NMR spectrum of **6L^{Re}** recorded at 100 MHz in D_6-dmso. (this is ~1:1 isomer mixture)

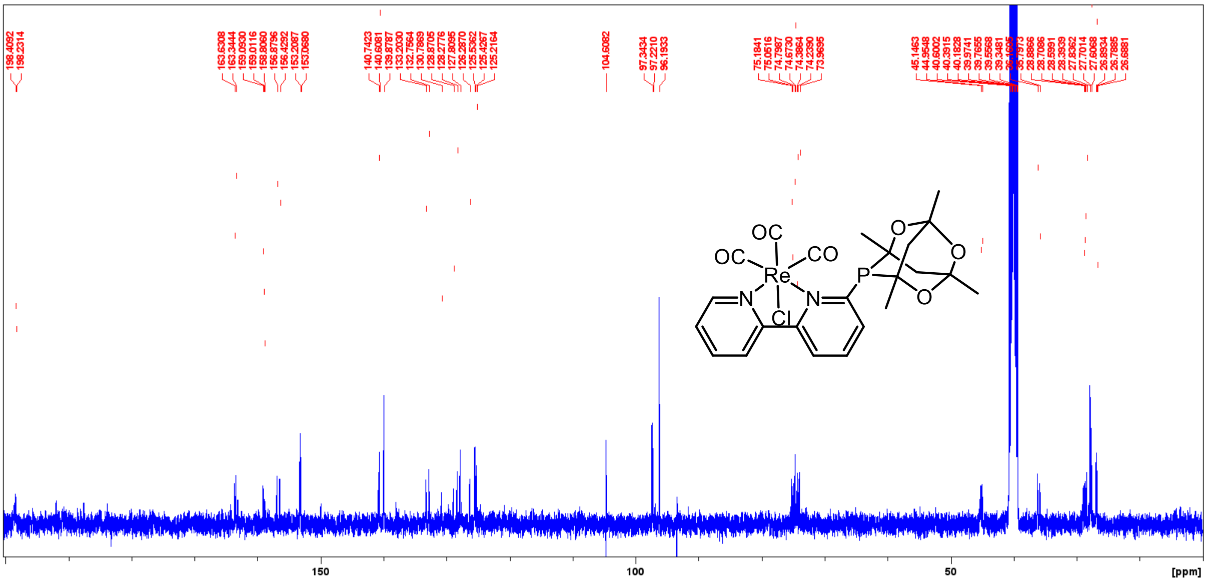

Figure S23. ^{13}C NMR DEPT spectrum of **6L^{Re}** recorded at 100 MHz in D$_6$-dmso. (this is ~1:1 isomer mixture)

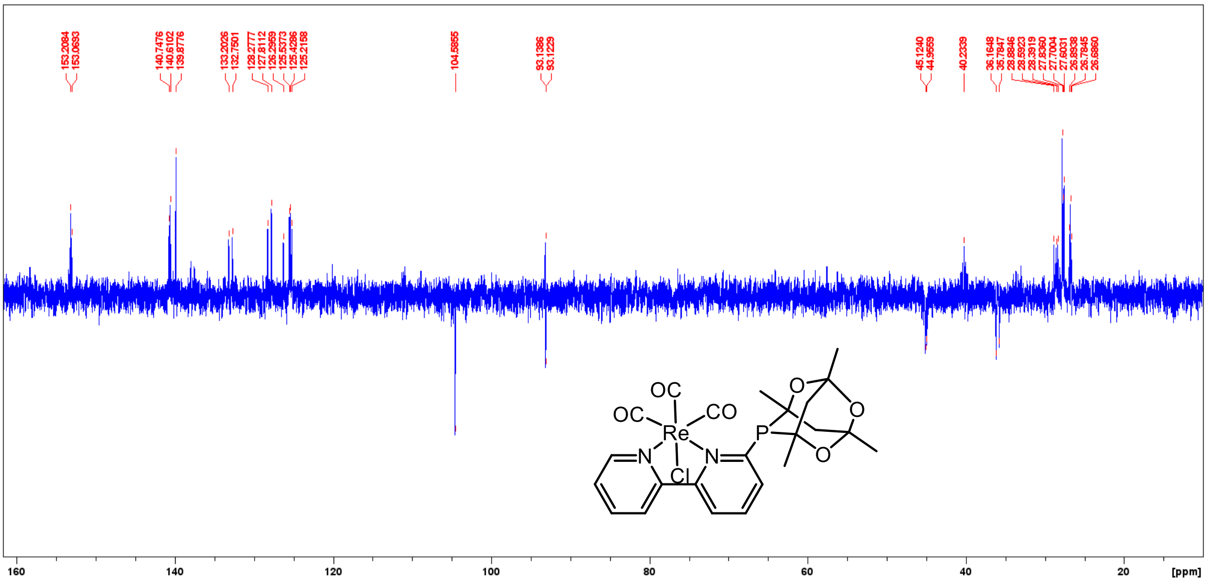

Figure S24. ^{13}C-^{1}H HSQC NMR spectrum of **6L^{Re}** recorded in D$_6$-dmso.

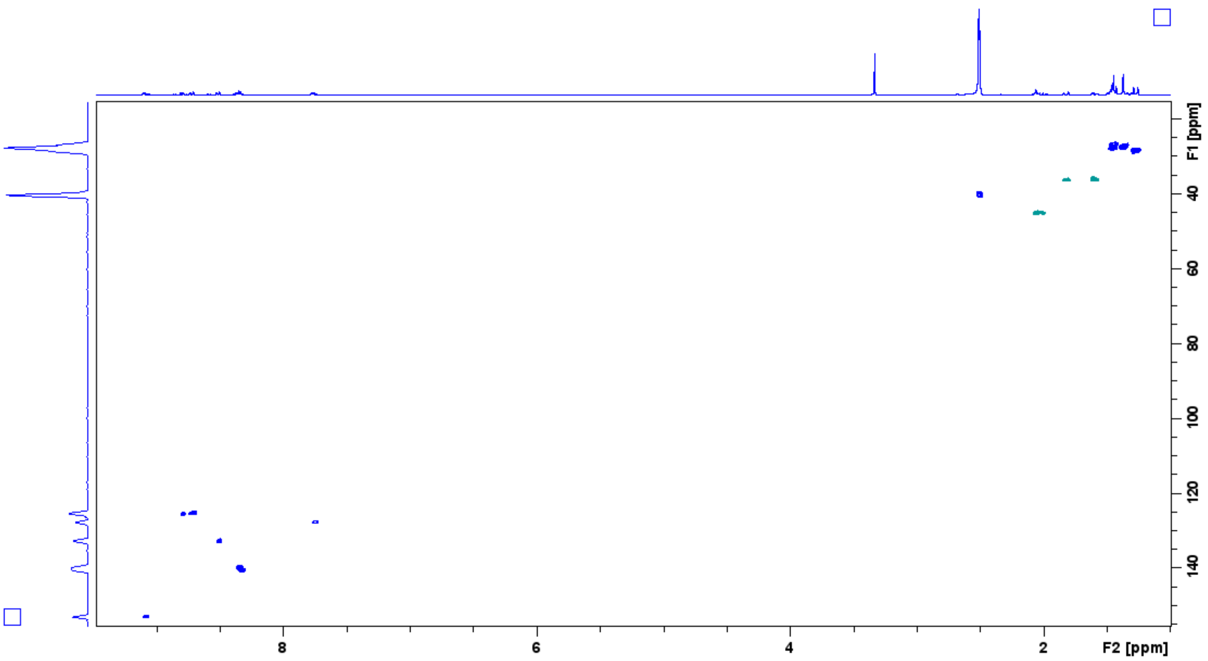

Figure S25. ^{1}H-^{1}H COSY NMR spectrum of **6L^{Re}** recorded in D_6-dmso.

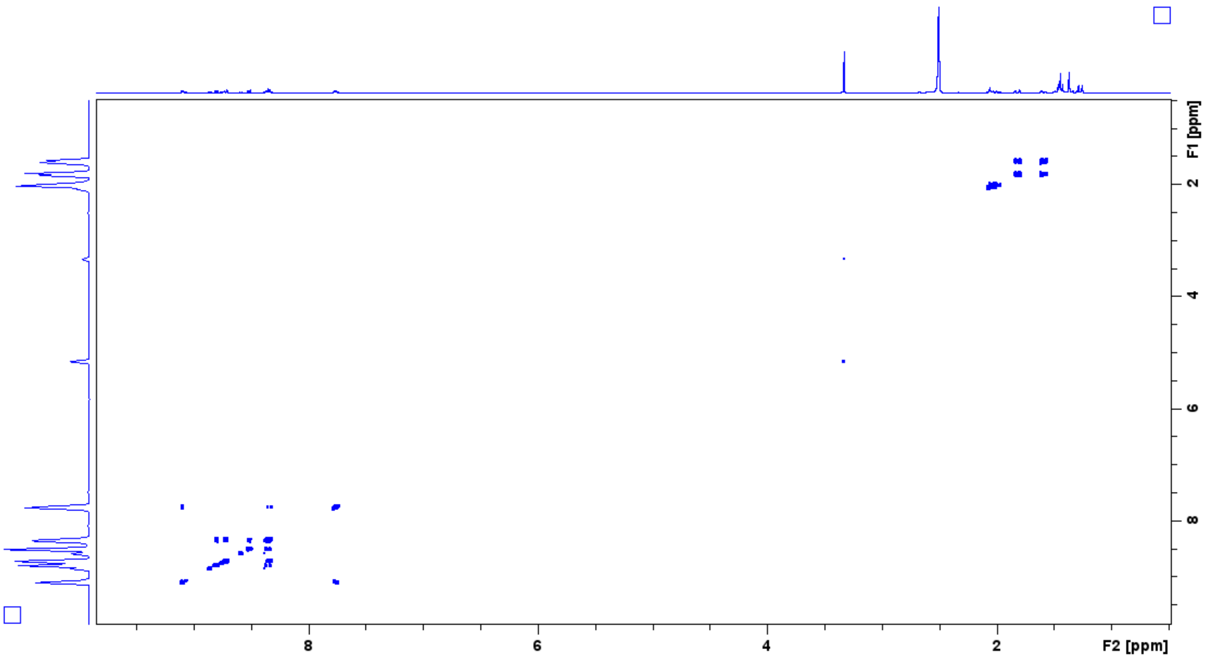

Figure S26. HRMS (ES^{+}) spectrum of **6L^{Re}**.

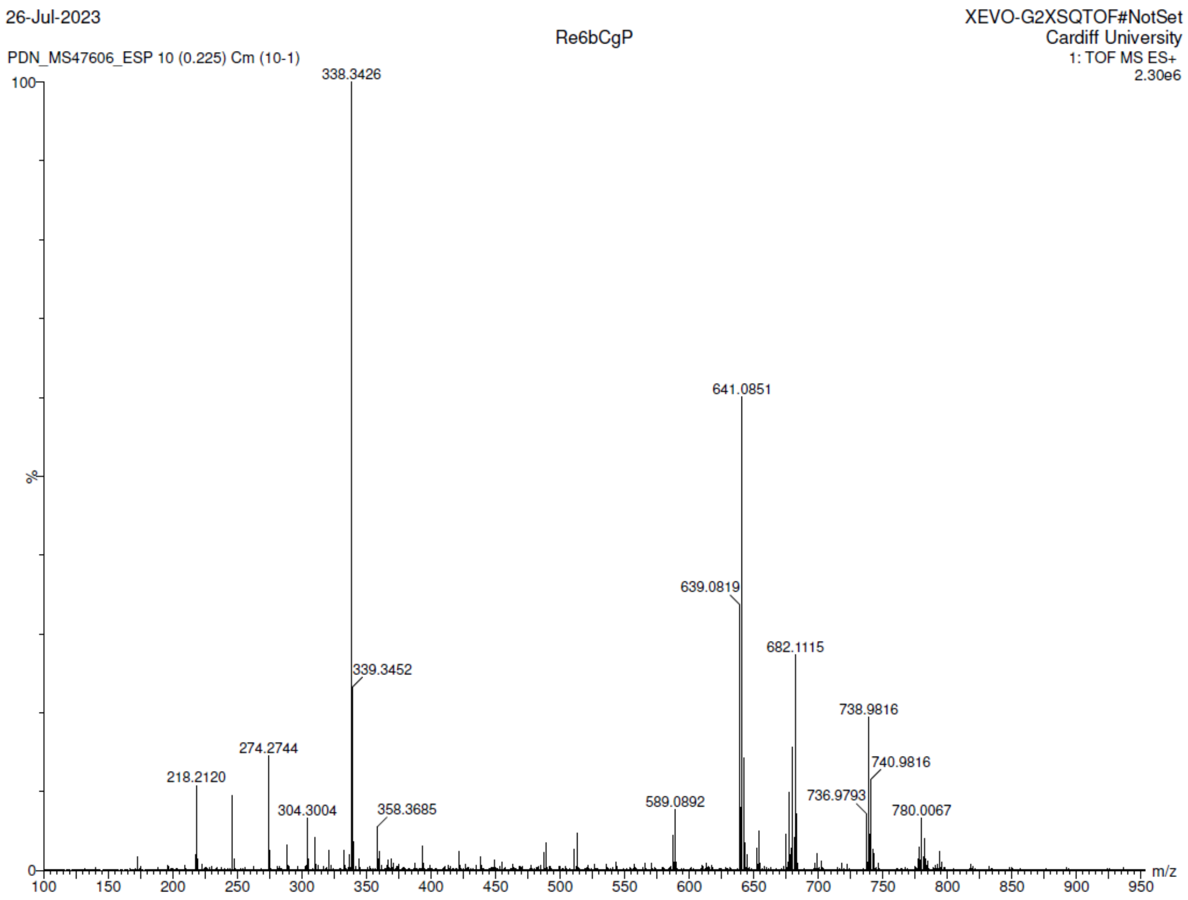

Minimum:				-1.5				
Maximum:		5000.0	10.0	50.0				
Mass	Calc. Mass	mDa	PPM	DBE	i-FIT	Norm	Conf(%)	Formula
639.0819	639.0823	-0.4	-0.6	14.0	868.2	n/a	n/a	C23 H23 N2 O6 185Re P

5. *Fac*-[Re(κ^2-*N,N'*-Re,κ-*P*-Au-**5L**)(CO)$_3$Cl(AuCl)]

Figure S27. ^{31}P{^{1}H} NMR spectrum of *syn,anti*-**5L**$^{\mathbf{Re,Au}}$ recorded at 202 MHz in D$_6$-acetone.

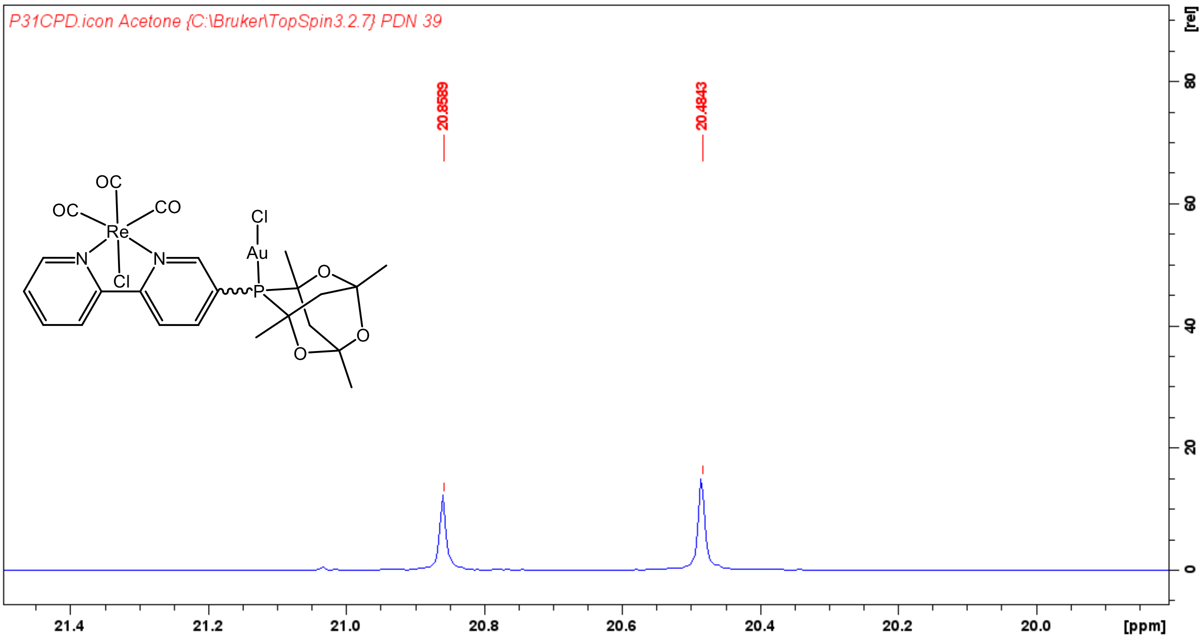

Figure S28. ^{1}H NMR spectrum of *syn,anti*-**5L**$^{\mathbf{Re,Au}}$ recorded at 500 MHz in D_6-acetone.

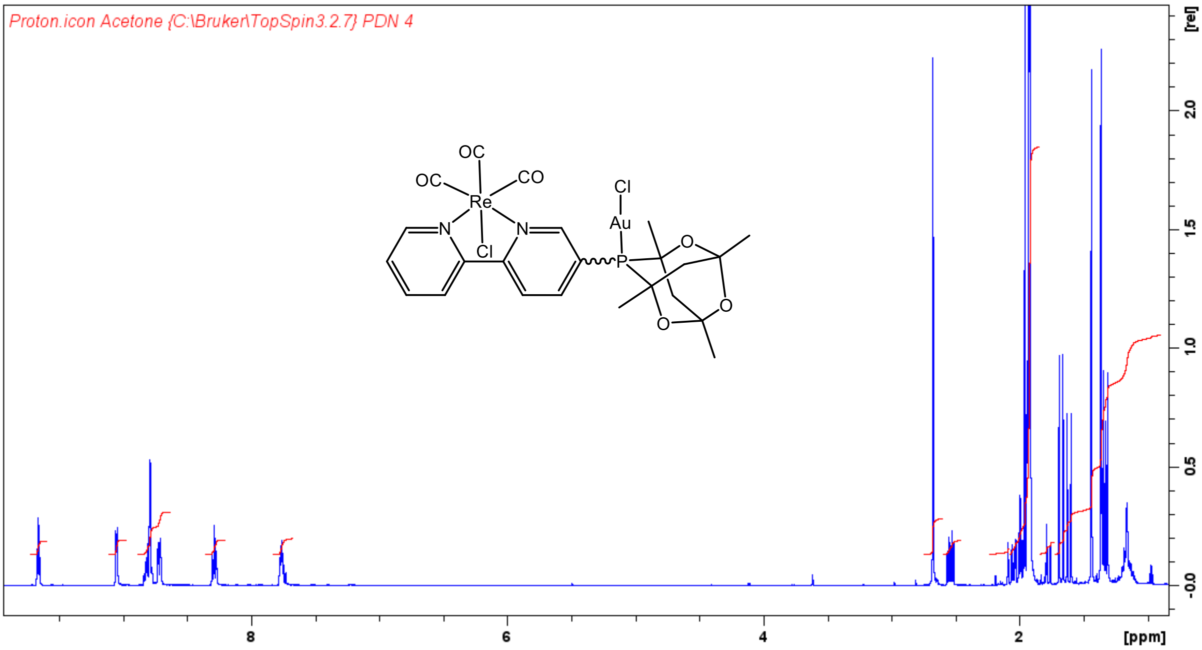

Figure S29. ^{13}C{^{1}H} NMR spectrum of *syn,anti*-**5L**$^{\mathbf{Re,Au}}$ recorded at 125 MHz in D_6-acetone.

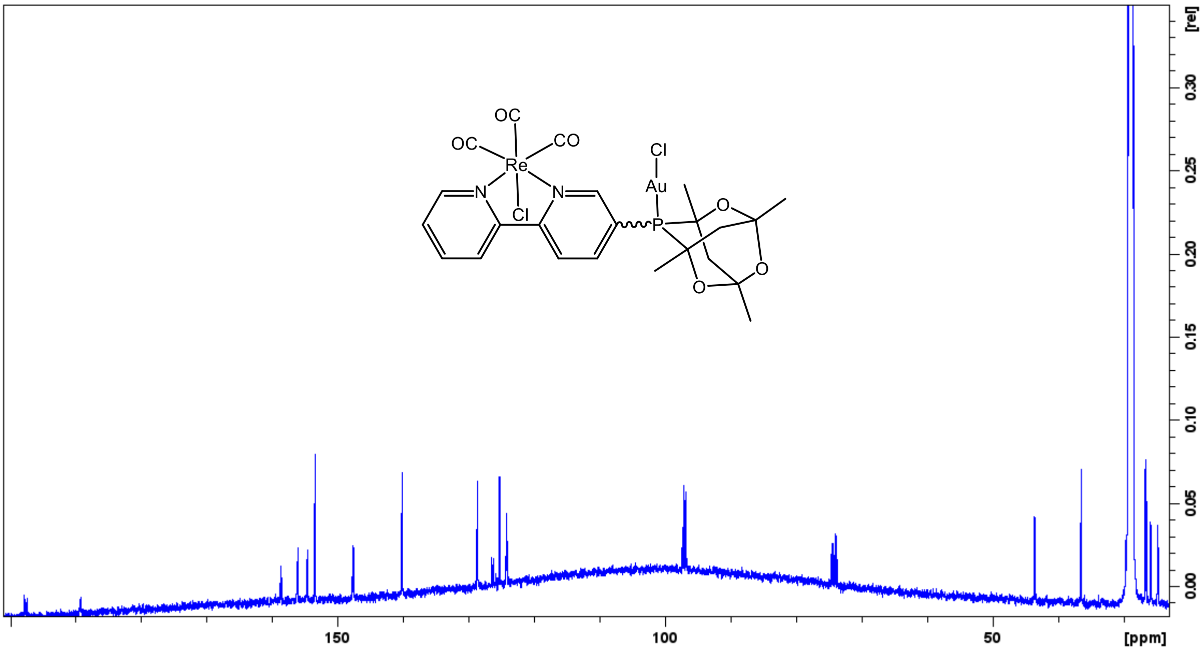

Figure S30. $^{13}C\{^{1}H\}$ DEPT NMR spectrum of *syn,anti*-**5L**^{Re,Au} recorded at 100 MHz in D_6-acetone.

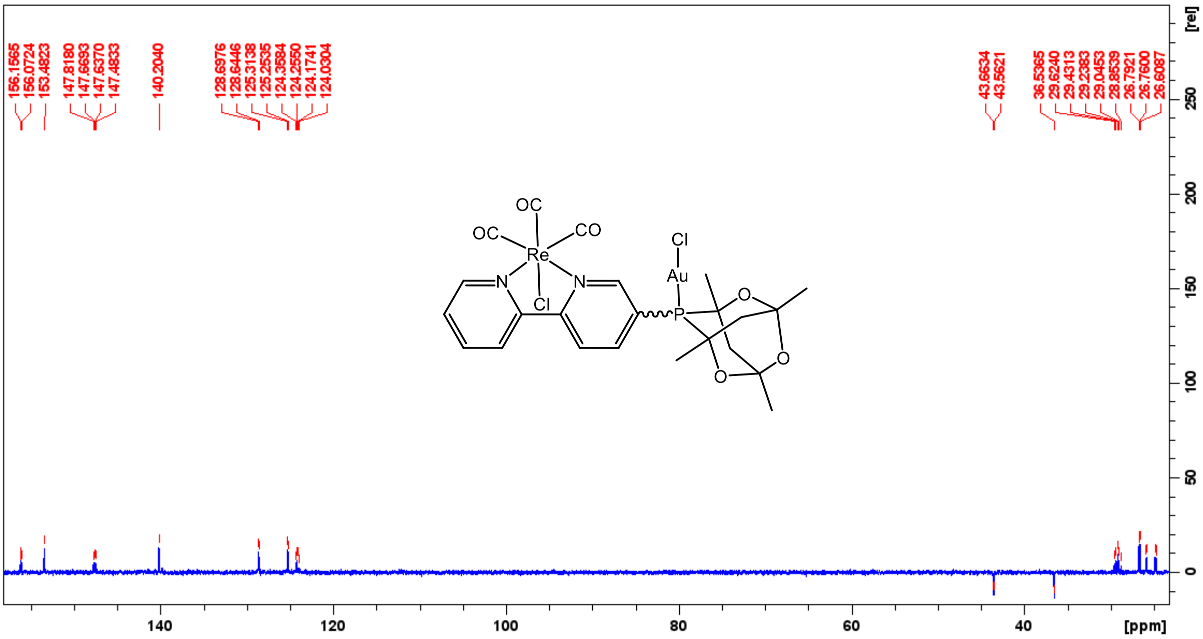

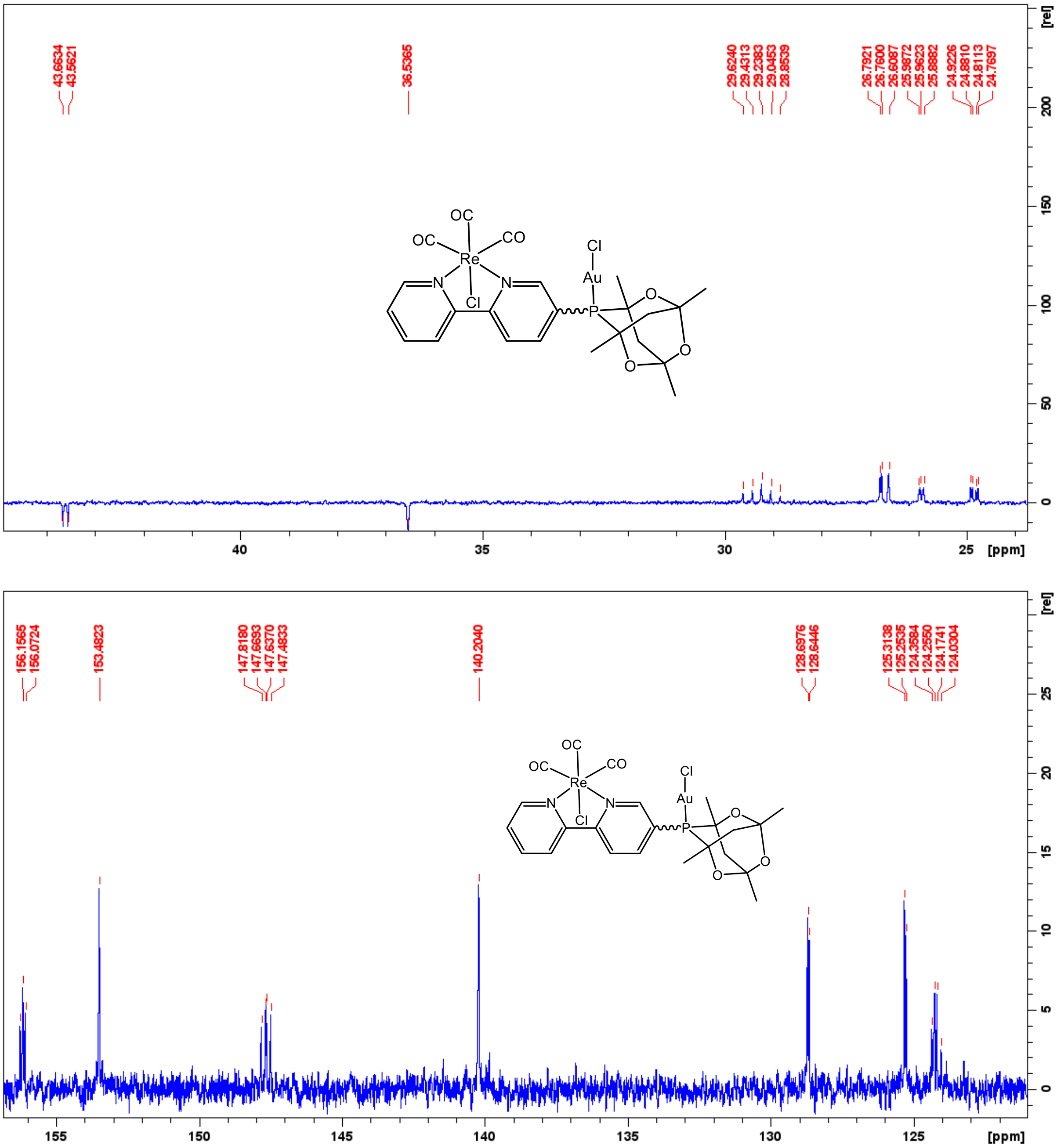

Figure S31. ^{13}C-^{1}H HSQC NMR spectrum of *syn,anti*-**5L**$^{\mathbf{Re,Au}}$ recorded in D_6-acetone.

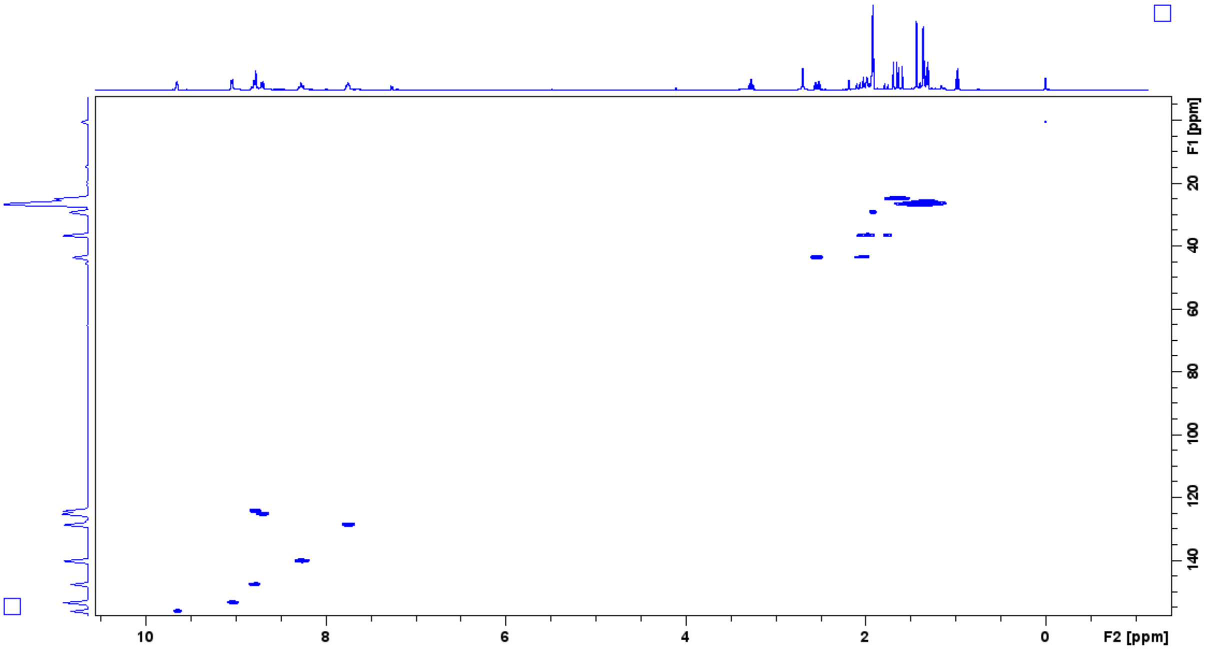

Figure S32. ^{1}H-^{1}H COSY NMR spectrum of *syn,anti*-**5L**^{Re,Au} recorded in D_6-acetone.

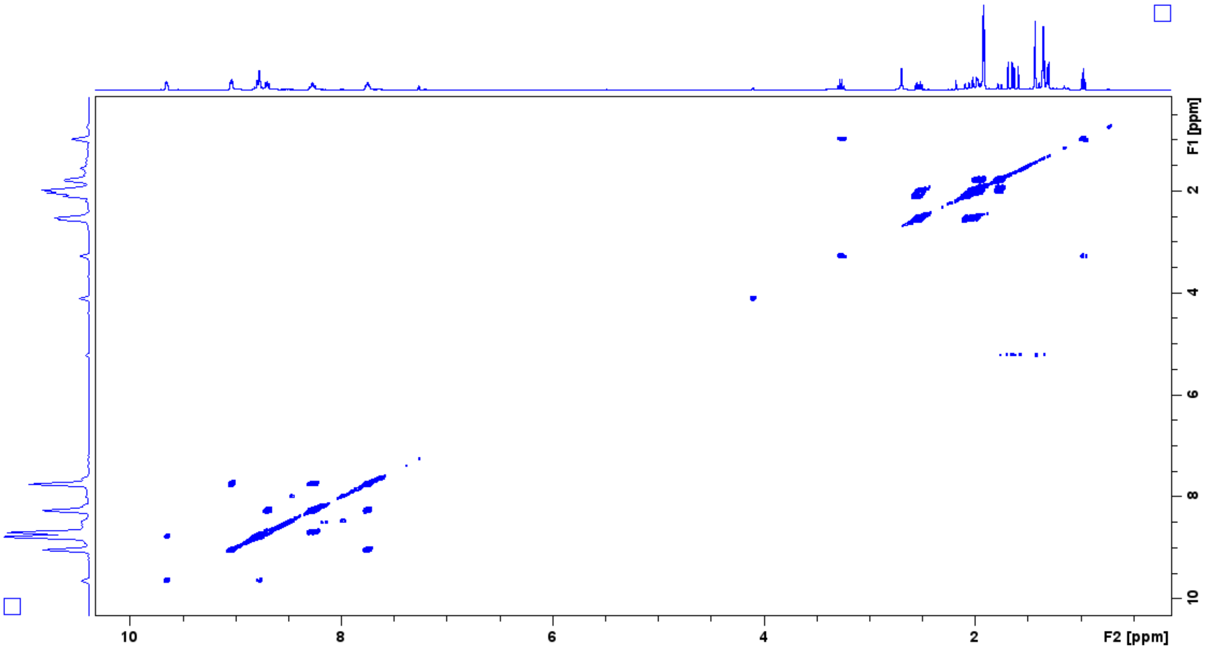

Figure S33. ^{1}H-^{1}H NOESY NMR spectrum of *syn,anti*-**5L**^{Re,Au} recorded in D_6-acetone.

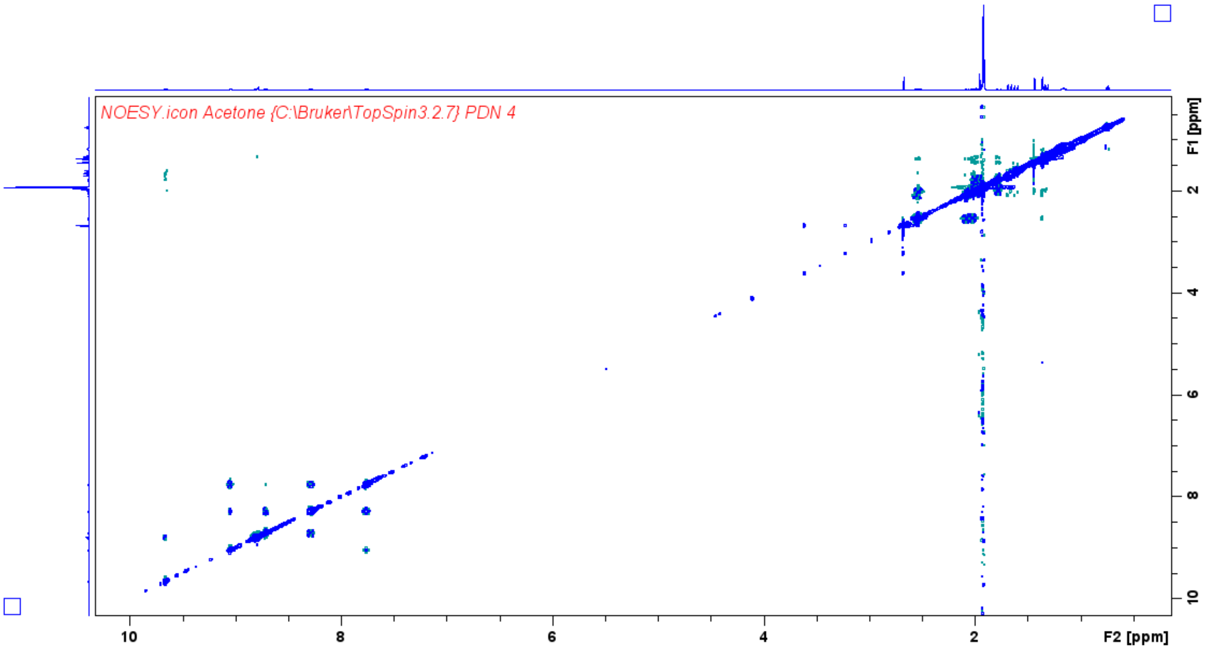

Figure S34. LRMS (ES$^+$) spectrum of *syn,anti*-**5L^{Re,Au}**.

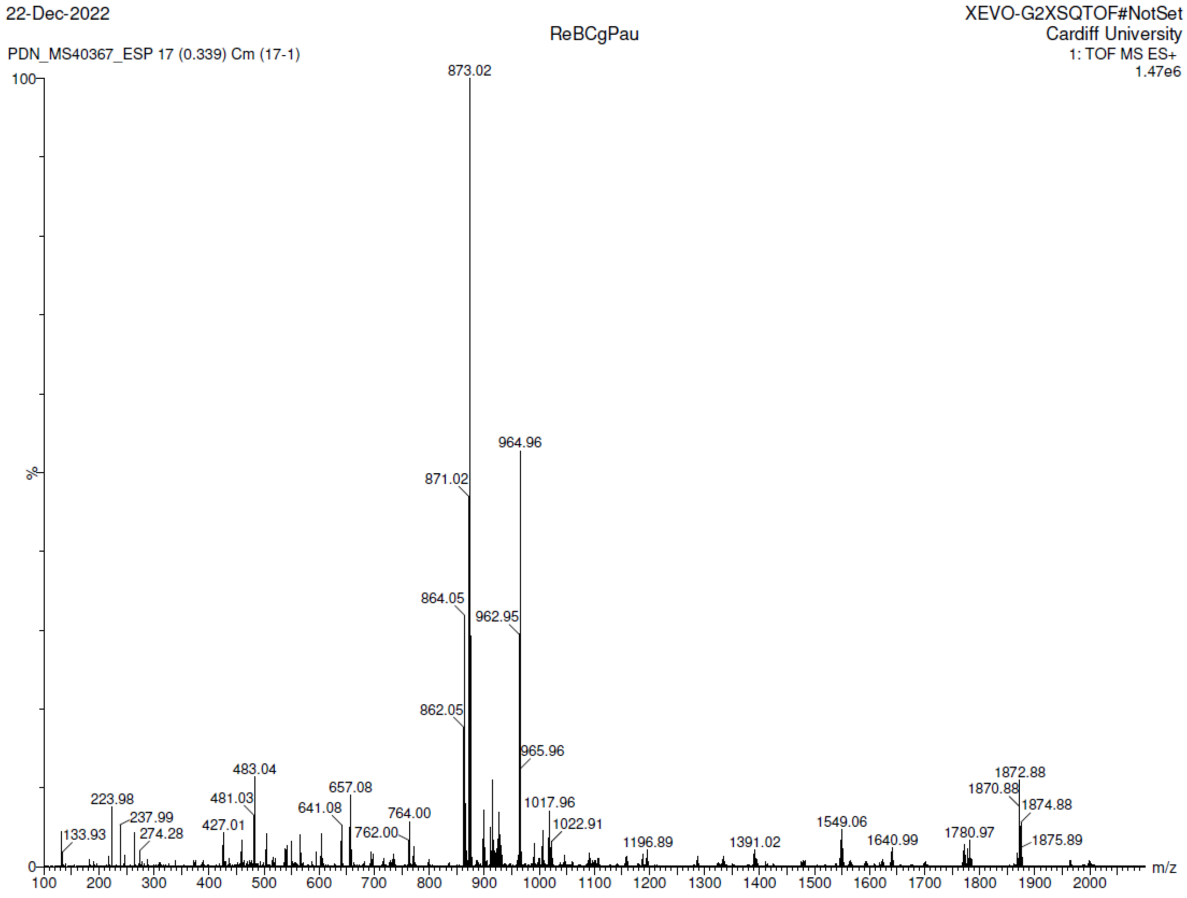

Figure S35. HRMS (ES$^+$) spectrum of *syn,anti*-**5L^{Re,Au}**.

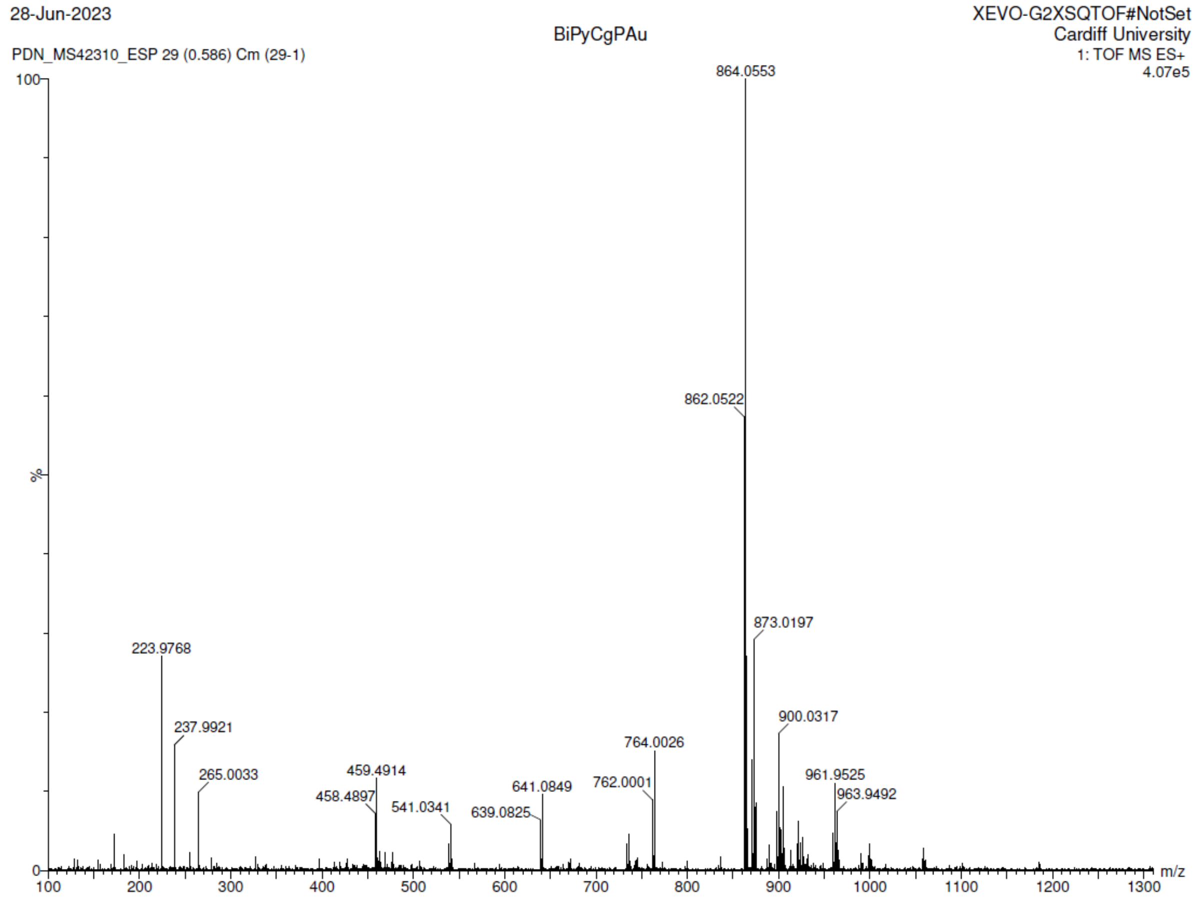

	Mass	Calc. Mass	mDa	PPM	DBE	i-FIT	Norm	Conf(%)	Formula
Minimum:					-1.5				
Maximum:			5000.0	5.0	50.0				
	873.0197	873.0205	-0.8	-0.9	14.0	595.6	n/a	n/a	C23 H23 N2 O6 P Cl 187Re 197Au

Figure S36. $^{31}P\{^{1}H\}$ NMR spectrum of *anti*-**5L**^{Re,Au} recorded at 202 MHz in $CDCl_3$.

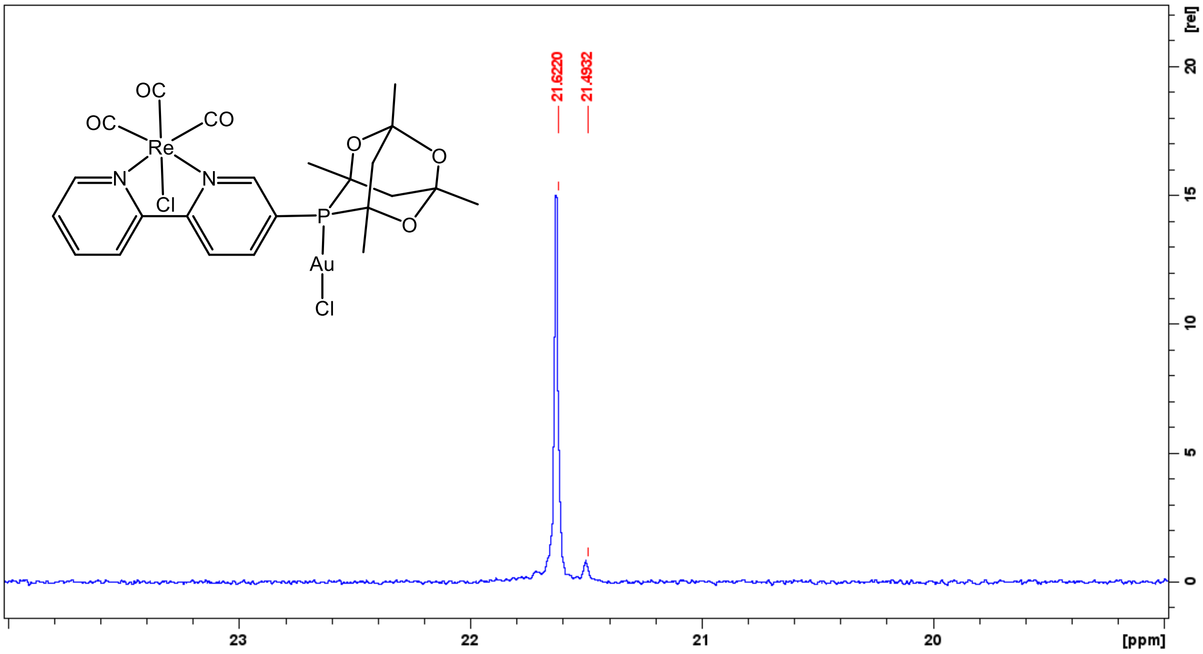

Figure S37. ^{1}H NMR spectrum of *anti*-**5L**$^{\mathbf{Re,Au}}$ recorded at 500 MHz in $CDCl_3$.

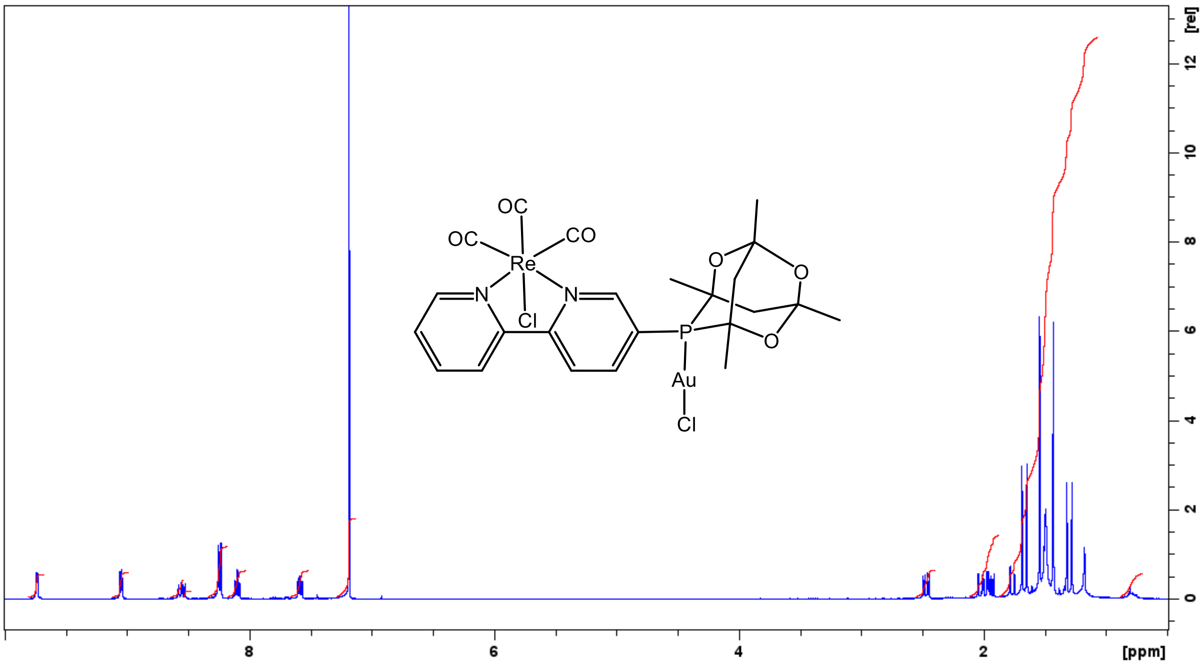

Figure S38. ^{13}C-^{1}H HSQC NMR spectrum of *anti*-**5L**$^{\mathbf{Re,Au}}$ recorded in $CDCl_3$.

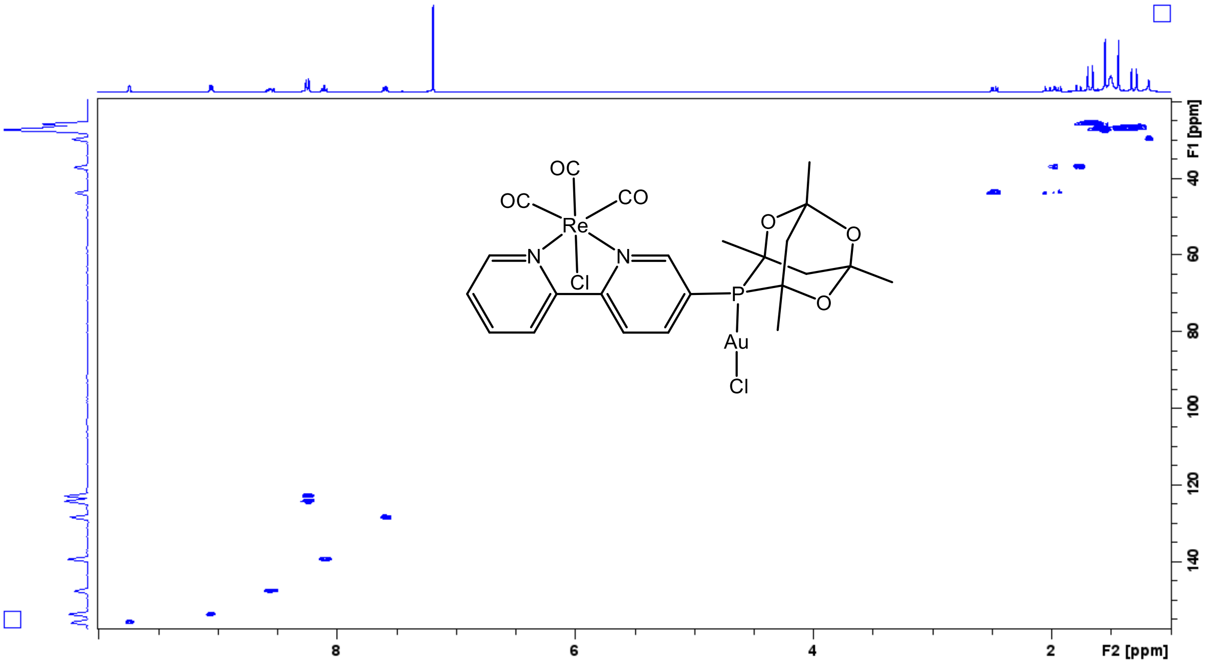

Figure S39. ^{1}H-^{1}H COSY NMR spectrum of *anti*-**5L**^{Re,Au} recorded in $CDCl_3$.

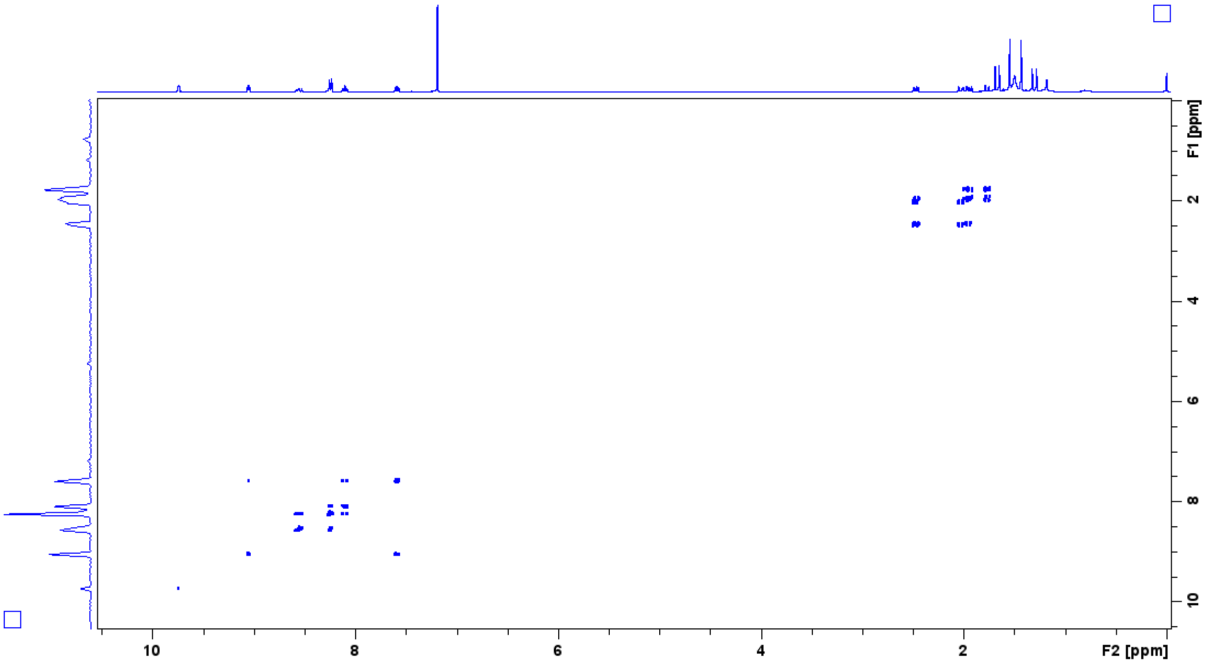

Figure S40. ^{1}H-^{1}H NOESY NMR spectrum of *anti*-**5L**^{Re,Au} recorded in $CDCl_3$.

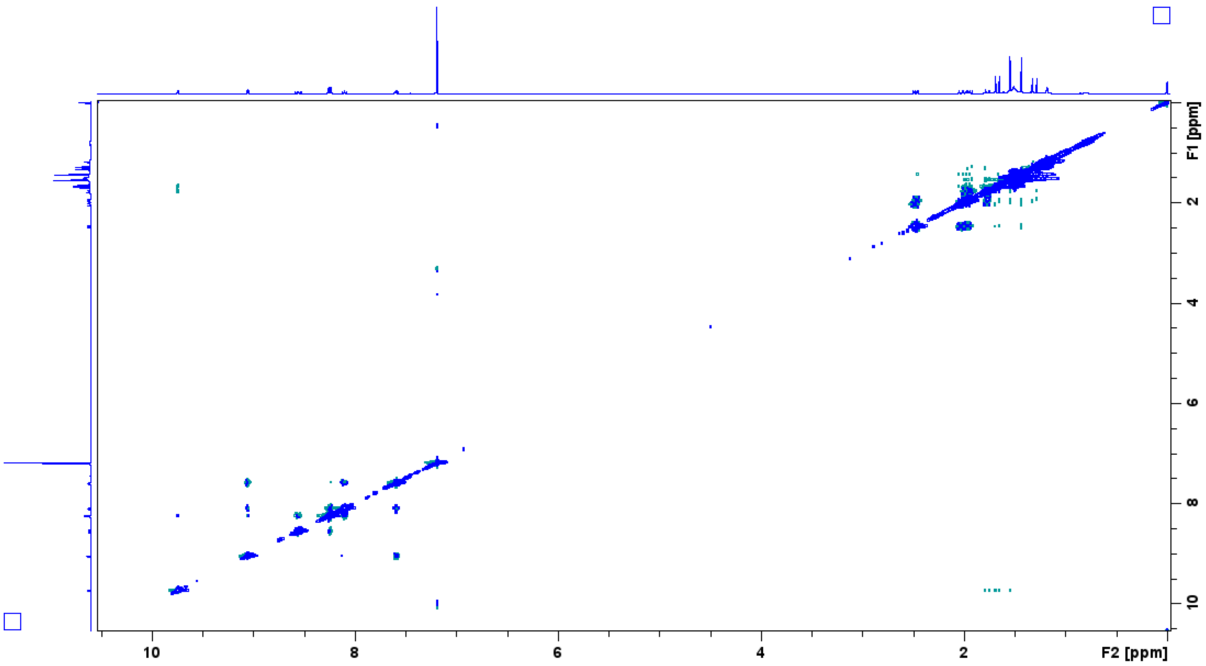

6. *Fac*-[Re(κ²-*N,N*′-Re,κ-*P*-Au-**6L**)(CO)$_3$Cl(AuCl)], **6L**^{Re,Au}.

Figure S41. ^{31}P{^{1}H} NMR spectrum of **6L**^{Re,Au} recorded at 162 MHz in CDCl$_3$.

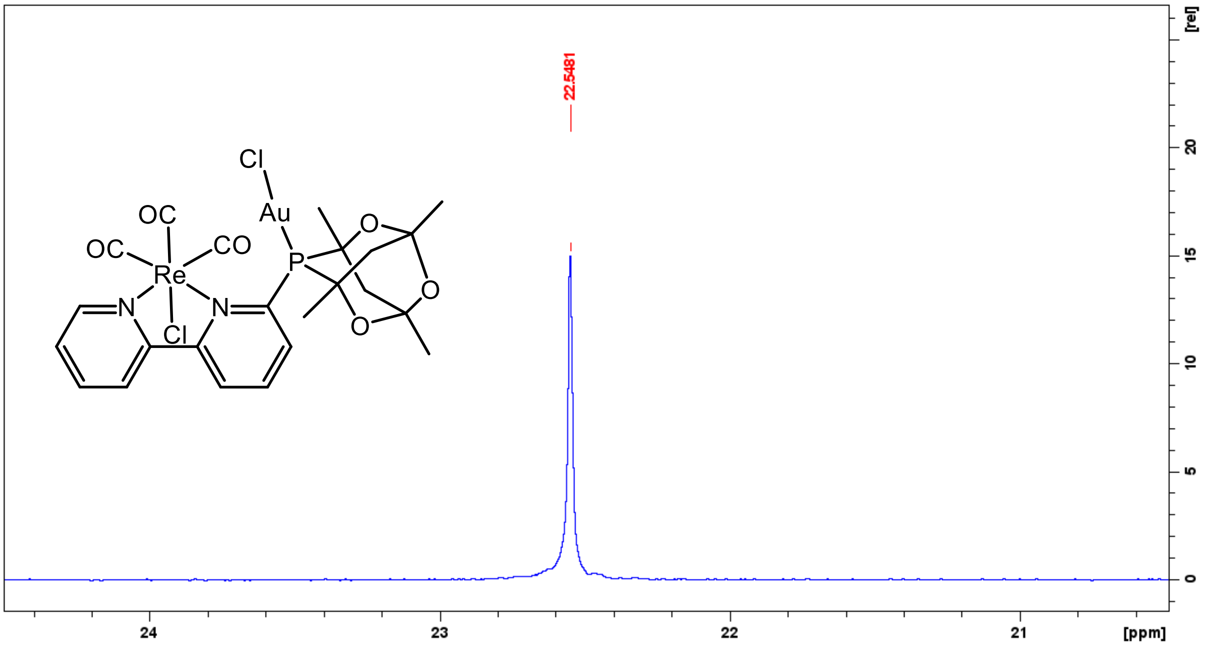

Figure S42. ^{1}H NMR spectrum of **6L**^{Re,Au} recorded at 400 MHz in d$_6$-dmso.

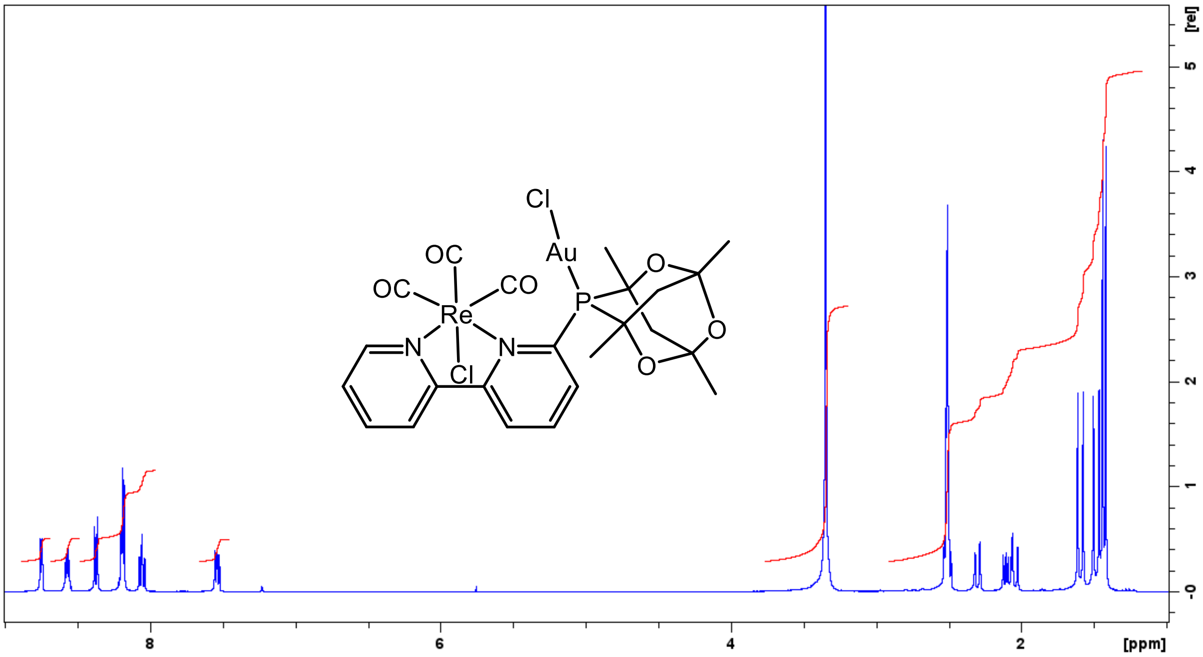

Figure S43. ^{1}H NMR spectrum of **6L^{Re,Au}** recorded at 400 MHz in $CDCl_3$.

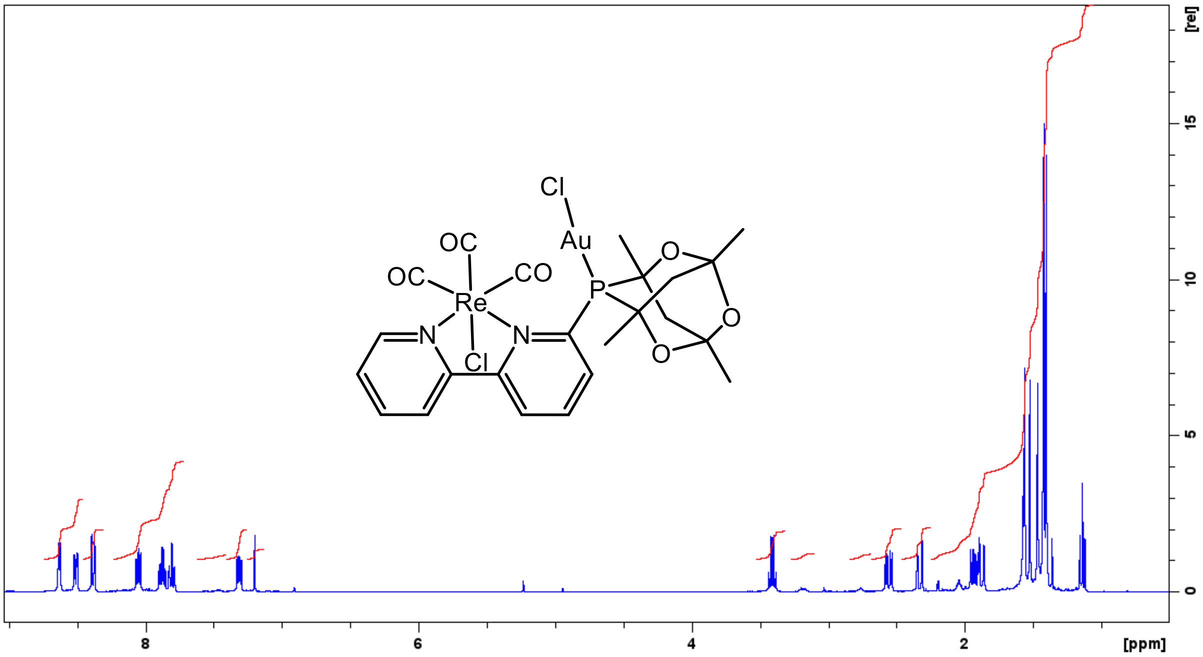

Figure S44. $^{13}C\{^{1}H\}$ DEPT NMR spectrum of **6L^{Re,Au}** recorded at 100 MHz in d_6-dmso.

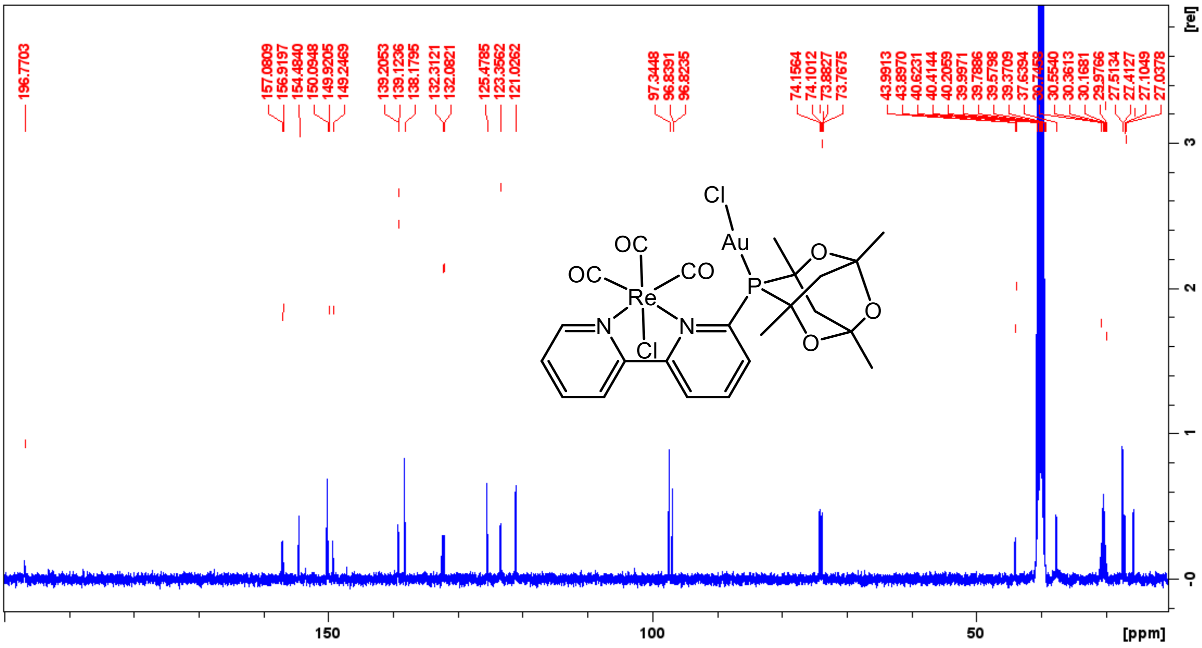

Figure S45. ^{13}C DEPT NMR spectrum of **6L^{Re,Au}** recorded at 100 MHz in d_6-dmso.

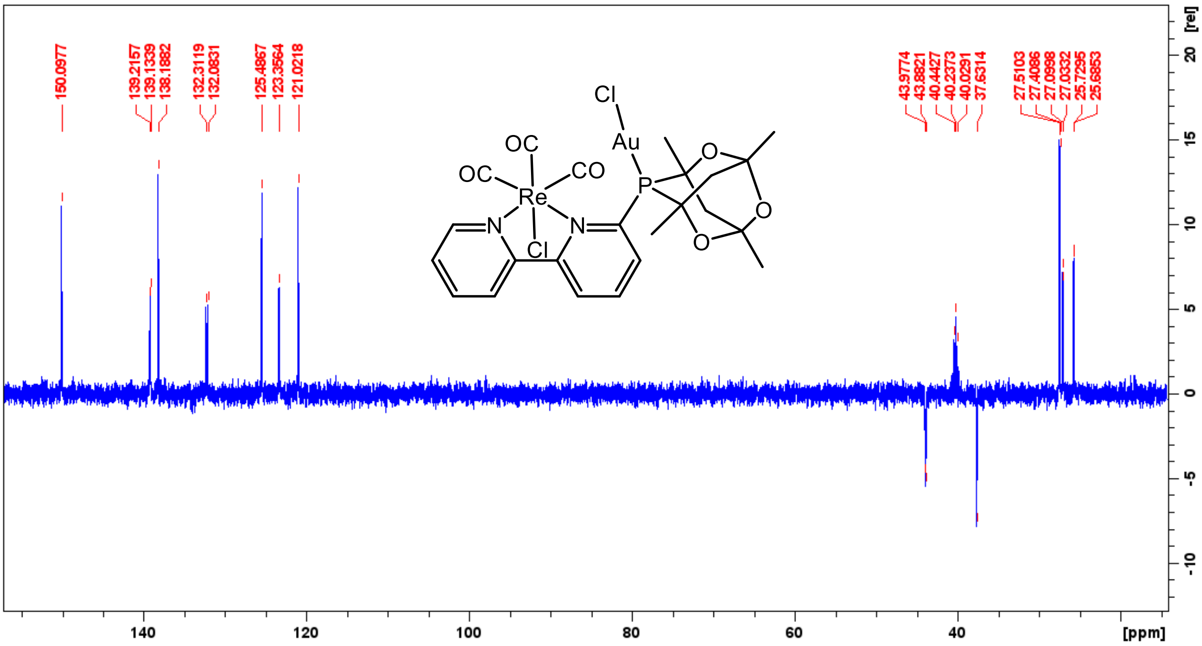

Figure S46. ^1H-^1H COSY NMR spectrum of **6L^{Re,Au}** recorded at 400 MHz in $CDCl_3$.

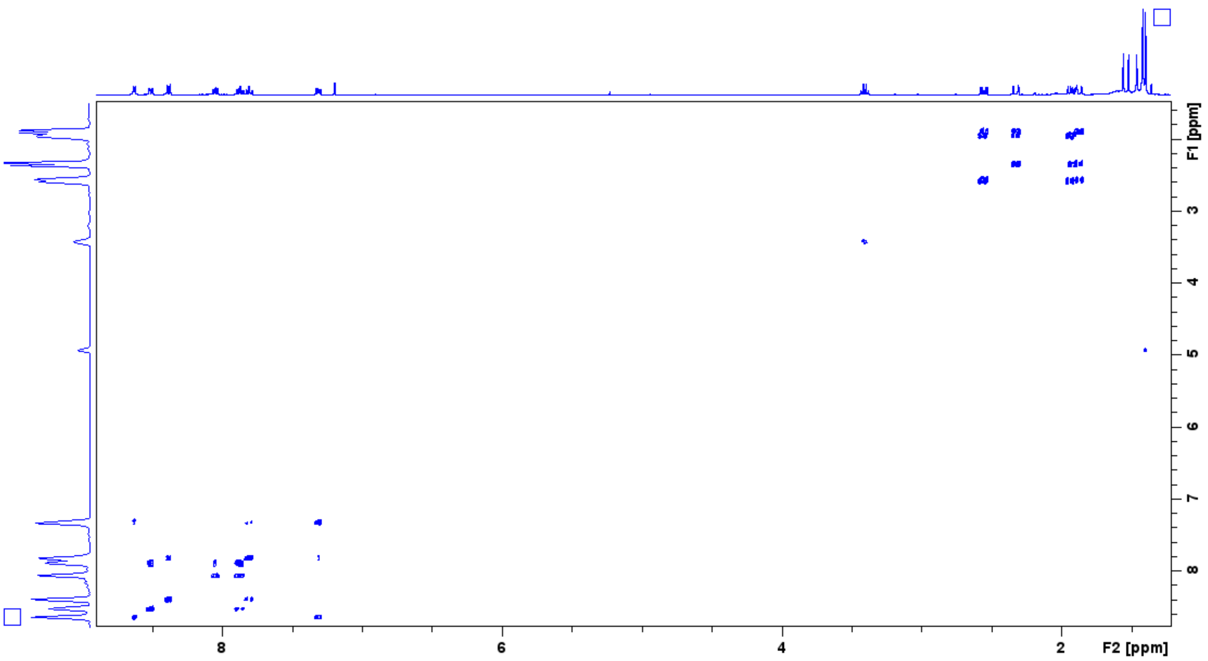

Figure S47. ^{1}H-^{13}C HSQC NMR spectrum of **6L^{Re,Au}** recorded at 400 MHz in $CDCl_3$.

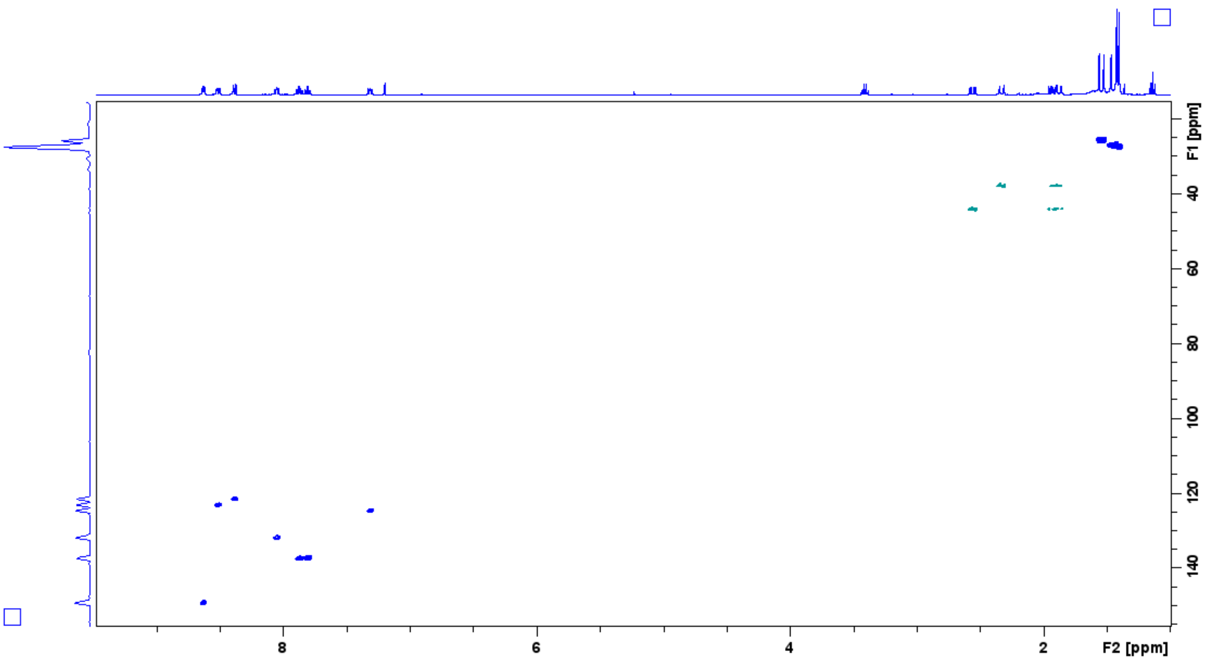

Figure S48. ^{1}H-^{1}H NOESY NMR spectrum of **6L^{Re,Au}** recorded at 400 MHz in d_6-dmso.

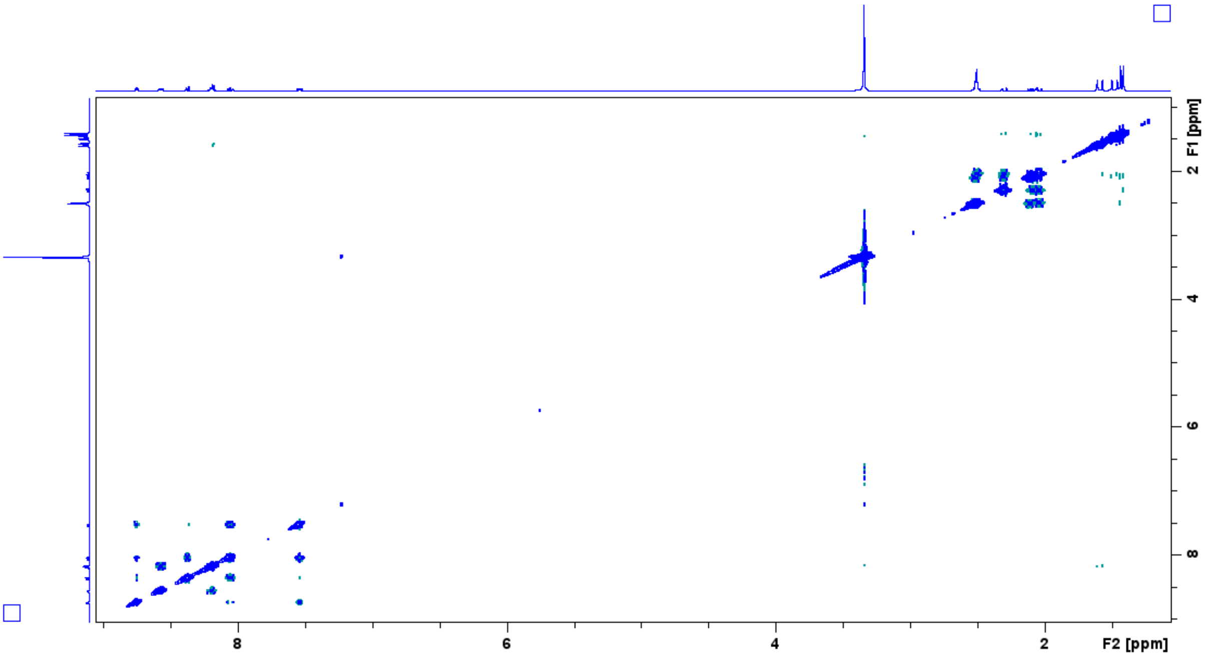

Figure S49. HRMS (ES$^+$) spectrum of **6L^{Re,Au}**.

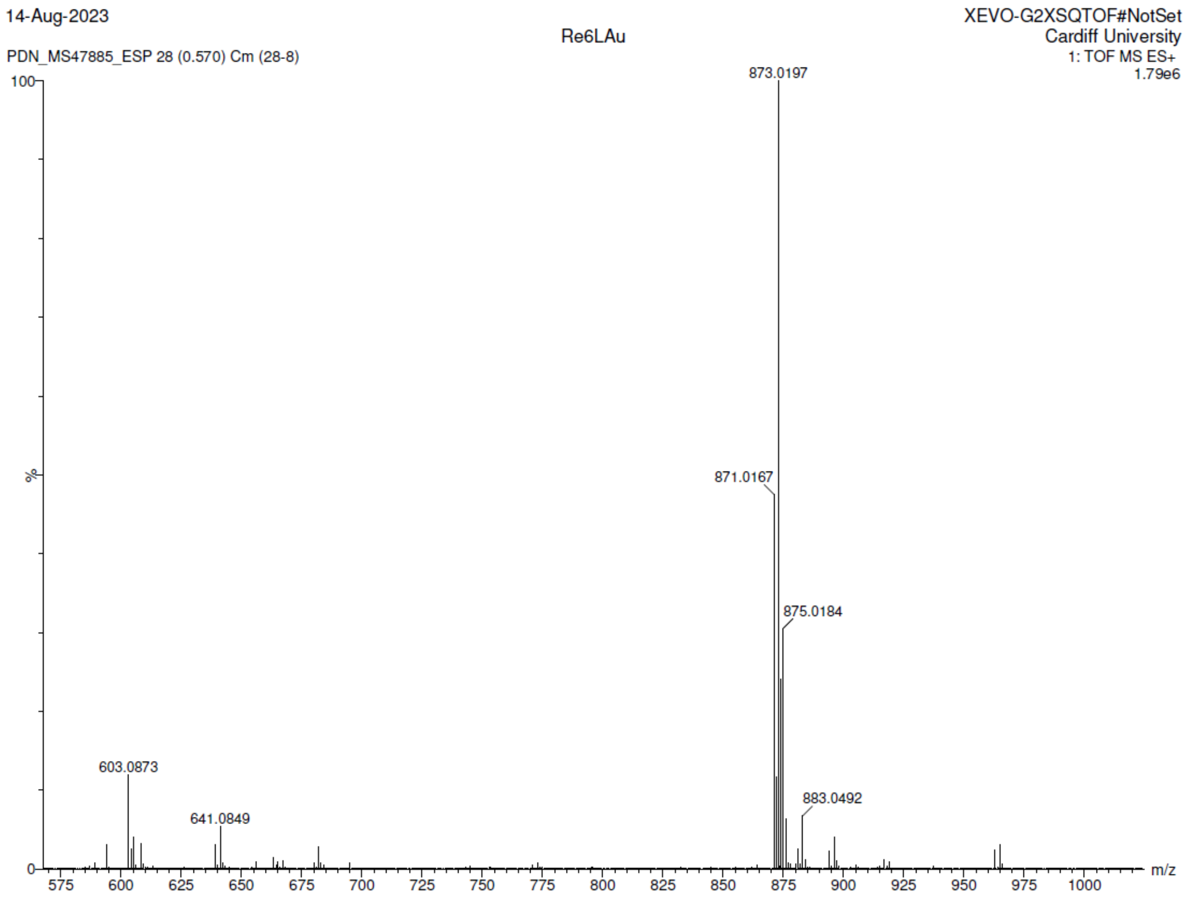

Minimum:				-1.5				
Maximum:		5000.0	10.0	50.0				
Mass	Calc. Mass	mDa	PPM	DBE	i-FIT	Norm	Conf(%)	Formula
873.0197	873.0205	-0.8	-0.9	14.0	463.9	n/a	n/a	C23 H23 N2 O6 P Cl Re Au

7. *Fac*-[{Re(κ²-*N,N'*-Re,κ-*P*-Ag-**5L**)(CO)₃Cl}₂(Ag)]OTf

Figure S50. $^{31}P\{^1H\}$ NMR spectrum of $\mathbf{5L}^{Re,Ag}$ recorded at 202 MHz in D_6-dmso.

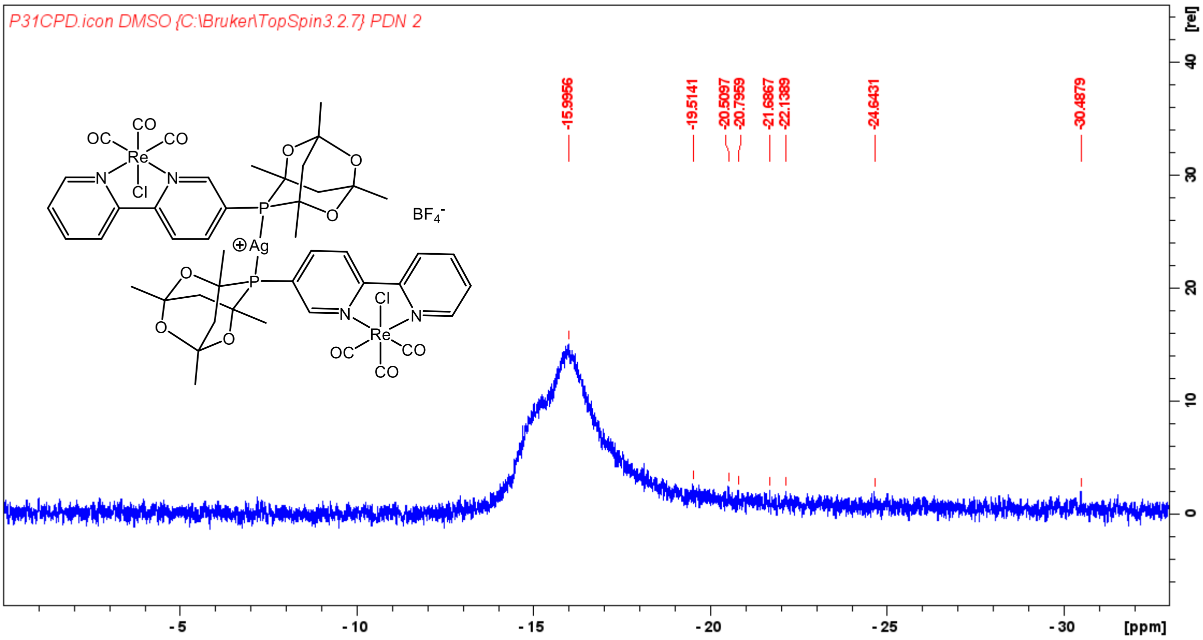

Figure S51. ^1H NMR spectrum of $\mathbf{5L}^{Re,Ag}$ recorded at 500 MHz in D_6-dmso.

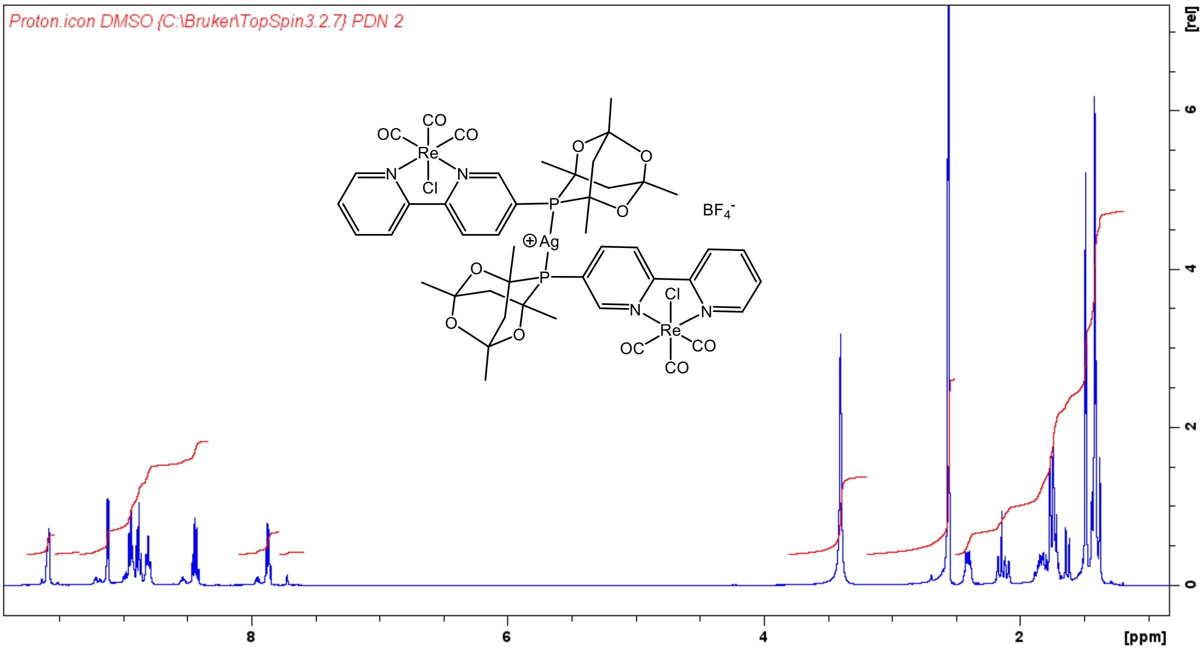

Figure S52. $^{13}C\{^{1}H\}$ NMR spectrum of **5L^{Re,Ag}** recorded at 150 MHz in D_6-dmso.

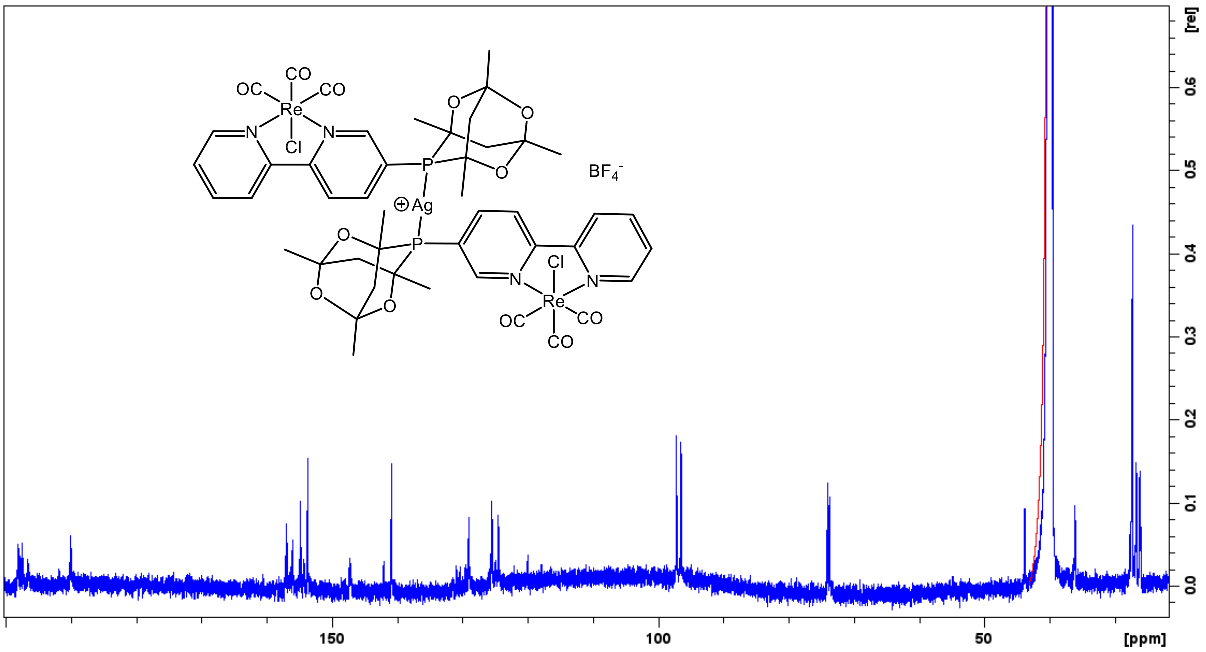

Figure S53. ^{1}H-^{1}H COSY NMR spectrum of **5L^{Re,Ag}** recorded at 500 MHz in D_6-dmso.

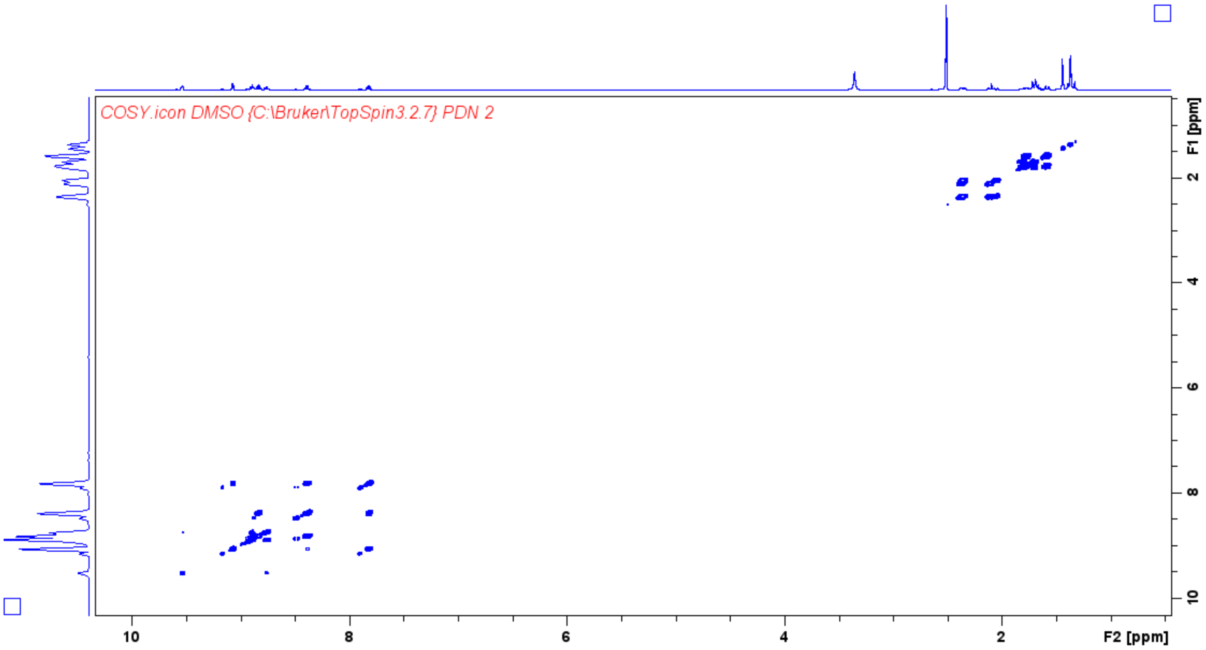

Figure S54. ^{1}H-^{13}C HSQC NMR spectrum of **5L^{Re,Ag}** recorded in D_6-dmso.

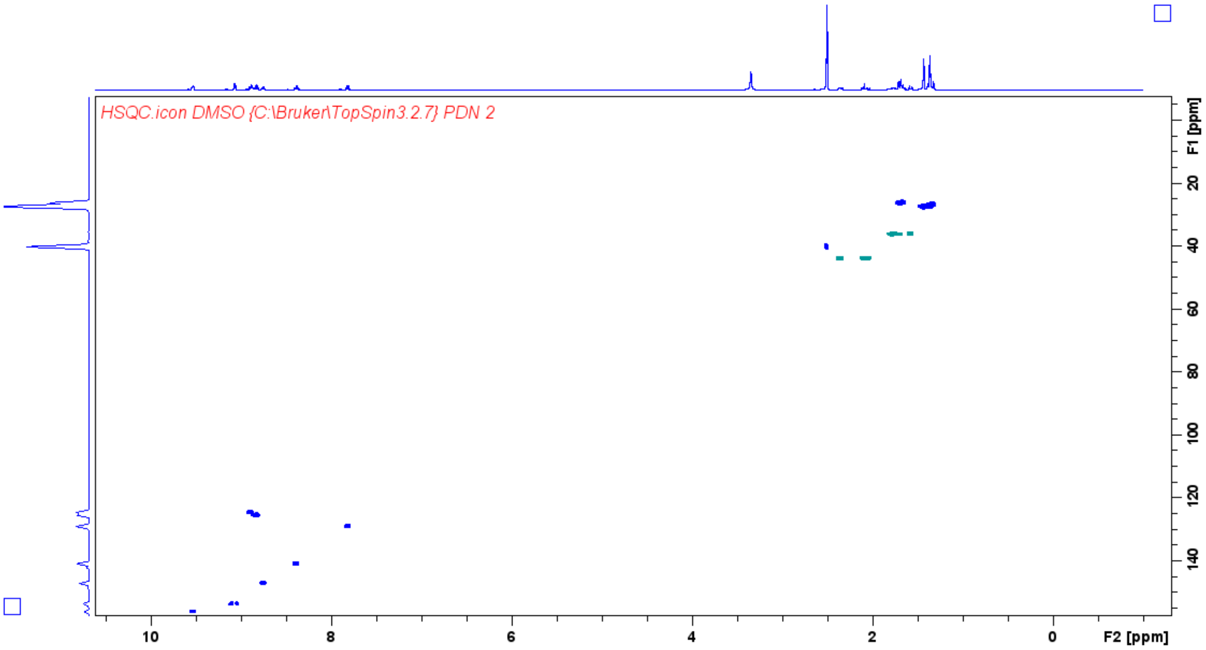

Figure S55. ^{1}H-^{1}H NOESY NMR spectrum of $\mathbf{5L^{Re,Ag}}$ recorded at 500 MHz in D_6-dmso.

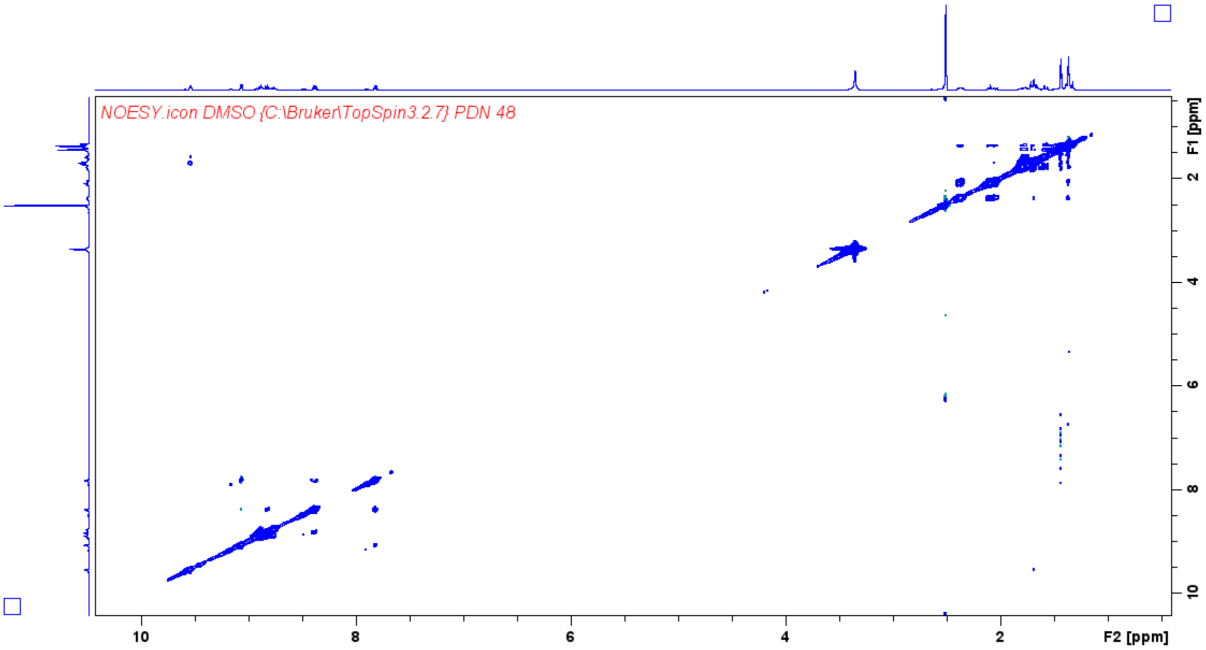

Figure S56. HRMS (ES^{+}) spectrum of $\mathbf{5L^{Re,Ag}}$.

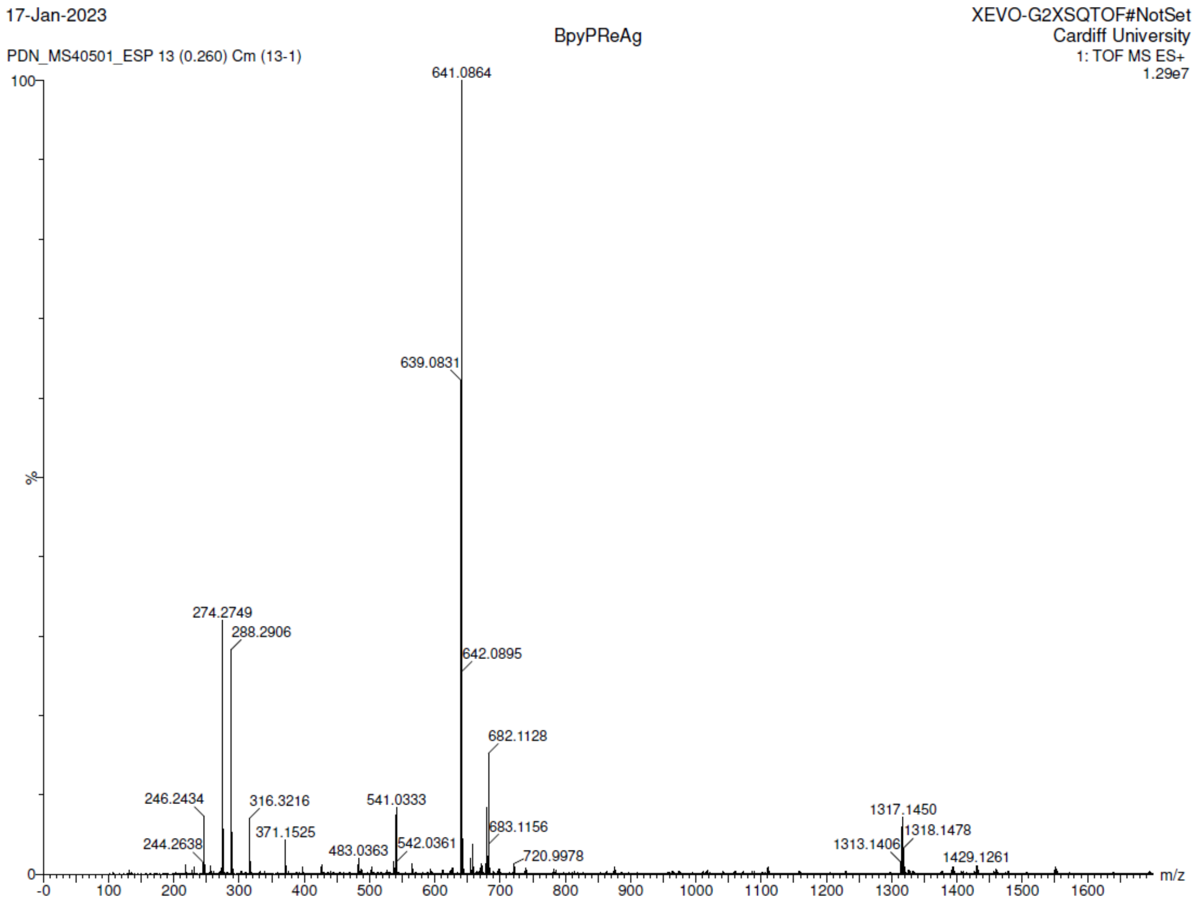
17-Jan-2023
BpyPReAg
PDN_MS40501_ESP 13 (0.260) Cm (13-1)
XEVO-G2XSQTOF#NotSet
Cardiff University
1: TOF MS ES+
1.29e7
641.0864
639.0831
274.2749
288.2906
642.0895
682.1128
246.2434
316.3216
541.0333
1317.1450
683.1156
1318.1478
244.2638
371.1525
542.0361
1313.1406
483.0363
720.9978
1429.1261
%
m/z

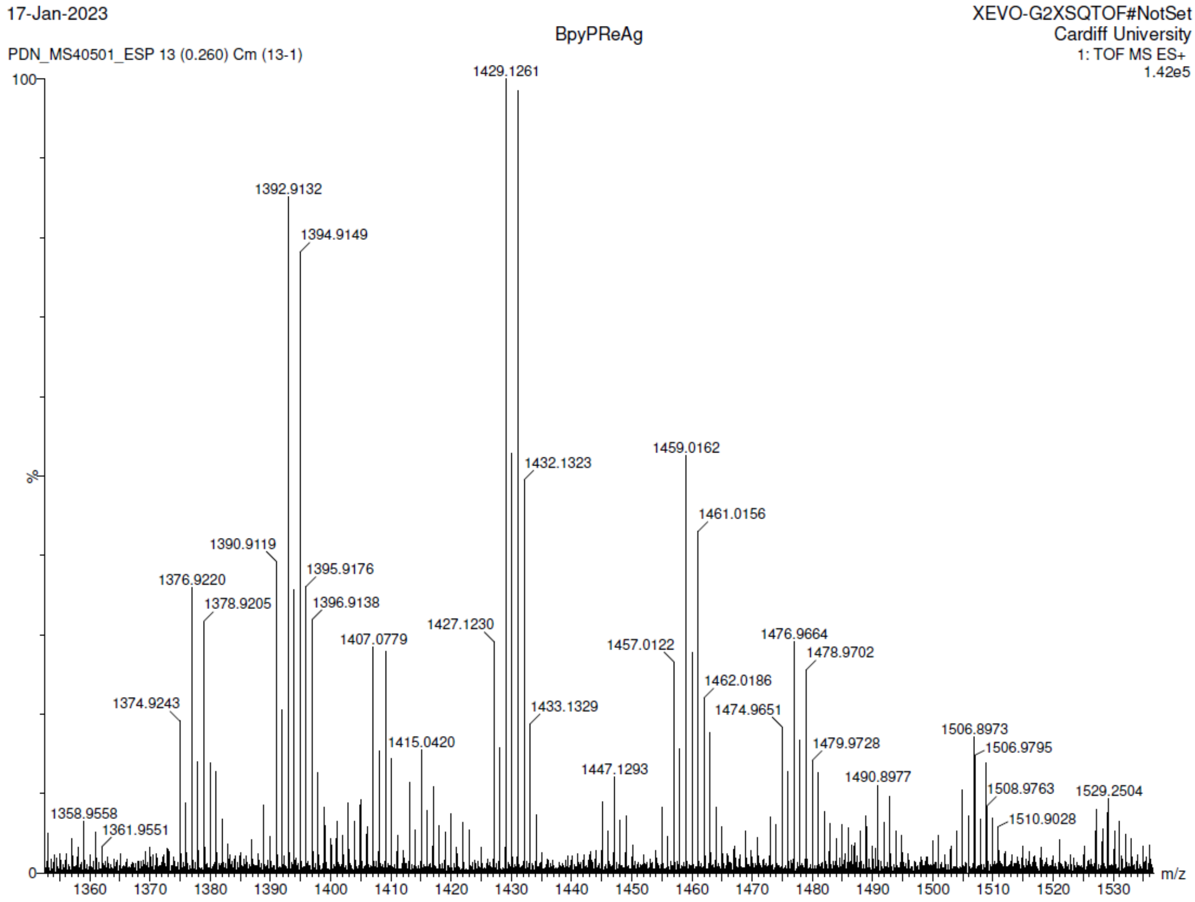
17-Jan-2023
BpyPReAg
PDN_MS40501_ESP 13 (0.260) Cm (13-1)
XEVO-G2XSQTOF#NotSet
Cardiff University
1: TOF MS ES+
1.42e5
1429.1261
1392.9132
1394.9149
1459.0162
1432.1323
1461.0156
1390.9119
1395.9176
1376.9220
1378.9205
1396.9138
1427.1230
1476.9664
1407.0779
1457.0122
1478.9702
1462.0186
1474.9651
1374.9243
1433.1329
1415.0420
1479.9728
1506.8973
1506.9795
1447.1293
1490.8977
1508.9763
1529.2504
1358.9558
1510.9028
1361.9551
%
m/z

	Mass	Calc. Mass	mDa	PPM	DBE	i-FIT	Norm	Conf(%)	Formula
Minimum:					-1.5				
Maximum:			5.0	20.0	100.0				
	1459.0162	1459.0130	3.2	2.2	25.5	493.4	n/a	n/a	C46 H46 N4 O12 P2 Cl2 Ag Re2

8. [Au(κ-*P*-**6L**)Cl]

Figure S57. $^{31}P\{^{1}H\}$ NMR spectrum of [Au(κ-*P*-**6L**)Cl] recorded at 162 MHz in $CDCl_3$.

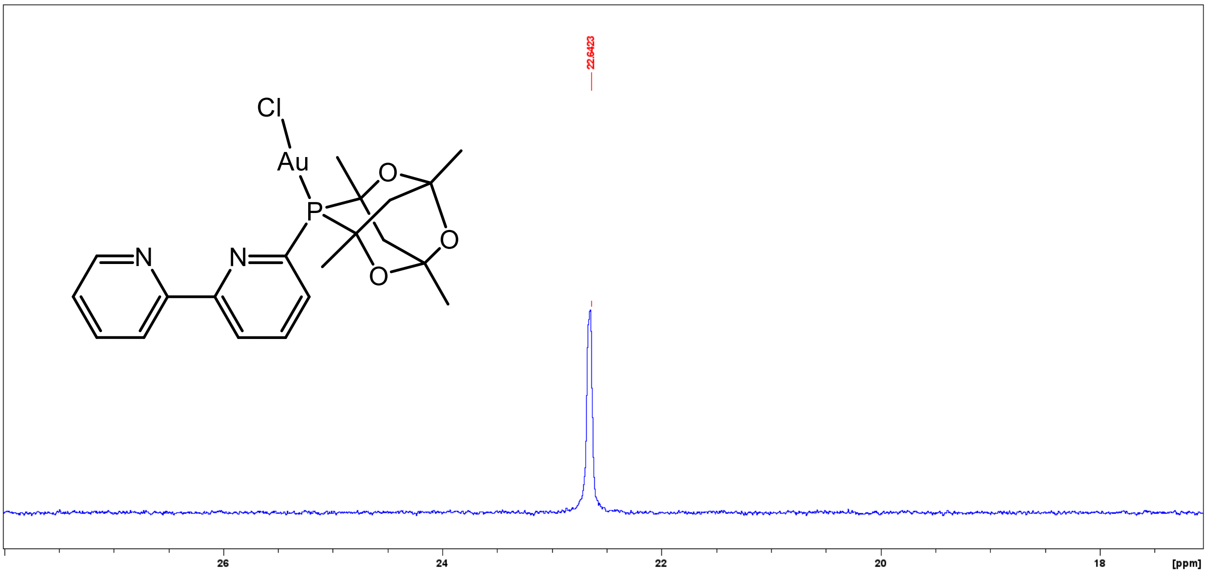

Figure S58. ^{1}H NMR spectrum of [Au(κ-*P*-**6L**)Cl] recorded at 400 MHz in $CDCl_3$.

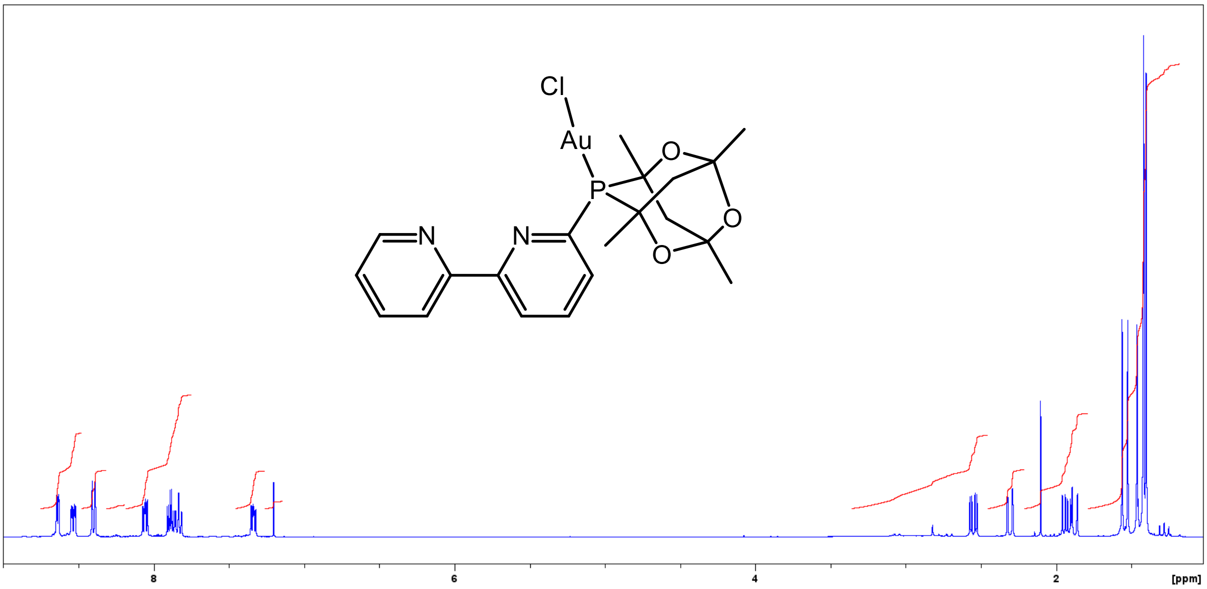

Figure S59. $^{13}C\{^{1}H\}$ NMR spectrum of [Au(κ-*P*-**6L**)Cl] recorded at 100 MHz in $CDCl_3$.

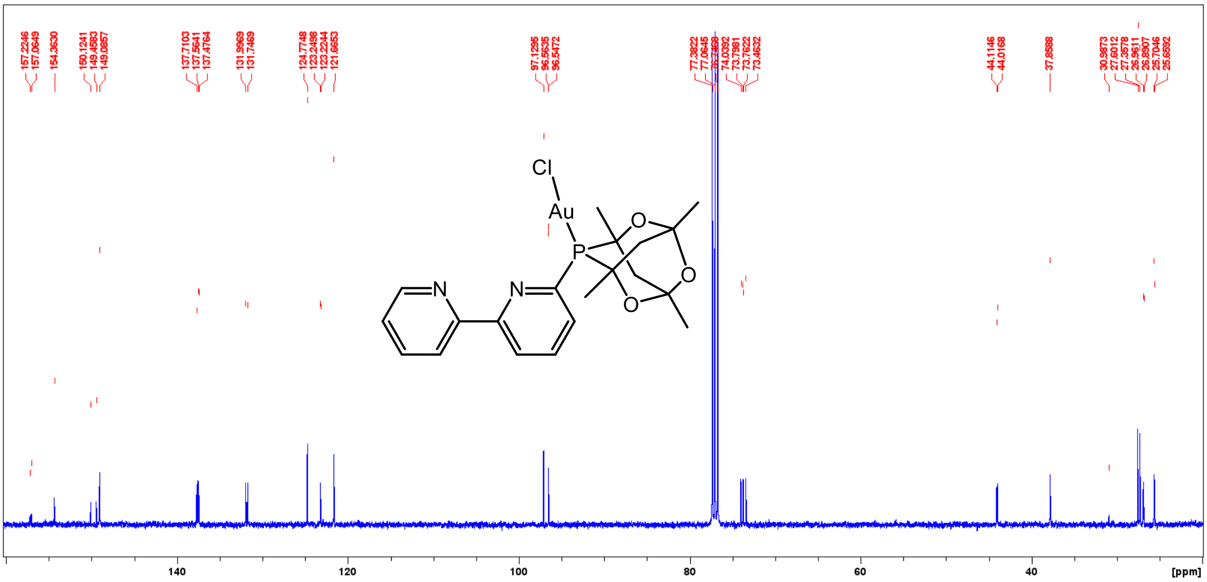

Figure S60. ^{1}H-^{13}C HSQC NMR spectrum of [Au(κ-*P*-**6L**)Cl] recorded in $CDCl_3$.

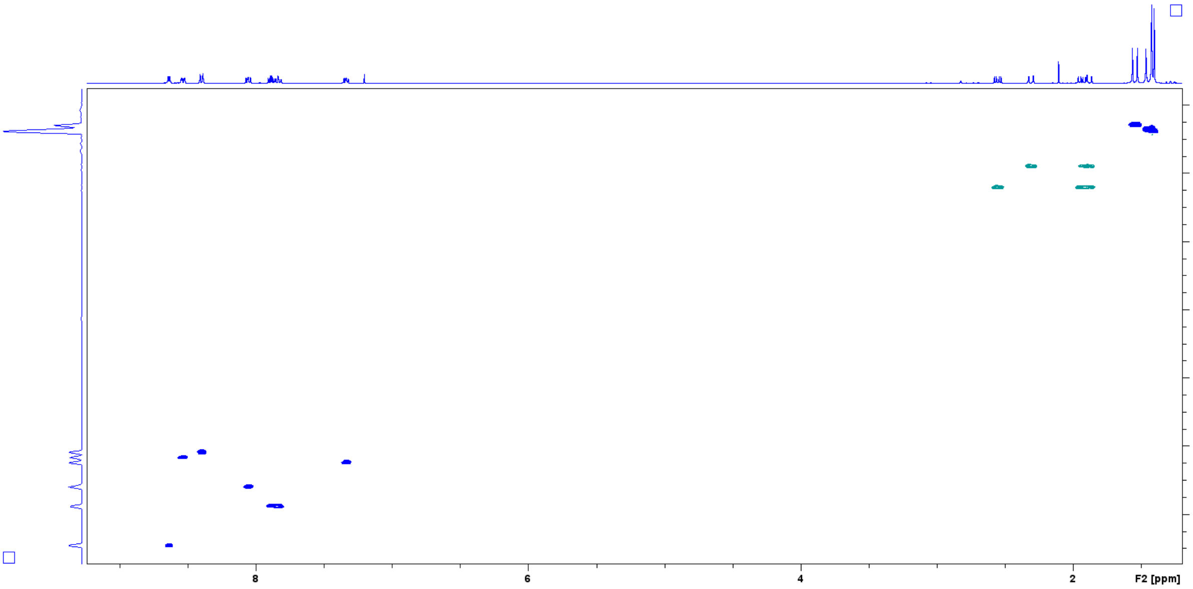

Figure S61. HRMS spectrum of [Au(κ-*P*-**6L**)Cl].

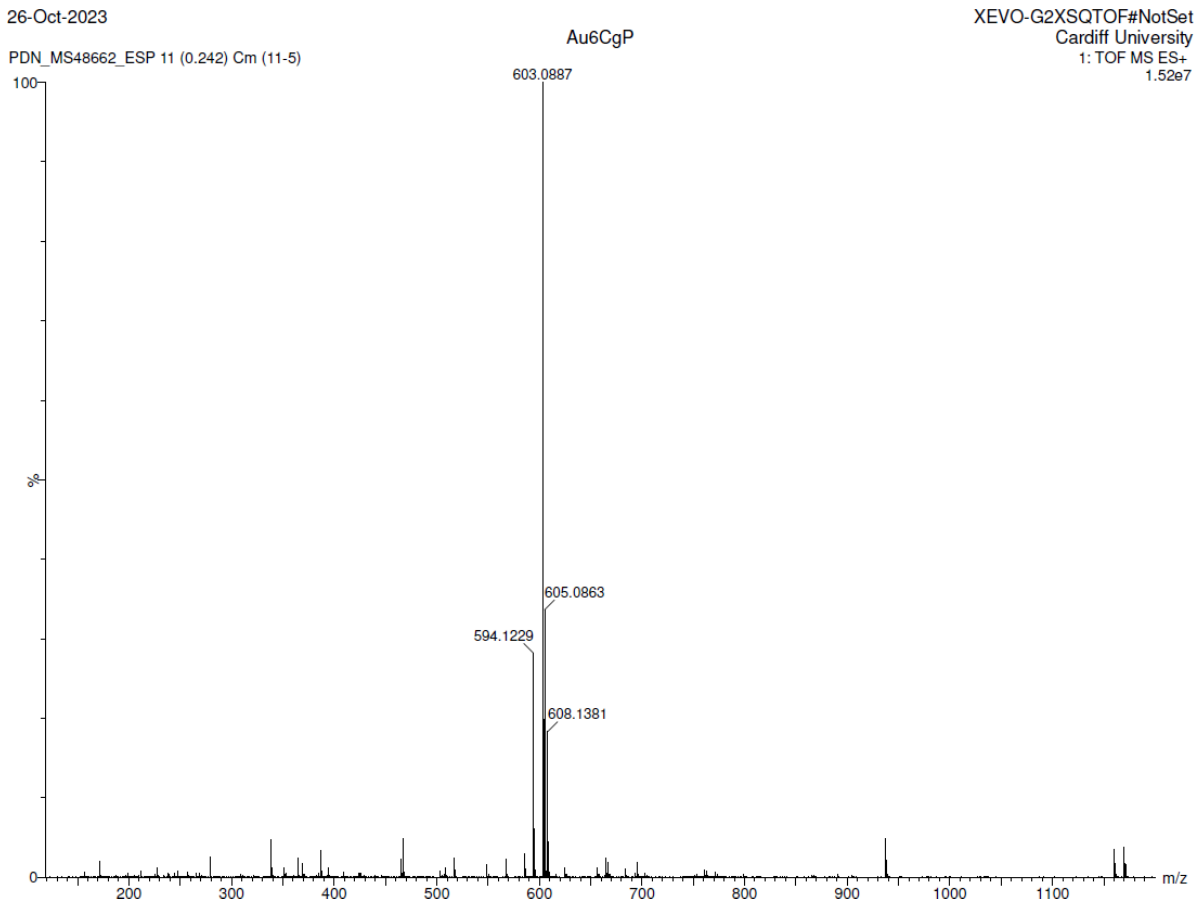

Minimum:				-1.5				
Maximum:		5.0	10.0	50.0				
Mass	Calc. Mass	mDa	PPM	DBE	i-FIT	Norm	Conf(%)	Formula
603.0887	603.0879	0.8	1.3	10.5	1203.2	n/a	n/a	C20 H24 N2 O3 P Cl Au

9. [Ag(κ-*P*-**6L**)$_2$]BF_4

Figure S62. $^{31}P\{^1H\}$ NMR spectrum of [Ag(κ-*P*-**6L**)$_2$]BF_4 recorded at 162 MHz in $CDCl_3$.

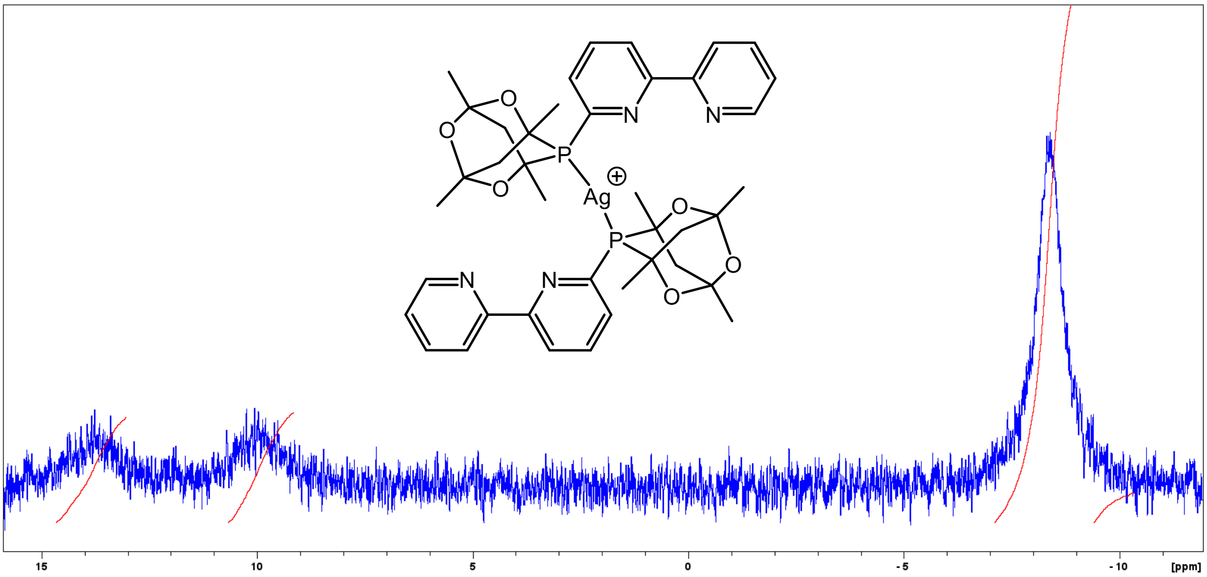

Figure S63. ^{1}H NMR spectrum of [Ag(κ-*P*-**6L**)$_2$]BF_4 recorded at 400 MHz in $CDCl_3$.

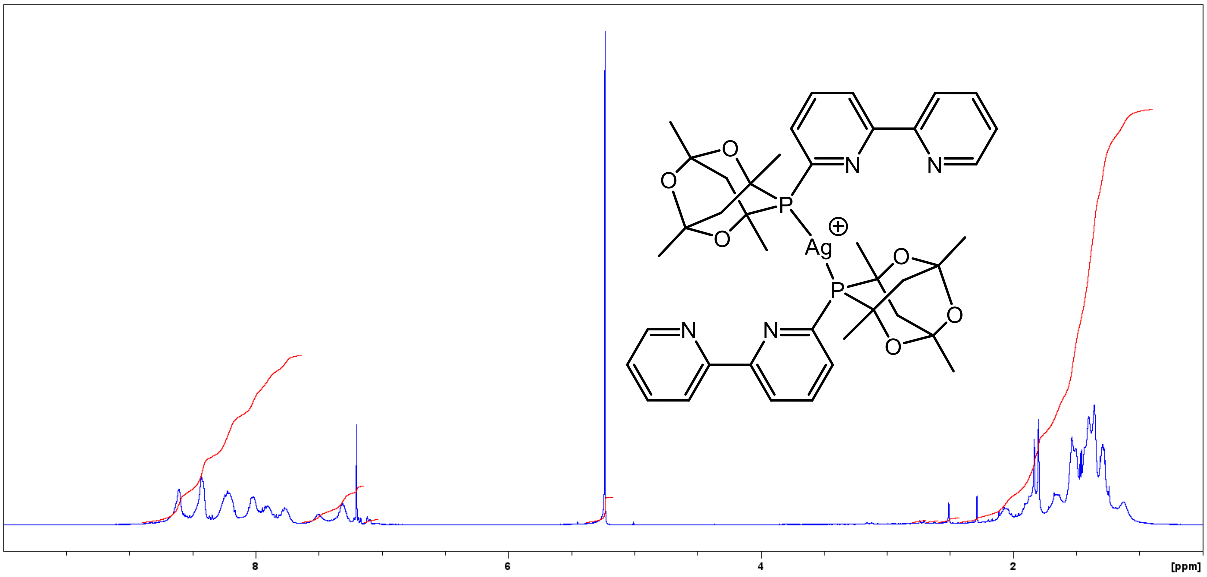

10. *Fac*-[Re(κ^2-*N,N'*-Re,κ-*P*-Rh-**5L**)(CO)$_3$Cl(Rh(COD)Cl)]

Figure S64. ^{31}P{^{1}H} NMR spectrum of **5L^{Re,Rh}** recorded at 202 MHz in D_6-acetone.

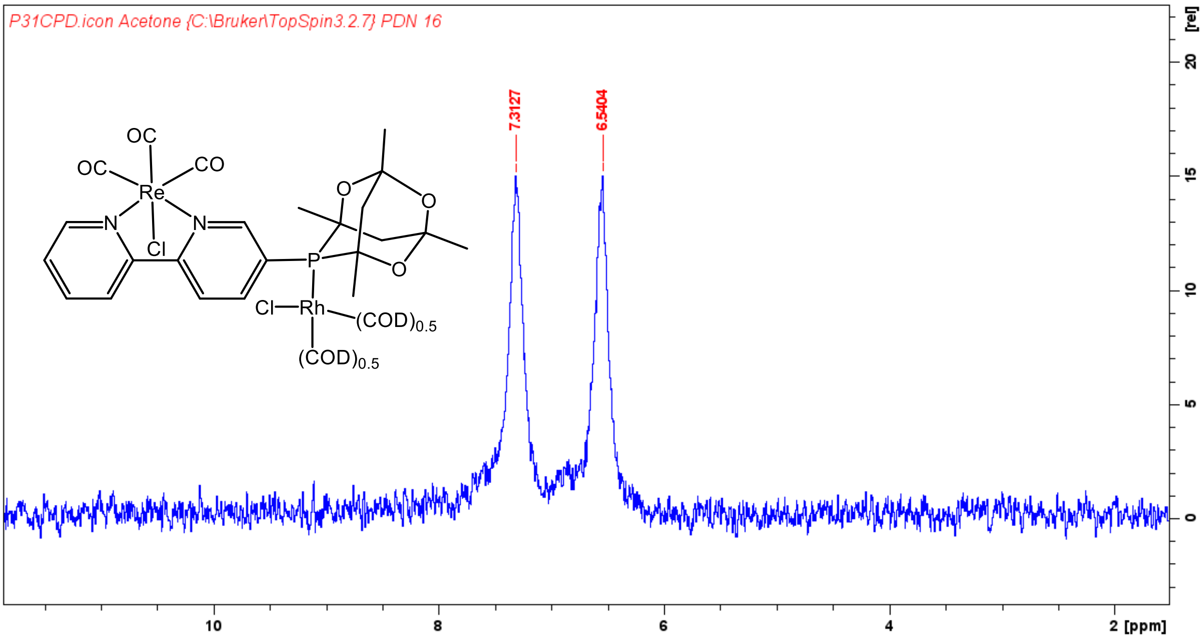

Figure S65. ^{1}H NMR spectrum of **5L^{Re,Rh}** recorded at 500 MHz in D_6-acetone (a singlet at 2.3 ppm and multiplets between 7.2 and 7.6 ppm are assigned to residual toluene).

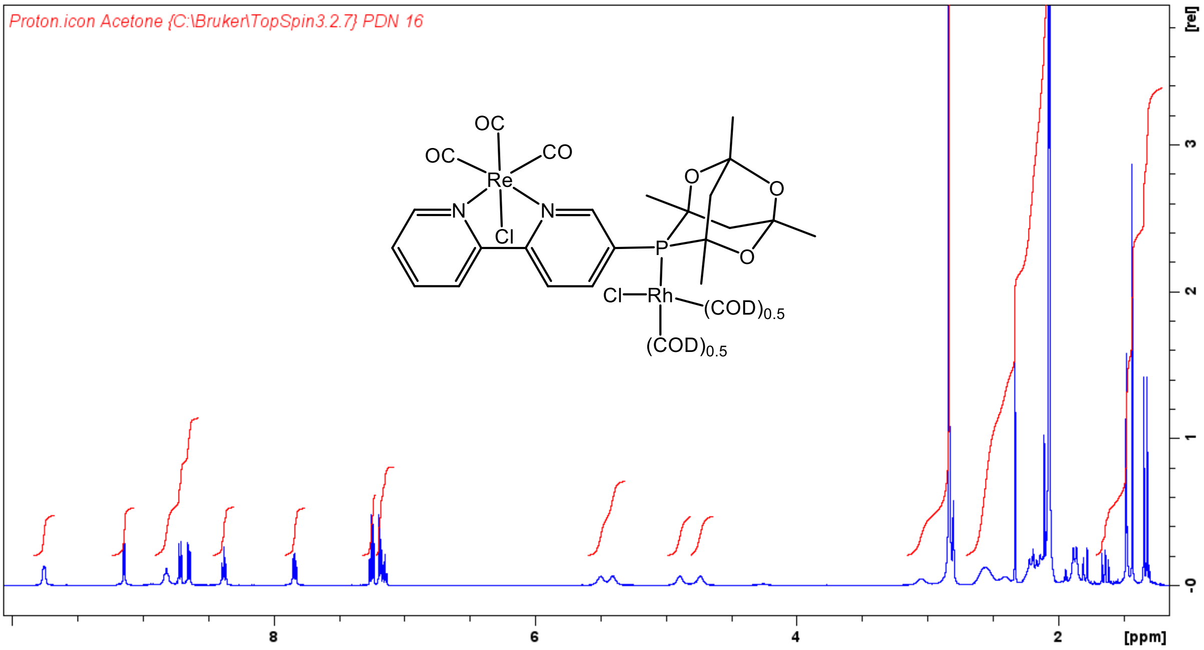

Figure S66. $^{13}C\{^{1}H\}$ NMR spectrum of **5L^{Re,Rh}** recorded at 125 MHz in $CDCl_3$.

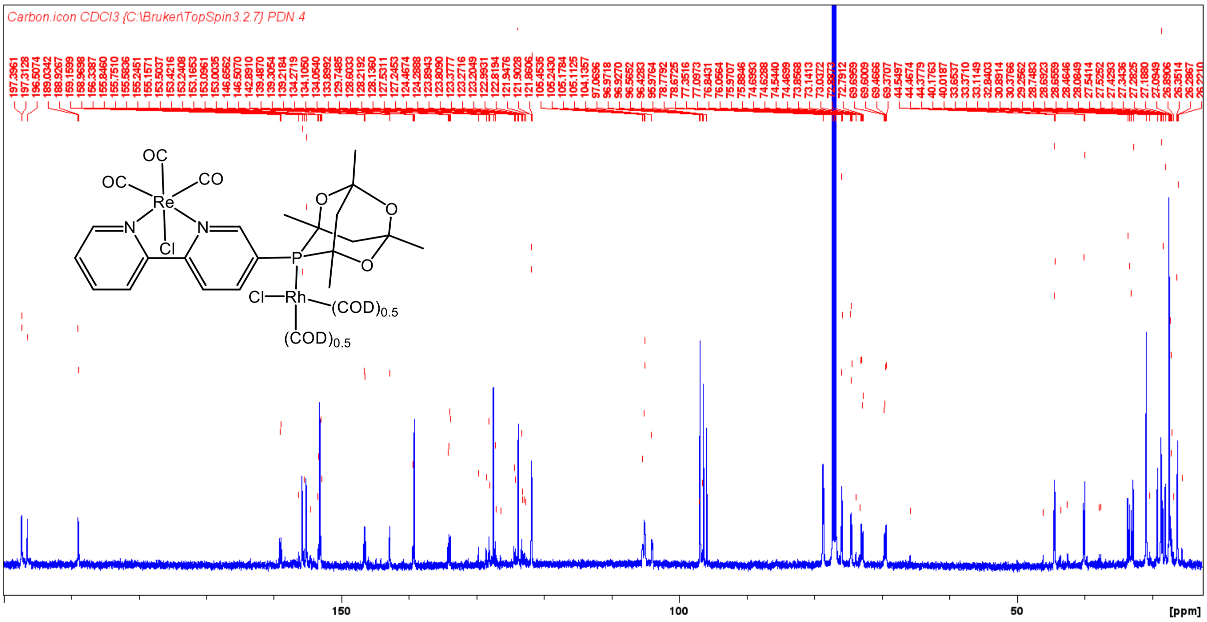

Figure S67. ^{13}C DEPT NMR spectrum of **5L^{Re,Rh}** recorded at 125 MHz in $CDCl_3$.

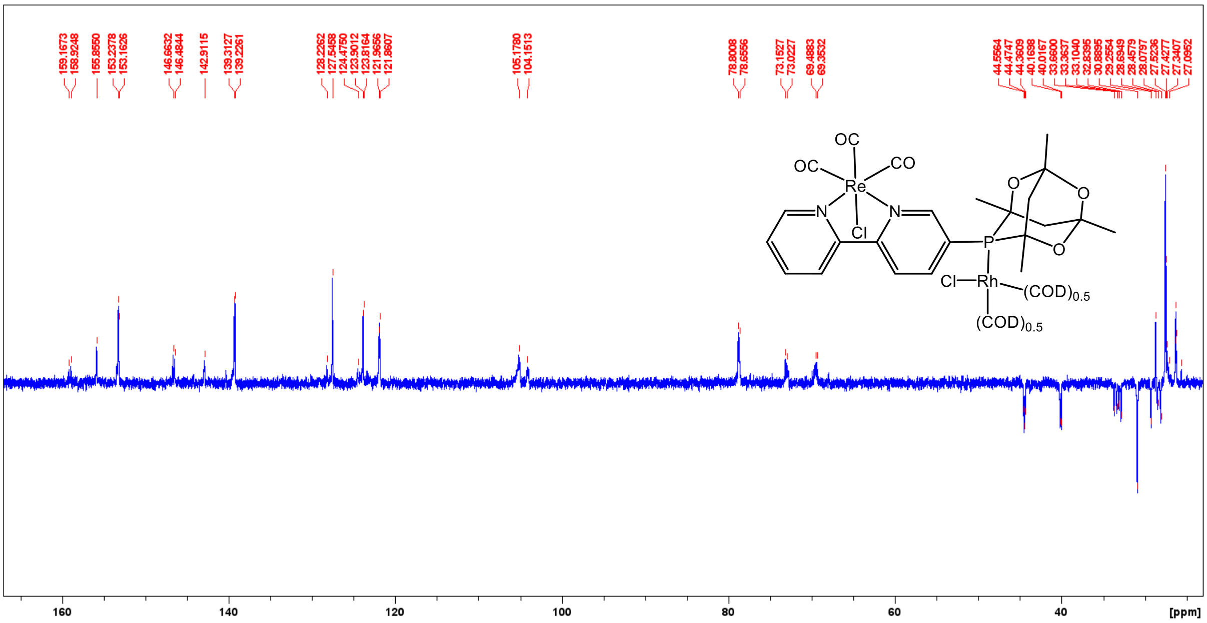

Figure S68. ^{1}H-^{13}C HSQC NMR spectrum of **5L^{Re,Rh}** recorded in D_6-acetone.

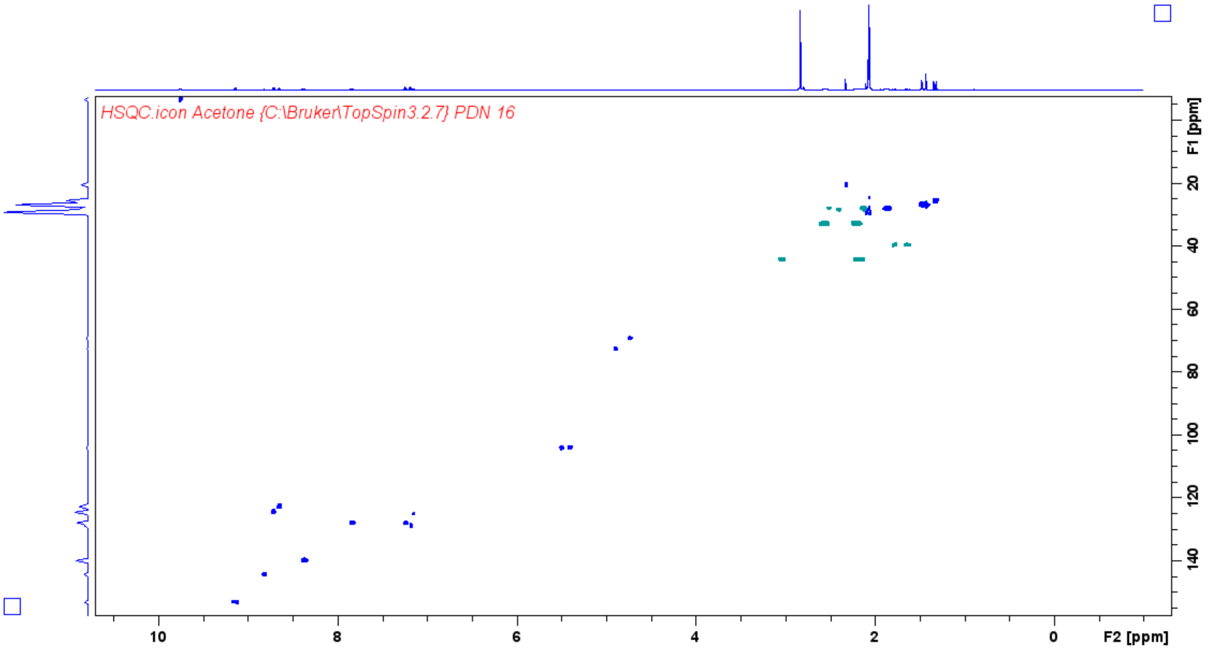

Figure S69. ^1H-^1H NMR COSY spectrum of **5L^{Re,Rh}** recorded at 500 MHz in D_6-acetone.

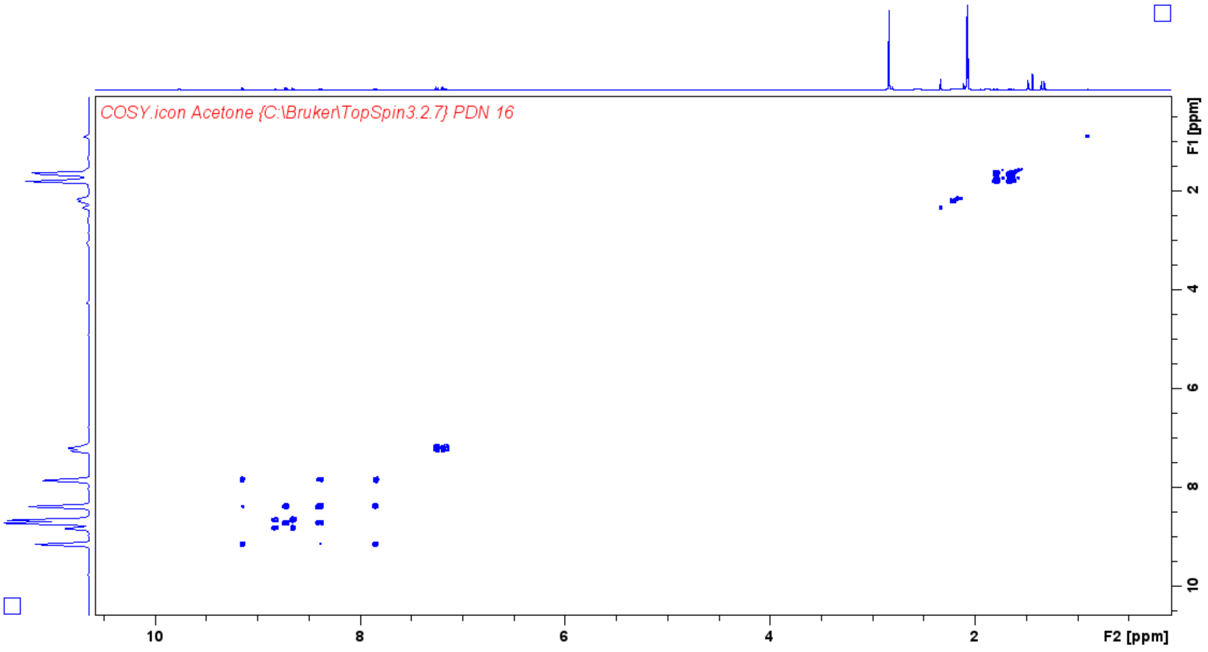

Figure S70. ^1H-^1H NOESY NMR spectrum of **5L^{Re,Rh}** recorded at 500 MHz in D_6-acetone.

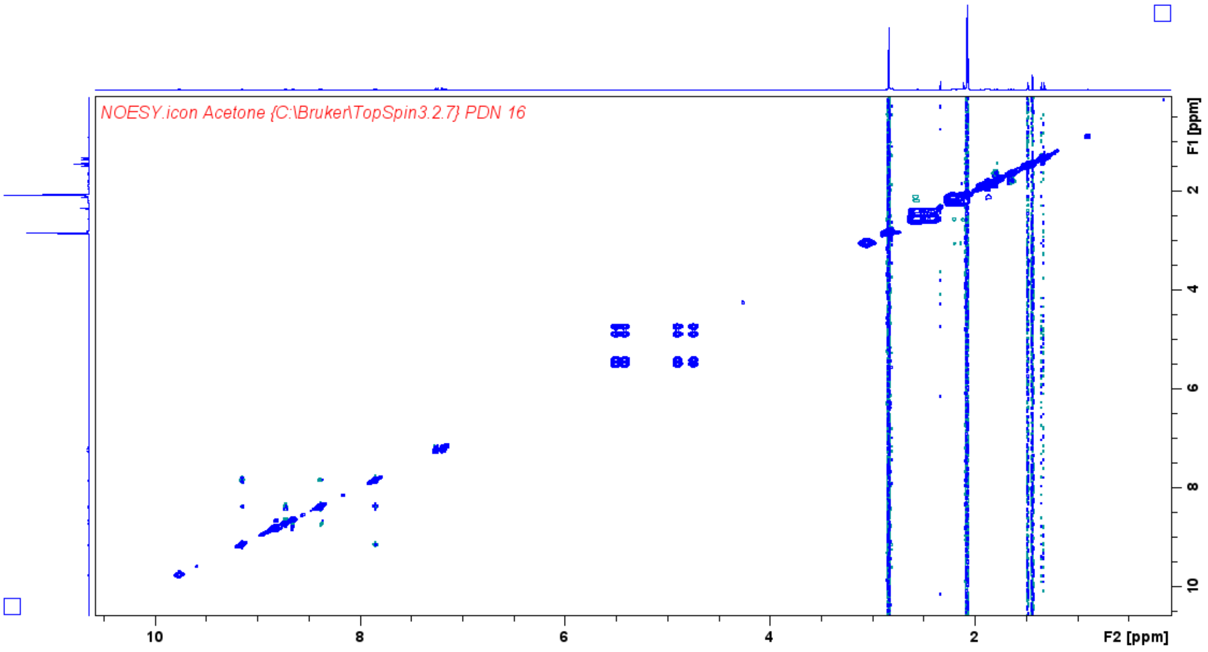

Figure S71. HRMS spectrum of **5L**$^{\mathbf{Re,Rh}}$.

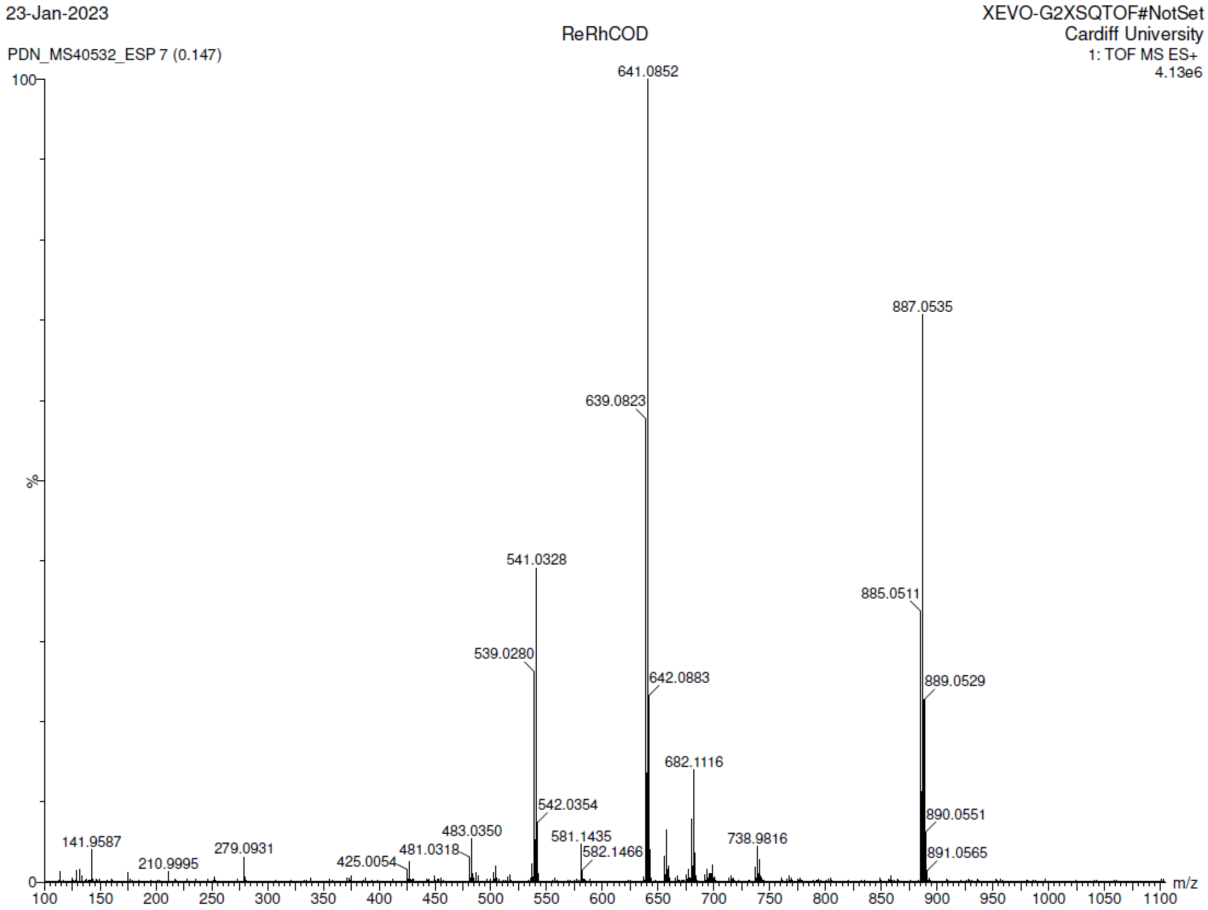

Minimum:				-1.5				
Maximum:		5.0	10.0	100.0				
Mass	Calc. Mass	mDa	PPM	DBE	i-FIT	Norm	Conf(%)	Formula
887.0535	887.0534	0.1	0.1	15.5	691.0	n/a	n/a	C31 H35 N2 O6 P 103Rh 187Re 35Cl

11. [Rh(κ-*P*-**6L**)(COD)Cl)]

Figure S72. $^{31}P\{^1H\}$ NMR spectrum of [Rh(κ-*P*-**6L**)(COD)Cl)] recorded at 162 MHz in $CDCl_3$.

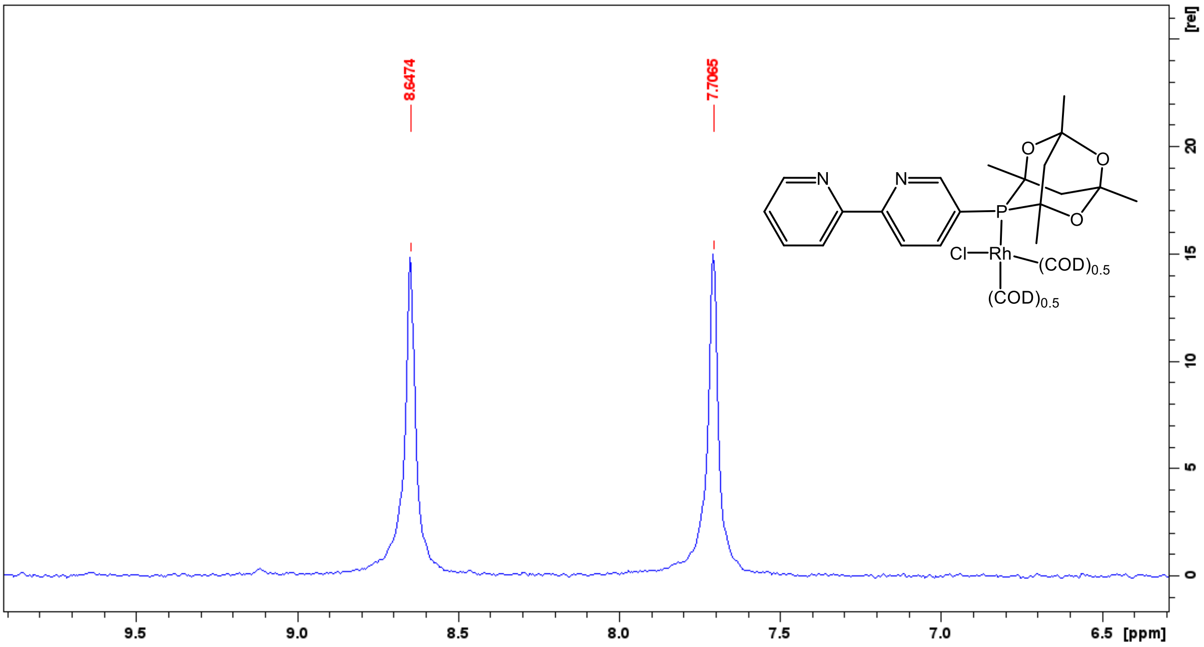

Figure S73. ^1H NMR spectrum of [Rh(κ-*P*-**6L**)(COD)Cl)] recorded at 400 MHz in $CDCl_3$.

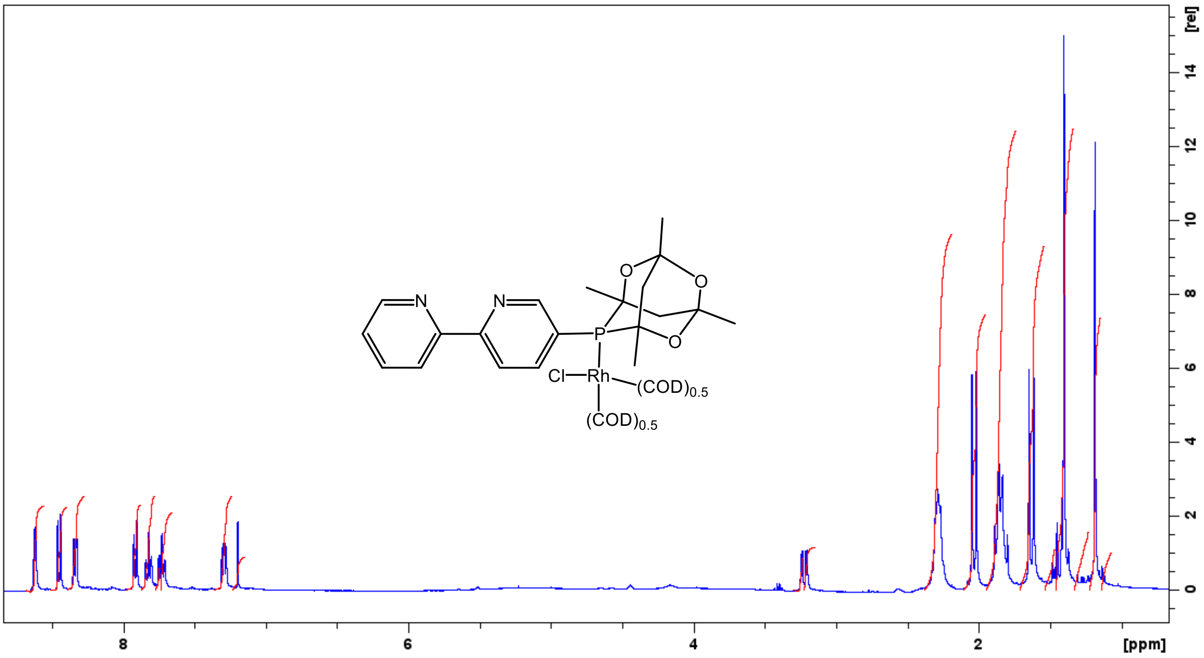

Figure S74. ^{1}H-^{1}H COSY NMR spectrum of [Rh(κ-*P*-**6L**)(COD)Cl)] recorded at 400 MHz in $CDCl_3$.

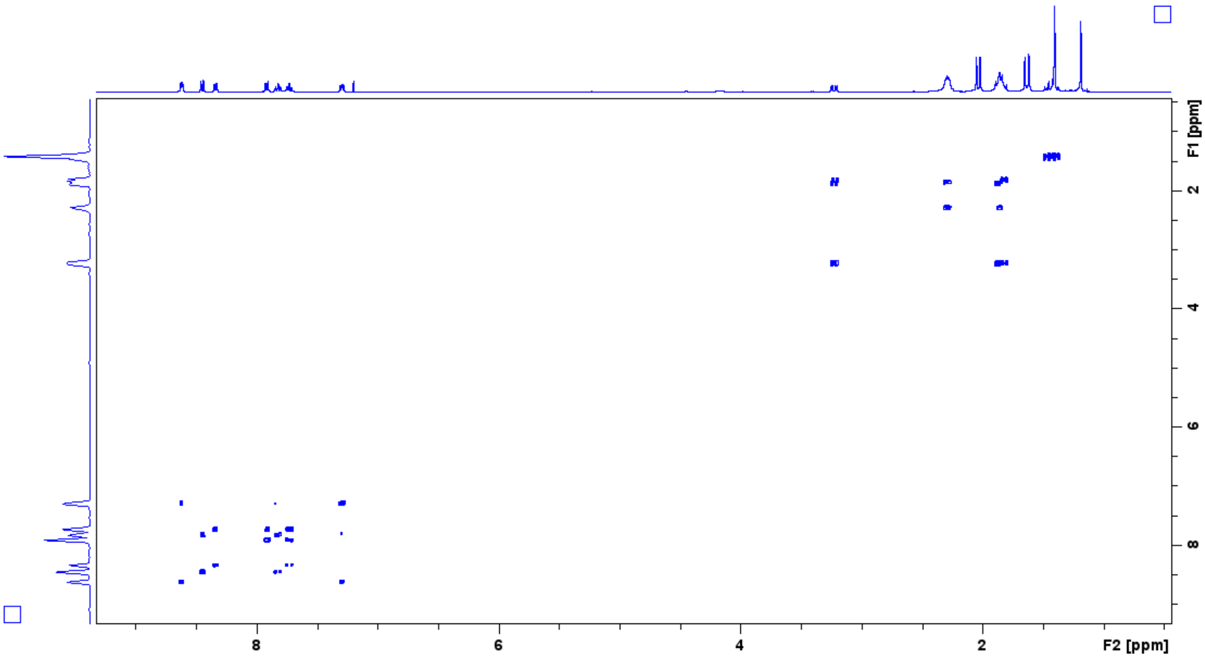

Figure S75. ^{1}H-^{13}C HSQC NMR spectrum of [Rh(κ-*P*-**6L**)(COD)Cl)] recorded in $CDCl_3$.

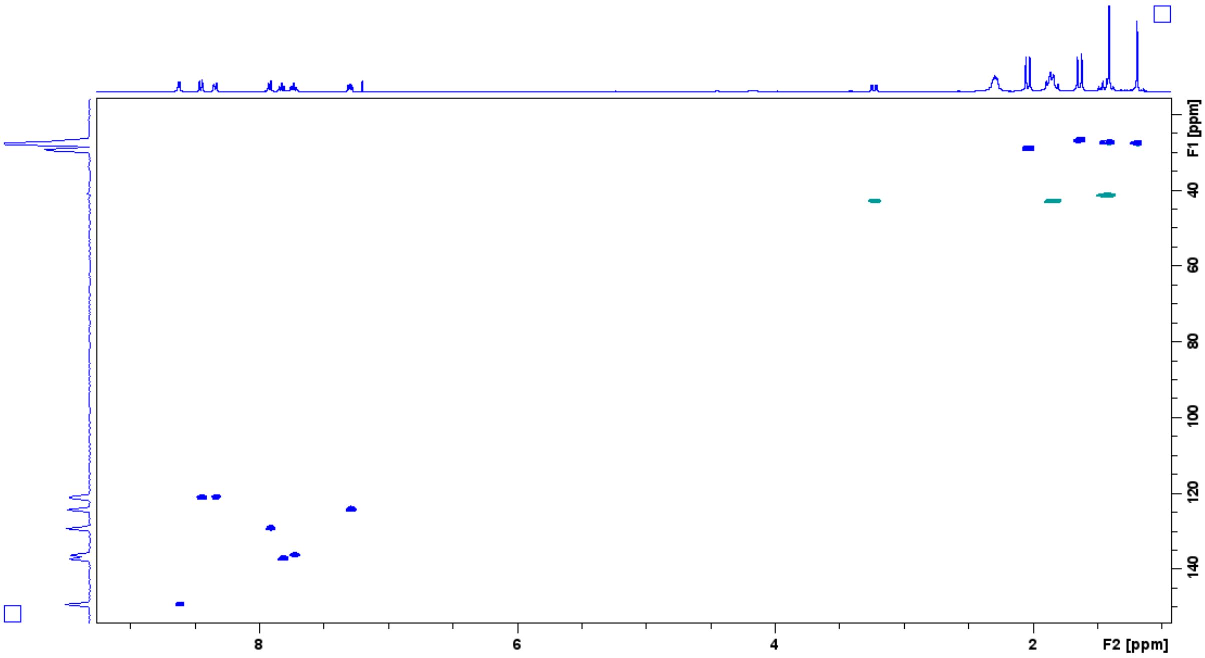

Figure S76. HRMS spectrum of [Rh(κ-*P*-**6L**)(COD)Cl)].

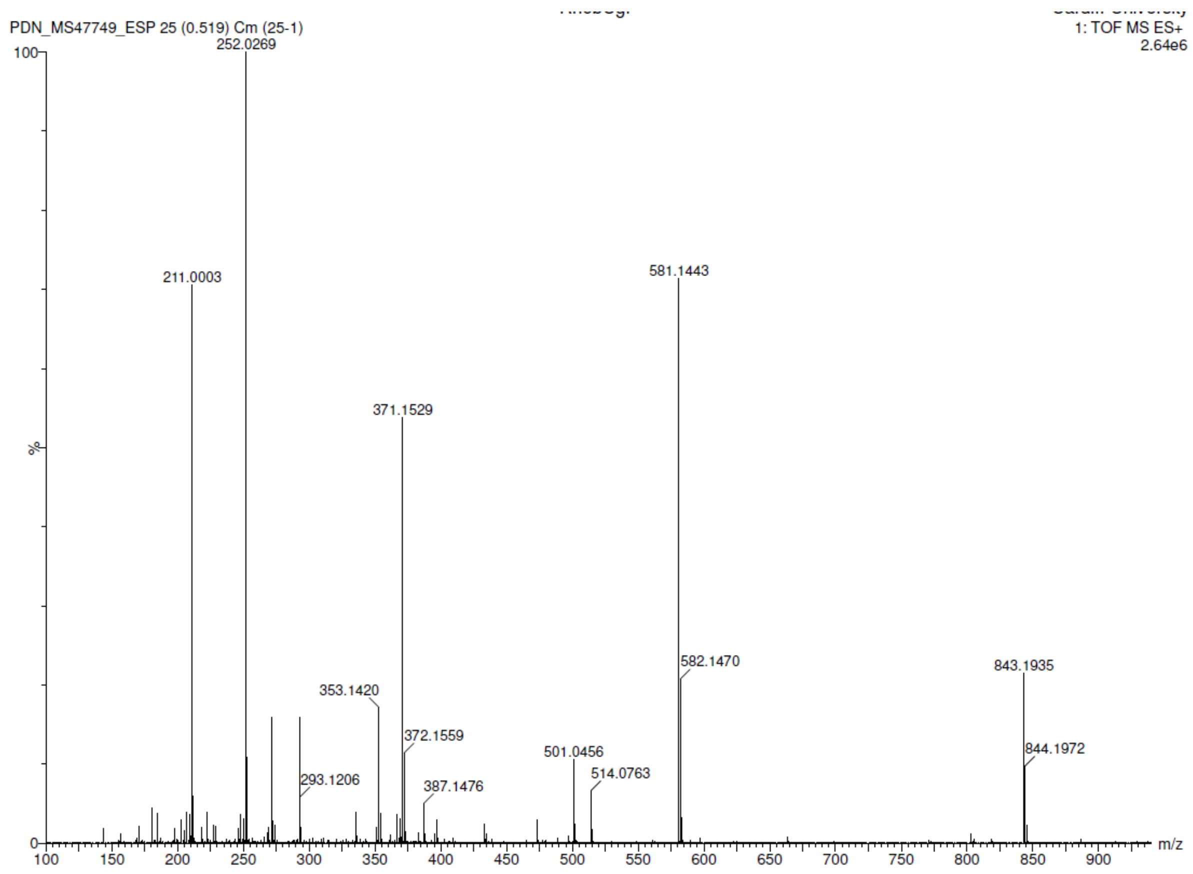

Minimum:				-1.5				
Maximum:		5000.0	10.0	50.0				
Mass	Calc. Mass	mDa	PPM	DBE	i-FIT	Norm	Conf(%)	Formula
581.1443	581.1440	0.3	0.5	13.0	736.4	n/a	n/a	C28 H35 N2 O3 P 103Rh

12. Reaction of [Rh(κ-*P*-**6L**)(COD)Cl)] with $[Re(CO)_5Cl]$.

Figure S77. $^{31}P\{^{1}H\}$ NMR spectrum in D_6-dmso at 162 MHz.

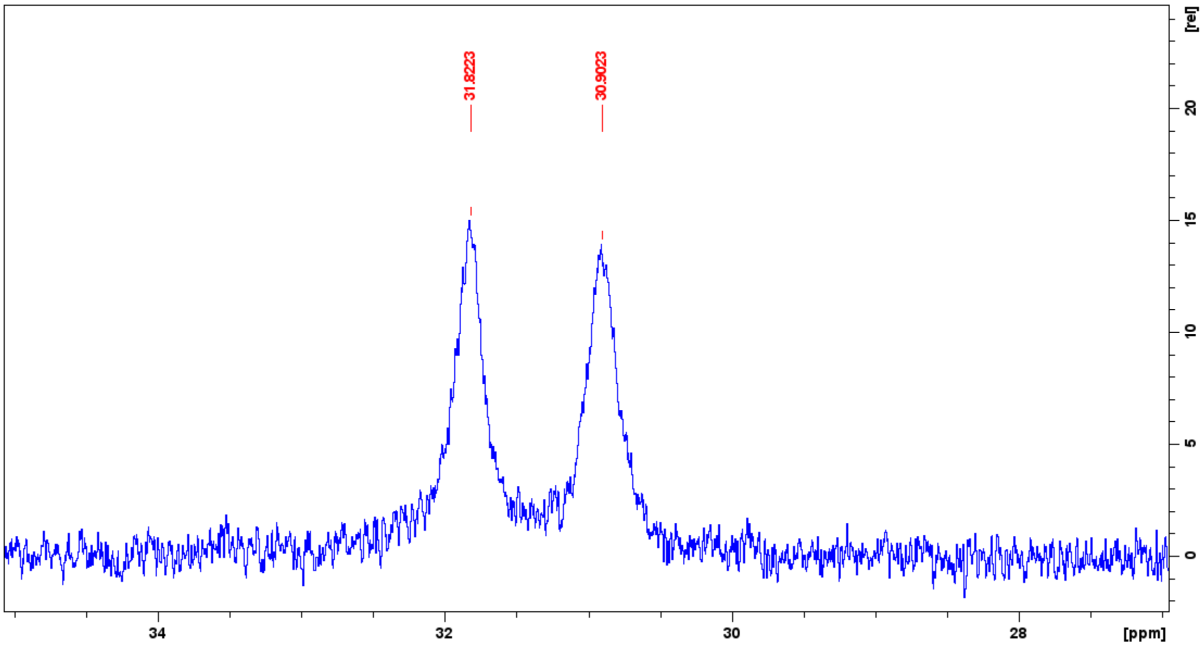

Figure S78. ^{1}H NMR spectrum in D_6-dmso at 400 MHz.

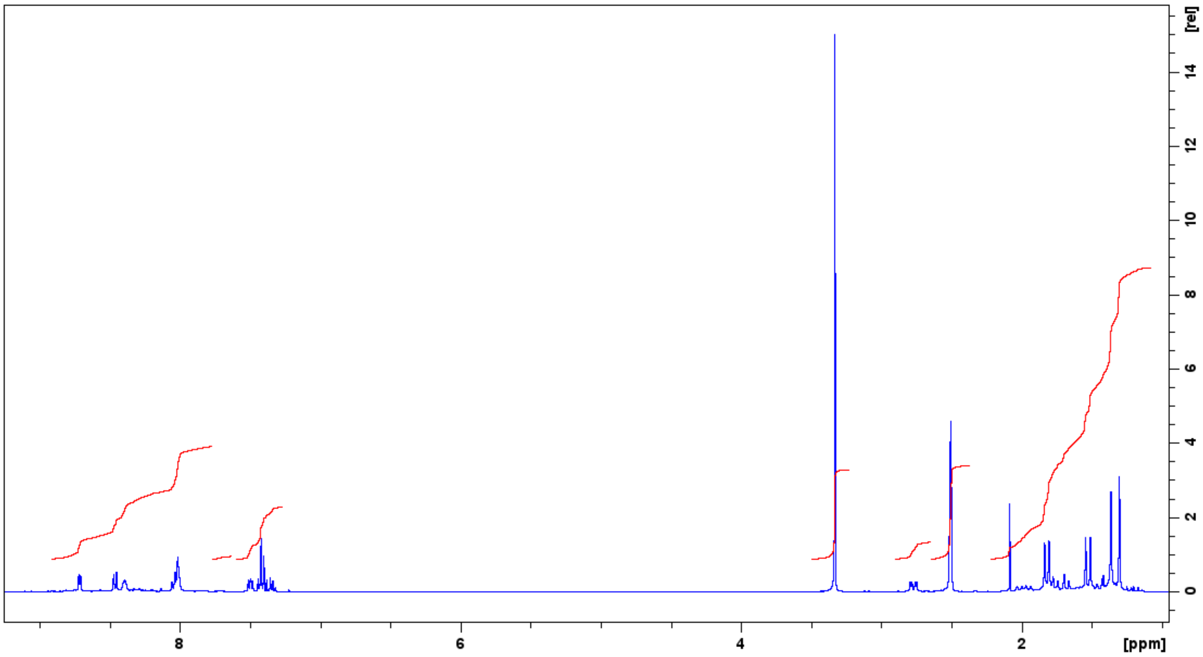

Figure S79. ^{1}H-^{1}H COSY NMR spectrum in D_6-dmso at 400 MHz.

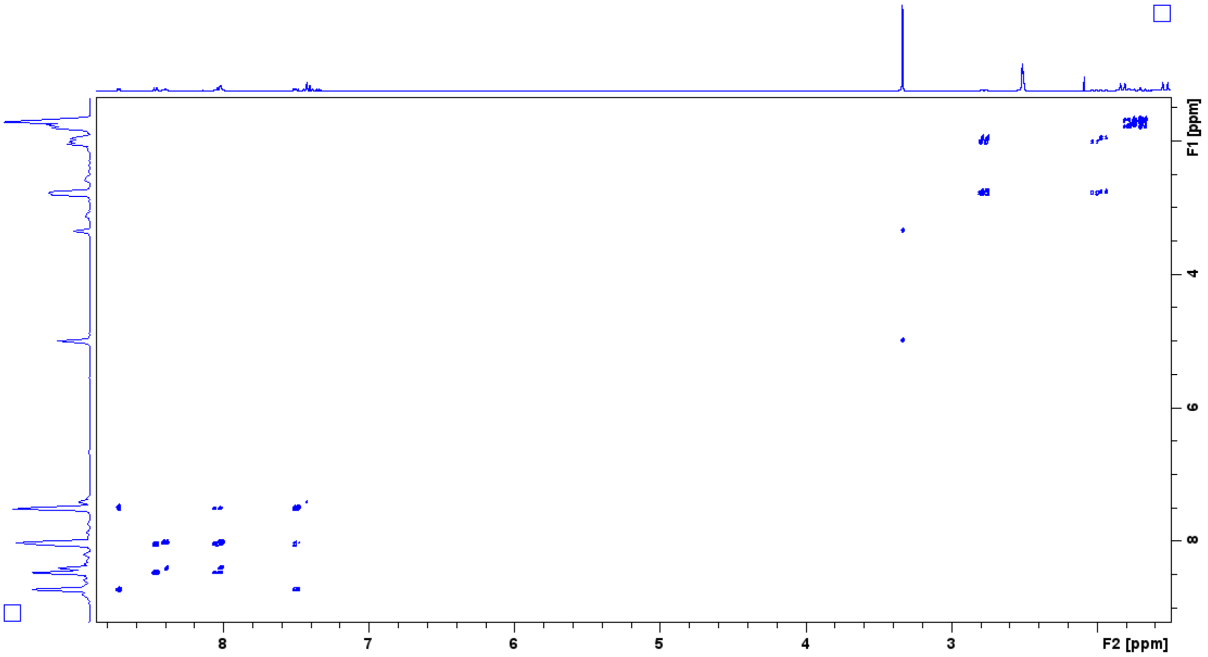

Figure S80. ^{1}H-^{13}C NMR spectrum in D_6-dmso.

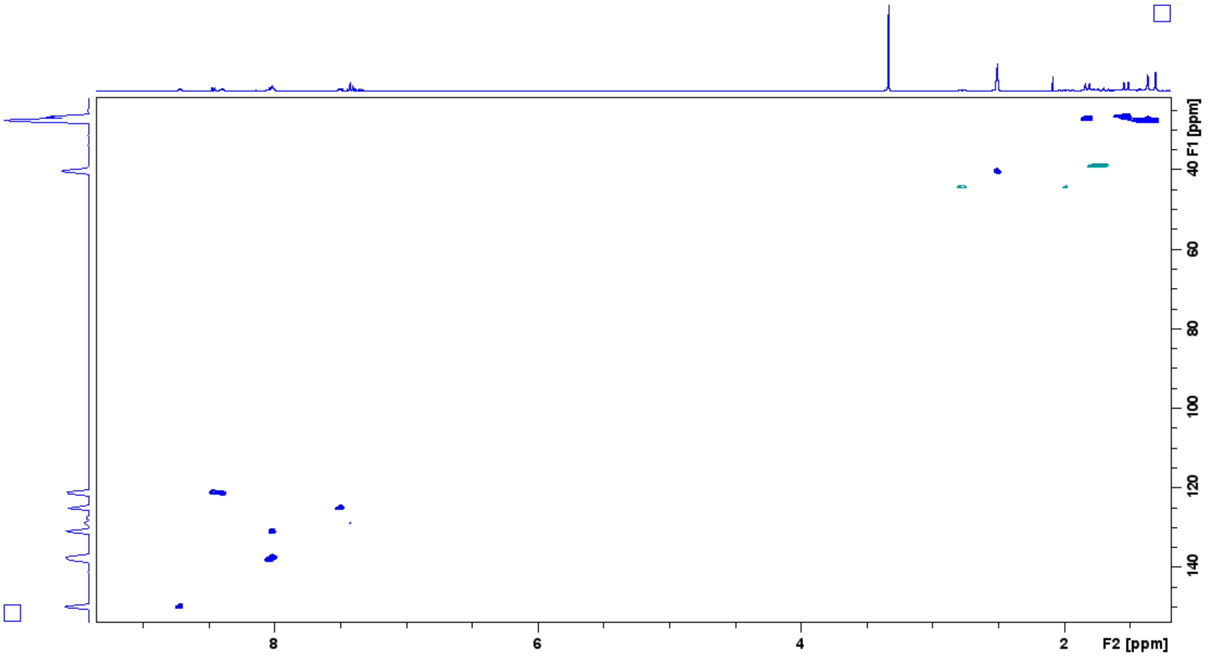

Figure S81. LRMS.

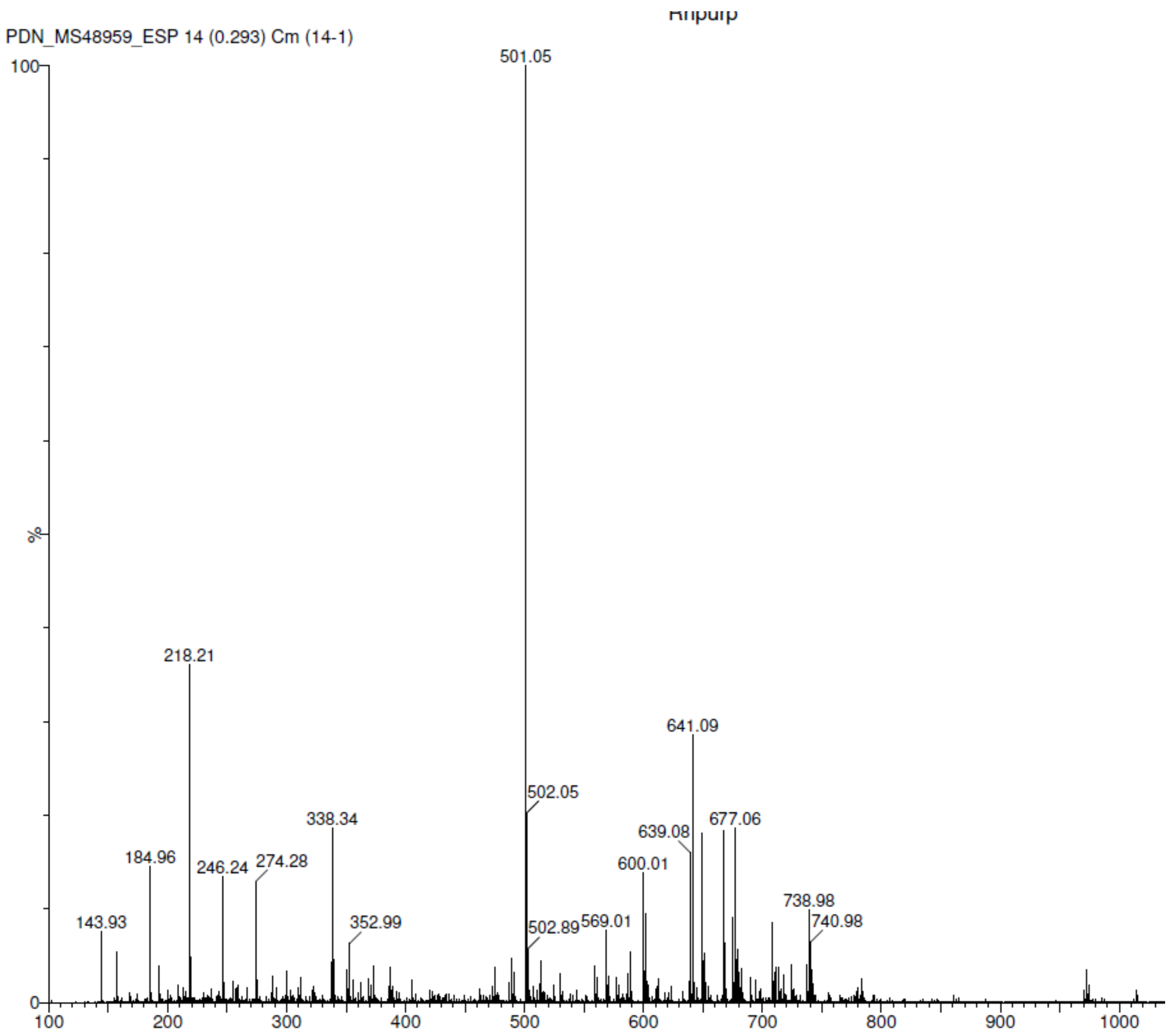

Figure S82. Electronic spectra of the complexes recorded in CH_2Cl_2 (1 x 10^{-5} M).

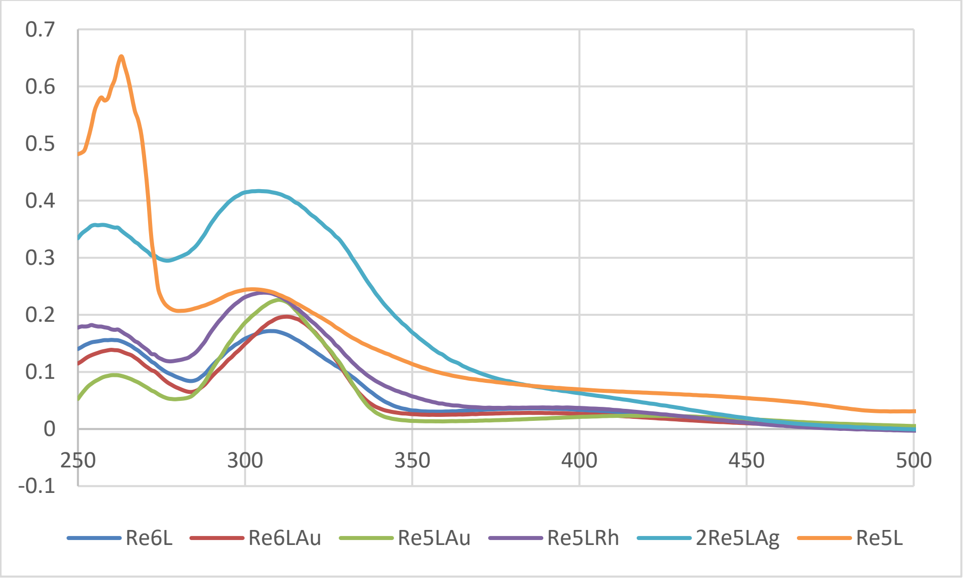

Figure S83. Normalised emission spectra of the complexes in CH_2Cl_2 (λ_{ex} = 410 nm)

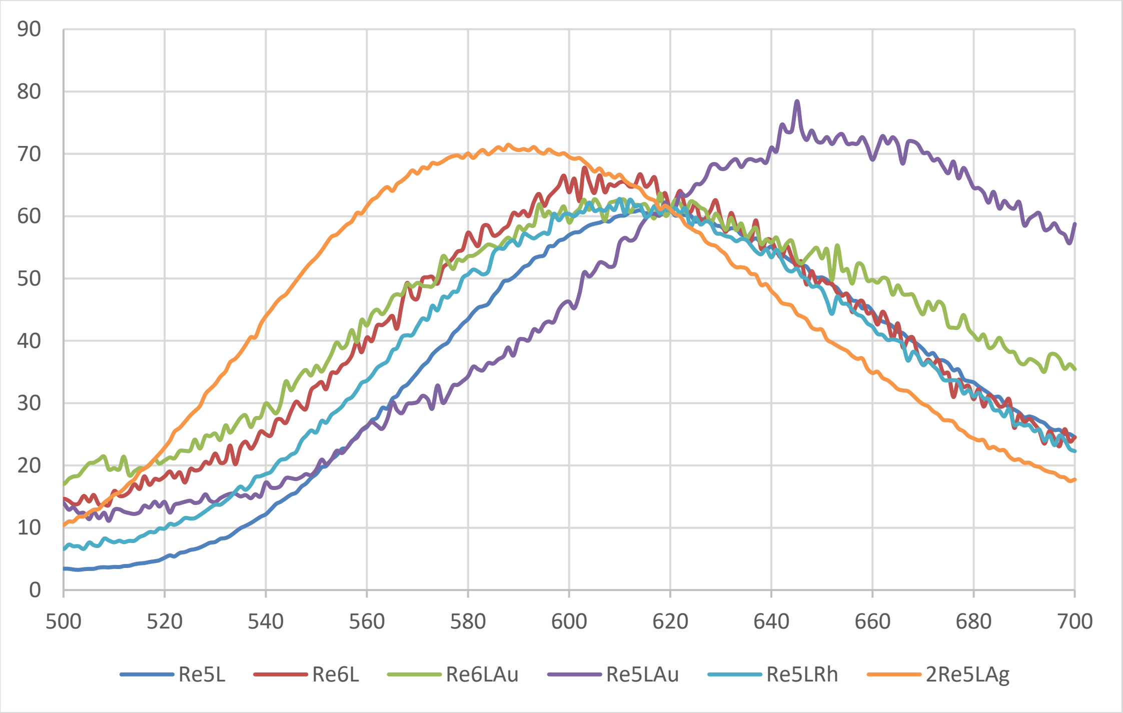

Table S1. Crystal data and structure refinement for **$5L^{Re,Au}$** and **$5L^{Re,Rh}$**.

Compound (Identification code)	**$5L^{Re,Au}$**	**$5L^{Re,Rh}$**
CCDC reference	2327744	2327745
Empirical formula	$C_{23}H_{23}AuCl_2N_2O_6PRe$	$C_{34}H_{41}Cl_2N_2O_7PReRh$
Formula weight	908.47	980.67
Temperature /K	200(2)	200(2)
Wavelength /Å	0.71073	0.71073
Crystal system	Monoclinic	Triclinic
Space group	P 2_1/n	P -1
a/Å	8.3545(2)	8.2303(3)
b/Å	24.9724(6)	13.2304(5)
c/Å	12.6864(3)	18.5876(5)
α/°	90	108.603(3)
β/°	95.620(2)	94.592(3)
γ/°	90	105.409(3)
Volume/Å^3	2634.07(11)	1818.96(11)
Z	4	2
Density (calculated)/ Mgm^{-3}	2.291	1.791

Absorption coefficient/ mm^{-1}	10.460	4.018
S1Crystal size/ mm^3	0.210x0.160x0.100	0.180x0.120x0.100
Reflections collected	6638	8986
Independent reflections	5741	7273
R(int)	0.0276	0.0358
Data / restraints / parameters	6638 / 0 / 329	8986 / 72 / 467
Goodness-of-fit on F^2	1.073	1.045
R1, wR2 [I>2σ(I)]	0.0346, 0.0741	0.0323, 0.0529
R1, wR2 (all data)	0.0441, 0.0792	0.0490, 0.0578
Largest diff. peak and hole e.Å^{-3}	1.986 and -1.956	1.706 and -1.063

5L^{Re,Au}.

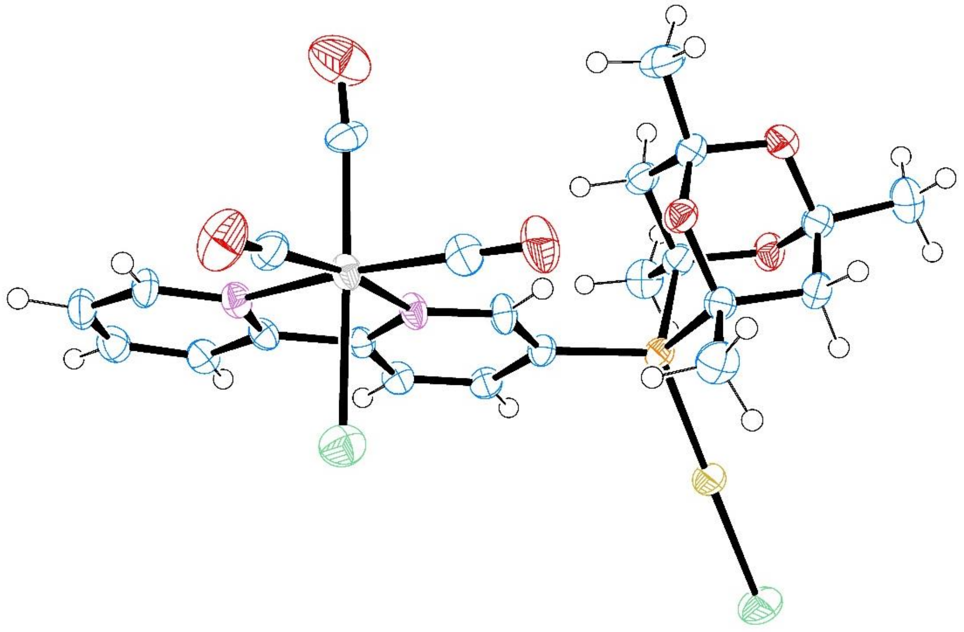

5L^{Re,Rh}.

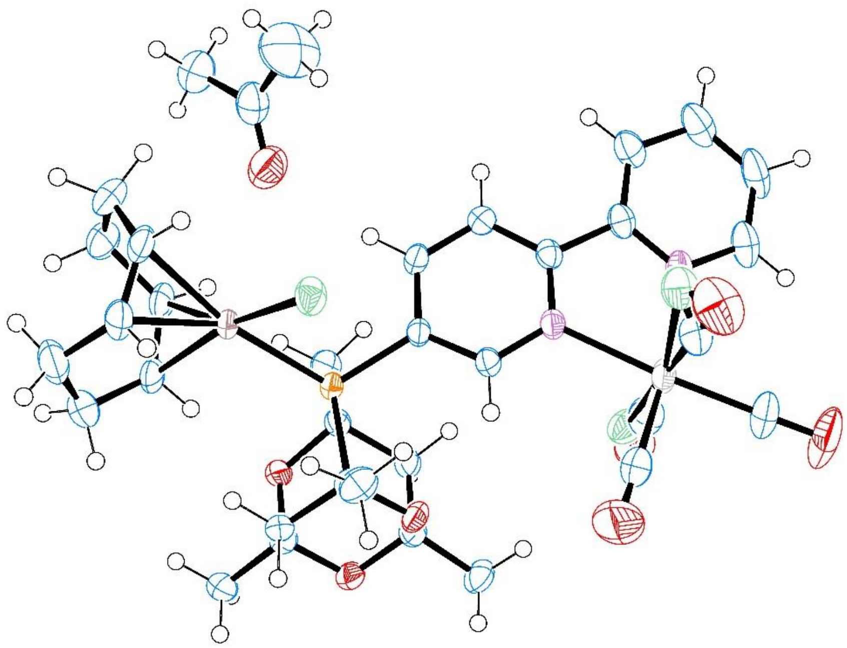

DFT data

DFT calculations were carried out using the Gaussian09 program. Rotational energy profiles and subsequent optimisations were calculated at BP86/def2SVP level, using density fitting with Automated fitting basis set assignment and D3 empirical dispersion correction. All minima were confirmed as such by harmonic frequency calculation. Subsequent calculation of orbital energies used B3LYP/def2TZVP level.

Table S2. Frontier molecular orbital energies

	**5L**			**6L**		
	HOMO	LUMO	Gap	HOMO	LUMO	Gap
Ligand only	-6.36	-1.91	4.45	-5.18	-2.43	2.75
Ligand-Re(CO)3	-5.85	-3.13	2.72	-4.86	-3.43	1.43
Ligand-AuCl	-6.69	-2.43	4.26	-5.46	-3.53	1.93
Ligand-Re(CO)3-AuCl	-6.09	-3.56	2.53	-5.05	-3.70	1.35

Optimised cartesian coordinates

5-bipyCgP

C	-1.966937	2.108746	1.931760
C	-0.977531	3.256975	3.674701
C	-0.106448	3.991695	2.827408
C	-0.196704	3.707519	1.444545
C	-1.123457	2.756180	0.997053
C	-2.988115	1.100568	1.497772
C	-4.241660	-0.762867	2.040876
C	-4.866609	-0.799985	0.779282
C	-4.516672	0.186249	-0.156052
C	-3.564950	1.151161	0.204947
H	-0.924615	3.414758	4.770034
H	0.463118	4.226413	0.732668
H	-1.174674	2.505161	-0.073818
H	-4.492850	-1.522832	2.804877
H	-5.608566	-1.578793	0.543185
H	-4.984933	0.210989	-1.153106
H	-3.291975	1.950602	-0.500739
C	2.430199	5.508164	2.363186
C	0.286276	6.901763	3.407897
C	3.264628	6.651728	2.976096
C	-0.053596	7.154518	1.932803
C	1.234606	7.214491	1.084888
C	2.446205	7.961440	2.985372
H	3.588787	6.395531	4.005662
H	4.169277	6.810324	2.350236
H	-0.563433	8.138769	1.846091
H	-0.742622	6.376885	1.546121
N	-3.332407	0.153663	2.398394
N	-1.880768	2.363756	3.255776
P	1.070375	5.149970	3.674549
O	1.919846	5.951315	1.082636
O	1.293246	7.846092	3.831487
O	2.066512	8.243383	1.632663
C	3.226747	9.163773	3.493633
H	4.124549	9.329140	2.867462
H	2.580287	10.061335	3.443157
H	3.534305	8.998855	4.544324
C	-0.906694	7.095679	4.335728
H	-1.307704	8.125832	4.225086
H	-1.712984	6.374022	4.092365
H	-0.603391	6.948784	5.391858
C	3.274567	4.265880	2.088651
H	2.662242	3.438519	1.678073
H	4.068519	4.511092	1.351739
H	3.752544	3.912456	3.024470
C	0.990940	7.569007	-0.374044
H	0.515706	8.566209	-0.446677
H	1.961105	7.591388	-0.907344
H	0.333213	6.815535	-0.849610

6-bipyCgP

C	-3.131189	-3.194226	-0.912148
H	-2.438364	-4.006681	-1.200629
C	-4.513528	-3.445055	-0.821065
H	-4.916129	-4.447966	-1.032157
C	-5.351587	-2.382324	-0.449122
H	-6.438993	-2.528914	-0.349679
C	-4.778565	-1.128009	-0.192833
H	-5.408855	-0.284062	0.126363

C	-3.376926	-0.973388	-0.316797
C	-2.712521	0.345668	-0.057812
C	-3.382217	1.569768	-0.282643
H	-4.405806	1.588475	-0.686176
C	-2.705690	2.767150	0.000158
H	-3.203824	3.737807	-0.154323
C	-1.391800	2.711104	0.482887
C	-0.772613	1.442941	0.633113
C	1.827012	3.219025	-0.715807
H	2.546839	3.431097	-1.534898
H	1.976899	3.963560	0.092241
H	0.803775	3.328658	-1.127380
C	2.053784	1.801911	-0.199147
C	3.496331	1.589640	0.309374
H	4.204986	1.944169	-0.470319
H	3.675995	2.165337	1.240453
C	3.753370	0.085302	0.547050
C	5.183575	-0.229224	0.960767
H	5.289527	-1.324634	1.083219
H	5.419051	0.265586	1.923045
H	5.891853	0.117170	0.183492
C	0.834906	-1.211788	2.458100
H	1.232365	-2.248659	2.494414
H	-0.260834	-1.251405	2.297499
H	1.049082	-0.718681	3.428098
C	1.491051	-0.451573	1.312231
C	1.262367	-1.114730	-0.050571
H	1.570107	-2.180637	0.027686
H	0.187508	-1.077429	-0.319524
C	2.148751	-0.454717	-1.119160
C	2.041891	-1.106476	-2.488644
H	1.003833	-1.025473	-2.864552
H	2.325589	-2.174574	-2.422713
H	2.726683	-0.592150	-3.190981
N	-2.572216	-2.000801	-0.668428
N	-1.438485	0.302033	0.385438
O	1.805978	0.929375	-1.309603
O	3.515030	-0.584835	-0.694987
O	2.914551	-0.423502	1.594000
P	0.954189	1.398564	1.346305
H	-0.848599	3.630938	0.750559

5-Re

C	-1.329190	2.058526	0.465978
C	0.486896	0.622465	0.862848
C	1.399832	1.690821	0.980096
C	0.862066	2.997830	0.916545
C	-0.497964	3.180845	0.651873
C	-2.741526	2.142758	0.048437
C	-4.626223	0.947072	-0.675254
C	-5.369726	2.119808	-0.841476
C	-4.767788	3.351798	-0.533193
C	-3.442454	3.359279	-0.086265
H	0.838895	-0.417316	0.904711
H	1.520244	3.872061	1.048136
H	-0.905656	4.196900	0.558150
H	-5.058131	-0.038384	-0.902745
H	-6.405118	2.059099	-1.206776
H	-5.323377	4.295467	-0.643014
H	-2.948461	4.309357	0.158935
C	3.533546	-0.358357	1.373842

C	3.744176	1.406648	-0.755852
C	5.049419	-0.544241	1.149312
C	2.908577	0.355086	-1.494486
C	3.206279	-1.056208	-0.950523
C	5.391401	-0.350187	-0.343162
H	5.634276	0.162512	1.773703
H	5.324715	-1.580897	1.439016
H	3.203205	0.364630	-2.566032
H	1.821631	0.569568	-1.453437
N	-3.346330	0.947629	-0.233493
N	-0.829944	0.795436	0.618541
P	3.240503	1.527163	1.126191
O	2.822378	-1.183661	0.433219
O	5.124976	1.007229	-0.747456
O	4.615714	-1.281937	-1.095044
C	6.852838	-0.606438	-0.674081
H	7.126593	-1.645347	-0.407344
H	7.005033	-0.461082	-1.761072
H	7.499374	0.100958	-0.119499
C	3.691838	2.781578	-1.409127
H	4.059769	2.709528	-2.454550
H	2.651275	3.164537	-1.433156
H	4.332372	3.500347	-0.859421
C	3.103618	-0.807986	2.769337
H	3.295907	-1.895662	2.879837
H	3.677347	-0.260465	3.543955
H	2.023804	-0.633221	2.947554
C	2.473846	-2.152341	-1.702292
H	2.853087	-2.201515	-2.741197
H	2.651387	-3.123012	-1.201087
H	1.386913	-1.938325	-1.727432
Re	-2.148704	-0.881773	0.075027
Cl	-1.143207	-0.408194	-2.167730
C	-3.424966	-2.143738	-0.652298
C	-0.921232	-2.351658	0.384846
C	-2.884035	-1.118138	1.846950
O	-4.226929	-2.857173	-1.118320
O	-0.165723	-3.220759	0.585019
O	-3.326254	-1.250914	2.925912

6-Re

C	4.403230	0.461851	1.221011
H	4.643546	-0.588245	1.443805
C	5.311045	1.489169	1.494270
H	6.282623	1.249943	1.950537
C	4.953924	2.809278	1.167142
H	5.642809	3.645506	1.361472
C	3.699244	3.046044	0.596145
H	3.391755	4.071939	0.352282
C	2.826665	1.965280	0.346632
C	1.450023	2.136103	-0.170789
C	0.963929	3.380770	-0.617816
H	1.632604	4.250550	-0.669627
C	-0.365569	3.485216	-1.034516
H	-0.752578	4.434605	-1.436485
C	-1.205258	2.369452	-0.903965
C	-0.685667	1.152366	-0.412884
C	3.235522	-2.248967	-0.148690
C	0.712698	-2.144379	-1.044239
C	-2.780669	-0.340224	-2.718011
H	-3.671479	-0.331007	-3.381132

H	-2.229783	-1.290516	-2.866249
H	-2.115430	0.494345	-3.018091
C	-3.221005	-0.209409	-1.263317
C	-4.140076	-1.367533	-0.820678
H	-4.966152	-1.469776	-1.556864
H	-3.576634	-2.322367	-0.787298
C	-4.758509	-1.058096	0.559496
C	-5.743857	-2.111951	1.038560
H	-6.160388	-1.799561	2.015707
H	-5.228869	-3.084425	1.159570
H	-6.570649	-2.220037	0.310467
C	-1.980425	0.136106	2.732719
H	-2.643414	0.300004	3.608888
H	-1.229974	0.952218	2.700347
H	-1.445204	-0.824162	2.866111
C	-2.813170	0.126797	1.457753
C	-3.595803	1.427925	1.242805
H	-4.160523	1.654981	2.173156
H	-2.912046	2.278068	1.047110
C	-4.619140	1.265850	0.100240
C	-5.506456	2.484420	-0.105028
H	-4.893752	3.375070	-0.346085
H	-6.092013	2.683188	0.813207
H	-6.203458	2.287339	-0.942381
N	3.193168	0.685234	0.655705
N	0.647961	1.024519	-0.134501
O	4.102183	-3.036511	-0.150245
O	0.070398	-2.907515	-1.651193
O	-3.959666	1.031259	-1.157455
O	-5.478108	0.175639	0.439670
O	-3.745133	-0.967415	1.576030
Re	1.875388	-0.887644	-0.112682
P	-1.715724	-0.370827	-0.071107
C	1.272140	-1.533685	1.604396
O	0.903882	-1.939686	2.644329
Cl	2.593835	0.048795	-2.310592
H	-2.267651	2.409830	-1.185544

5-Re-Au

O	2.306021	3.617994	0.876302
C	1.100775	2.869670	0.695507
C	1.073271	1.748296	1.754029
C	2.333182	0.884411	1.649085
O	3.504122	1.707876	1.617549
C	3.475713	2.819010	0.692103
C	3.482561	2.312136	-0.766713
C	2.180844	1.544817	-1.064060
O	1.050467	2.326434	-0.650091
P	2.314333	-0.030066	-0.024753
C	0.711724	-0.890496	-0.086163
C	-0.477643	-0.231708	-0.440905
N	-1.693935	-0.791880	-0.275048
C	-1.789986	-2.039646	0.278123
C	-0.633830	-2.796165	0.561521
C	0.628828	-2.222910	0.376015
C	-3.157648	-2.471893	0.618574
C	-3.473093	-3.762309	1.089287
C	-4.794028	-4.065872	1.442016
C	-5.774318	-3.063865	1.321753
C	-5.398584	-1.806169	0.835193
N	-4.125474	-1.511317	0.478922

C	2.009553	1.208688	-2.538013
C	2.503351	-0.115047	2.780462
C	-0.080809	3.808452	0.823586
C	4.680187	3.675488	1.029610
Re	-3.520116	0.422966	-0.400321
C	-4.055428	-0.202727	-2.152587
O	-4.368164	-0.598168	-3.211192
Cl	-2.692729	1.074971	1.869758
C	-5.215374	1.332735	-0.157362
O	-6.259311	1.820523	0.042993
C	-2.753992	2.024628	-1.183271
O	-2.249429	2.965784	-1.658828
H	-0.460820	0.800861	-0.817306
H	1.551949	-2.778330	0.613016
H	-0.724748	-3.811582	0.971730
H	-6.126190	-0.989534	0.716841
H	-6.823077	-3.247870	1.597888
H	-5.055163	-5.070202	1.809401
H	-2.684538	-4.523144	1.173894
H	4.369533	1.669797	-0.949332
H	3.523664	3.193835	-1.440788
H	1.071313	2.234194	2.752882
H	0.136804	1.157283	1.670992
H	4.706926	4.558913	0.361450
H	4.597871	4.012201	2.082185
H	5.606252	3.080399	0.902583
H	2.581513	0.432781	3.743569
H	1.624600	-0.791932	2.823073
H	3.424717	-0.714131	2.620037
H	1.967916	2.151051	-3.124047
H	2.869623	0.595638	-2.879504
H	1.071907	0.641161	-2.710661
H	0.010133	4.366751	1.776329
H	-0.086828	4.517945	-0.026924
H	-1.021011	3.220501	0.839331
Au	4.126417	-1.297632	-0.348681
Cl	6.021496	-2.570468	-0.662299

6-Re-Au

O	-3.632280	0.214641	1.547169
C	-4.678013	0.313518	0.557240
O	-5.131335	1.668228	0.432958
C	-4.082387	2.552034	0.053765
C	-3.018225	2.527966	1.171889
C	-2.512270	1.097849	1.405872
O	-3.505227	2.160243	-1.215581
C	-3.055329	0.794461	-1.327668
C	-4.180714	-0.136261	-0.830887
C	-2.685959	0.546355	-2.786187
P	-1.573435	0.422614	-0.150581
C	-0.226844	1.675220	-0.518809
C	-0.597880	2.926411	-1.053954
C	0.341637	3.968247	-1.110086
C	1.602700	3.761253	-0.553736
C	1.939751	2.480219	-0.071261
N	1.060905	1.423801	-0.134050
C	3.233081	2.218754	0.600169
C	4.082042	3.244880	1.065719
C	5.244088	2.915574	1.771729
C	5.529730	1.559666	2.013500
C	4.647435	0.590170	1.528662

N	3.528597	0.905131	0.832408
C	-5.835950	-0.513273	1.087710
C	-1.656249	0.945592	2.656289
C	-4.703679	3.921153	-0.172631
Re	2.283876	-0.502752	-0.256709
Cl	3.194620	0.725088	-2.234825
C	3.646132	-1.852560	-0.397651
O	4.513216	-2.634895	-0.456383
C	1.245851	-1.551430	-1.547043
O	0.740755	-2.124412	-2.427757
C	1.631445	-1.364079	1.351267
O	1.317169	-1.852773	2.369937
H	4.832714	-0.483519	1.680331
H	6.426658	1.249383	2.568983
H	5.914979	3.707527	2.138000
H	3.824405	4.298343	0.890628
H	2.346443	4.569126	-0.533478
H	0.073449	4.938880	-1.554785
H	-3.576741	0.717334	-3.425717
H	-2.343096	-0.499411	-2.919663
H	-1.877390	1.223207	-3.127227
H	-5.028494	-0.057246	-1.544218
H	-3.839654	-1.191175	-0.799602
H	-6.144731	-0.114040	2.073044
H	-5.522335	-1.568159	1.205377
H	-6.693236	-0.457798	0.389706
H	-2.254782	1.226167	3.548192
H	-0.765440	1.604002	2.603717
H	-1.315149	-0.102587	2.773004
H	-3.503227	2.876823	2.108609
H	-2.183137	3.220596	0.947283
H	-3.928373	4.658311	-0.458783
H	-5.207472	4.262628	0.751903
H	-5.452337	3.848537	-0.984892
H	-1.629295	3.073099	-1.405102
Au	-1.298328	-1.819015	0.114089
Cl	-1.387693	-4.102250	0.430056
